# Expansion of the 4-(Diethylamino)benzaldehyde Scaffold to Explore the Impact on Aldehyde Dehydrogenase Activity and Antiproliferative Activity in Prostate Cancer

**DOI:** 10.1021/acs.jmedchem.1c01367

**Published:** 2022-02-25

**Authors:** Ali I. M. Ibrahim, Elisabet Batlle, Smarakan Sneha, Rafael Jiménez, Raquel Pequerul, Xavier Parés, Till Rüngeler, Vibhu Jha, Tiziano Tuccinardi, Maria Sadiq, Fiona Frame, Norman J. Maitland, Jaume Farrés, Klaus Pors

**Affiliations:** † Institute of Cancer Therapeutics, School of Pharmacy and Medical Sciences, Faculty of Life Sciences, University of Bradford, Yorkshire BD7 1DP, U.K.; ‡ Faculty of Pharmacy, Al-Zaytoonah University of Jordan, Amman 11733, Jordan; § Department of Biochemistry and Molecular Biology, Faculty of Biosciences, Universitat Autònoma de Barcelona, Bellaterra, Barcelona E-08193, Spain; ∥ Department of Pharmacy, University of Pisa, Via Bonanno 6, 56126 Pisa, Italy; ⊥ Cancer Research Unit, Department of Biology, University of York, Heslington, Yorkshire YO10 5DD, U.K.

## Abstract

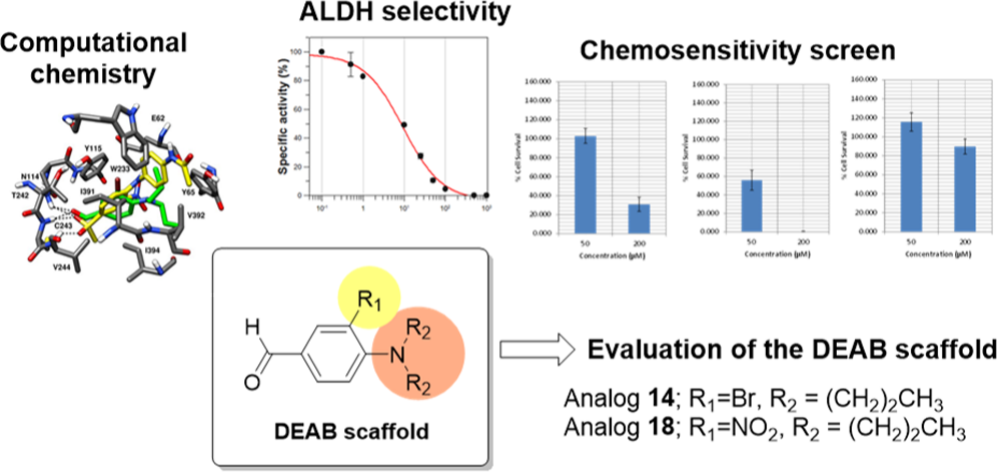

Aldehyde dehydrogenases (ALDHs) are overexpressed in various tumor types including prostate cancer and considered a potential target for therapeutic intervention. 4-(Diethylamino)benzaldehyde (DEAB) has been extensively reported as a pan-inhibitor of ALDH isoforms, and here, we report on the synthesis, ALDH isoform selectivity, and cellular potencies in prostate cancer cells of 40 DEAB analogues; three analogues (**14**, **15**, and **16**) showed potent inhibitory activity against ALDH1A3, and two analogues (**18** and **19**) showed potent inhibitory activity against ALDH3A1. Significantly, 16 analogues displayed increased cytotoxicity (IC_50_ = 10–200 μM) compared with DEAB (>200 μM) against three different prostate cancer cell lines. Analogues **14** and **18** were more potent than DEAB against patient-derived primary prostate tumor epithelial cells, as single agents or in combination treatment with docetaxel. In conclusion, our study supports the use of DEAB as an ALDH inhibitor but also reveals closely related analogues with increased selectivity and potency.

## Introduction

The aldehyde dehydrogenase (ALDH) superfamily of 19 different human isoforms is a group of NAD(P)^+^-dependent enzymes that catalyze several cellular processes, including detoxification of endogenous and exogenous aldehydes and biosynthesis of retinoic acid (RA), which is a modulator of stem cell (SC) differentiation.^1−3^ Recent interest in ALDHs emerges from their roles linked to cancer cell proliferation, differentiation, and survival^4,5^ and potential as markers of tumor-initiating cells or cancer SCs (CSCs).^6^

Identification of CSCs is frequently carried out using the Aldefluor assay, which includes the addition of 4-(diethylamino)benzaldehyde (DEAB), a small-molecule inhibitor that is used as a control to identify subpopulations of cells with high ALDH expression (ALDH^high^) and with SC-like properties.^4^ DEAB is a pan-ALDH inhibitor, which has been found to delay differentiation of CSCs,^7,8^ also showing potential in combination treatments with other drugs.^9−13^ Early work showed that 4-(dipropylamino)benzaldehyde was more potent than DEAB as a reversible inhibitor against mouse and human ALDH1, with variable inhibitory effects according to the selected substrate.^14^ In the same study, 4-(dimethylamino)benzaldehyde and 4-(dibutylamino)benzaldehyde showed low binding affinity to ALDH1 but provided no information on isoform selectivity.^14^ Many studies indicate that DEAB is a reversible and broad inhibitor of several ALDH isoforms, which is a confounding factor when using the Aldefluor assay.^15,16^ The complexity of the ALDH interaction is evident by recent work, which has shown that DEAB is also a substrate for ALDH1A1 and ALDH3A1,^15^ an irreversible inhibitor for ALDH1A2 and ALDH2, and neither a substrate nor inhibitor for ALDH1L1 and ALDH4A1, while it has been shown to be metabolized by ALDH1A3, ALDH1B1, and ALDH5A1.^17^ Additionally, DEAB has been shown to covalently bind to ALDH7A1, in which the DEAB-enzyme complex was successfully cocrystallized with the cofactor NAD^+^.^18^ It has been proposed that the structural features of the amino acid residues at the ALDH active sites are the main factors determining whether DEAB is a substrate or an inhibitor.^17^ Moreover, the ALDH–DEAB binary structure has been hypothesized to be stabilized by resonance arrangement, which is initiated and supported by the amine electron donors at the para position to the carbonyl group.^17^

ALDH1A1 and 1A3 are highly expressed in SC-like subpopulations and several cancer types.^8,19^ We have recently reported ALDH1A1 and 1A3 isoform expression in different prostatic tissue-derived cell lines (normal, benign, and malignant) and patient-derived primary prostate tumor epithelial cells and shown potential in inhibiting these for therapeutic intervention.^20^ Given the utility of DEAB in both identifying CSC populations^21^ and potential in treating such ALDH^high^-expressing populations,^8^ we decided to generate a new library of analogues that could be used to explore the DEAB scaffold to unravel key features. Here, we report on the synthesis, biological evaluation against ALDH1A1, 1A3, and 3A1, and antiproliferative activity as single agents against a panel of prostate cancer (PCa) cell lines. We also assessed selected agents for their potential in combination treatment with docetaxel.

## Results and Discussion

### Chemistry

**Scheme 1:**
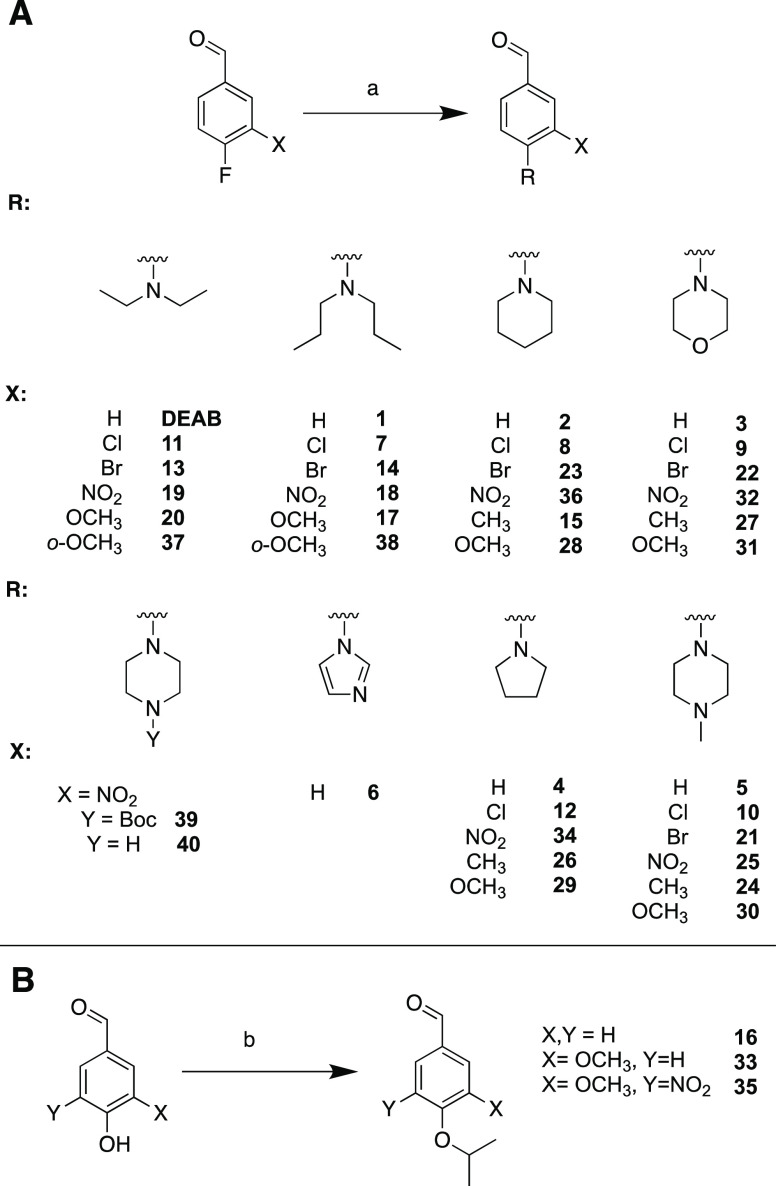
Nomenclature of DEAB Analogues and General Synthesis Scheme; Reaction Starts by a Nucleophilic Substitution at the Fluorine (A) or Hydroxyl Group (B) of the Substituted Benzaldehyde Standard conditions: (a) secondary amine, DMF, K_2_CO_3_, 25–100 °C and (b) 2-iodopropane, DMF, K_2_CO_3_, 25–90 °C, 6 h.

DEAB analogues were synthesized using either aliphatic or aromatic nucleophilic substitution one-step reactions. The nucleophilic aromatic substitution of fluorine by the desired secondary amine is shown in Scheme 1A. The presence of the aldehyde linked para to the fluorine group is likely facilitating the nucleophilic attack through electronic arrangement within the aromatic ring.^22−24^ The aldehyde is chemically reactive toward amines; however, as imines are less likely to be produced by secondary amines, the products were generally afforded in good yields (33–100%). Compounds **16**, **33**, and **35** were synthesized by the aliphatic nucleophilic substitution reaction between the phenolic hydroxyl group and isopropyl iodide, as shown in Scheme 1B.^25^ All DEAB analogues were synthesized, purified, and characterized by ^1^H NMR, ^13^C NMR, and HRMS as described in the Experimental Section.

### Inhibitory Effect of DEAB Analogues against ALDH Isoforms

Previous studies have established the importance of ALDH1 isoforms: 1A1^26^ and 1A3^27^ in CSCs and 3A1^28^ in drug resistance. Accordingly, we decided to explore a new library of DEAB analogues for interaction with these isoforms by generating structure–activity relationships (SAR) useful in informing DEAB properties critical to biological activity.

The inhibition screening and IC_50_ values were assayed using the conditions described in the Experimental Section. The IC_50_ value for compound **7** was measured at a saturating substrate concentration with ALDH3A1 because the same remaining activity was observed in inhibition screening both near the *K*_m_ value and at the saturating substrate concentration. Experimental values are shown as the mean ± SE. IC_50_ values and represent the concentration of the compound that decreases 50% the enzyme activity determined in the absence of the inhibitor. ND = not determined.

### ALDH1A1

Given the presence of the aldehyde group and the evidence that DEAB has been reported to be a slow ALDH1A1 substrate, we decided to perform both inhibition and substrate studies. Most compounds displayed lower inhibitory potency against ALDH1A1 compared with DEAB and were evaluated at an initial dose of 10 μM (Figure S1). Due to the significant substrate activity shown with many compounds, even much higher than that with DEAB (Figure S2), it was difficult to assess their inhibitory properties.

Accordingly, we captured full data analysis for compounds **14**, **26**, **29**, and DEAB, and IC_50_ values were calculated for the best fits, demonstrating the latter two compounds to be nearly equipotent with DEAB (IC_50_ = 0.48 ± 0.06 μM), while compound **14** was ∼15-fold less effective in inhibiting ALDH1A1 activity (Table 1). In general, those compounds with a better inhibition profile had dipropyl, diethyl, or a pyrrolidine as an R group, the latter being the best, especially compared to those with other groups such as morpholine or methyl-piperazine. Among those compounds that provided satisfactory results, unsubstituted analogues at the meta position (X group) yielded better inhibition results (measured as remaining activity at 10 μM compound) than those substituted with halogen atoms, that is, DEAB (*m*-H, 2%) versus **13** (*m*-Br, 24%), pyrrolidine **4** (*m*-H, 10%) versus **12** (*m*-Cl, 17%), or dipropylamine **1** (*m*-H, 12%) versus **14** (*m*-Br, 45%) (Figure S1). In fact, **14** yielded an IC_50_ of 7.08 ± 0.70 μM, approximately 15-fold less active when compared to DEAB as an inhibitor for ALDH1A1 (IC_50_ = 0.48 ± 0.06 μM). Contrary to ALDH1A3 and 3A1, the most potent ALDH1A1 inhibitors were analogues bearing an electron-donating group: **26** (*m*-CH_3_, IC_50_ = 0.80 ± 0.16 μM) and **29** (*m*-OCH3, IC_50_ = 0.88 ± 0.05 μM) (Table 1 1).

**Table 1:**
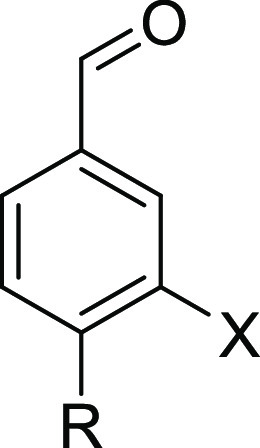

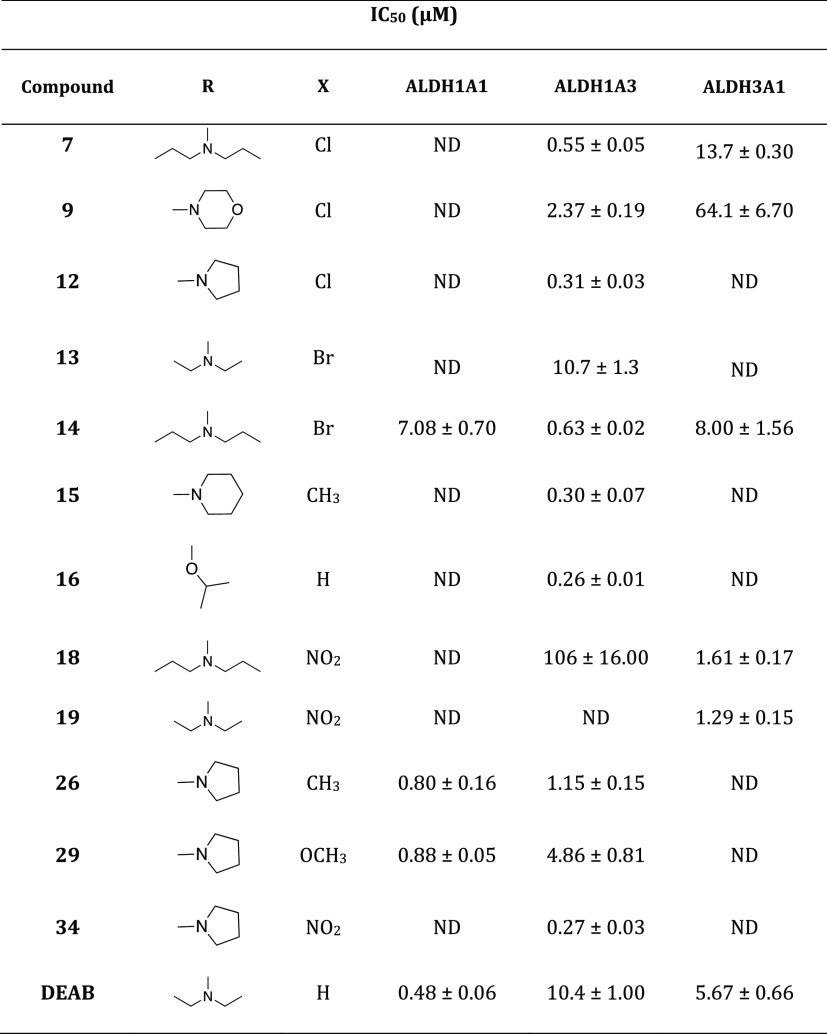
IC_50_ Values of the Most Potent DEAB Analogues against ALDH1A1, ALDH1A3, and ALDH3A1 Isoforms

### ALDH1A3

From the initial one-dose (10 μM) evaluation (Figure S1), the selection of compounds was based not only on inhibitory properties but also on their ability to act as substrates for the other two ALDH isoforms. Some compounds were shown to display very low IC_50_ values regarding ALDH1A3 (Table 1), yet its high percentage of substrate activity for ALDH1A1 and/or ALDH3A1 would complicate their further investigation and use as inhibitors. For example, **16** displayed the lowest IC_50_ value of all compounds in the library for ALDH1A3 (0.26 μM), yet it was shown to be a good ALDH3A1 substrate (Figure S2). Similarly, compounds **7** and **14** revealed comparable IC_50_ values (0.55 and 0.66 μM, respectively); however, **7** presented a significantly higher ALDH1A1 substrate activity compared to that of **14** (17.9 vs 3.14%). This result may be due to chlorine being a smaller atom compared to bromine, and thus, steric hindrance might explain why compound **7** can be better accommodated into the active site. Nonetheless, ALDH1A3 studies revealed many compounds to be superior to DEAB in inhibiting ALDH1A3 enzymatic activity with hexanal as a substrate (Table 1 and Figure S1).

There are several interesting SARs that reveal trends of inhibitory properties. Analogues bearing a methyl group at the meta position to the aldehyde and an aliphatic moiety at the para position reveal that the modulation of the latter from diethyl (DEAB) or dipropyl (**1**) to constrained heterocycles based on pyrrolidine (**26**) or piperidine (**15**) rings increases the inhibitory effect: **15** (IC_50_ = 0.29 μM) > **26** (IC_50_ = 1.15 μM) > DEAB (IC_50_ = 10.4 μM) ≈ **1** (only tested at 10 μM, Figure S1). Analogues incorporating para-positioned piperazine **24** and especially morpholine **27** also showed inhibitory capacity decreasing ALDH1A3 activity down to 45 and 10% at 10 μM dose, respectively. Pyrrolidine **12** with a chlorine installed at the meta position to the aldehyde was almost twice as potent (IC_50_ = 0.31 μM) as the propyl analogue **7** (IC_50_ = 0.55 μM), further substantiating the presence of a heterocycle at the para position for enhanced potent ALDH1A3 inhibitory activity. Interestingly, **34** was the only compound with an NO_2_ electron-withdrawing group that appeared effective at inhibiting ALDH1A3, with an IC_50_ = 0.27 μM. Comparison of propyl analogues **7** (*m*-Cl) and **14** (*m*-Br) revealed similar IC_50_ values (0.55 and 0.63 μM, respectively), indicating that the atomic volume and electronegativity of these halogens are not critical for ALDH1A3 inhibition.

Notably, comparison of diethyl **13** (IC_50_ = 10.7 μM) and diisopropyl **14** (IC_50_ = 0.63 μM) compounds revealed approximately 17-fold difference in capacity to inhibit ALDH1A3. This result suggests that the extra two methyl groups may provide additional nonpolar interactions with the active-site pocket, enough to strengthen binding. Figure S6 depicts the comparison of docking poses of **13** with **14** and DEAB within the ALDH1A3 binding pocket. Analogue **13** demonstrated a similar binding orientation as DEAB, establishing H-bonds with Cys313, Cys314, and Thr315. Both the *N*,*N*-diethyl side chains of **13** and DEAB established van der Waals contacts with Ile132 and Leu185 in the binding site of ALDH1A3, whereas analogue **14** was able to form only one H-bond with Thr315 and maintained van der Waals contacts with Ile132 and Leu185. Interestingly, the two extra methyl groups at the side chain of **14** were accommodated between Trp189 and Leu471 residues, favoring stronger van der Waals contacts than **13** and DEAB, thus improving its binding to ALDH1A3. Therefore, these findings may explain why **13** acted as an ALDH1A1 substrate with almost 60% activity relative to hexanal, whereas **14** only yielded 4% activity (Figure S2).

From all the compounds with a CH_3_ group at the meta position, compound **15** displayed the most potent inhibitory properties. Compound **16**, containing an isopropoxy group at the para position, was found to be one of the most potent compounds under the conditions investigated (IC_50_ = 0.26 μM). Significantly, several new analogues exhibited up to 100-fold higher potency in inhibiting ALDH1A3 as compared to DEAB (IC_50_ = 10.20 ± 2.15 μM) when using hexanal as a substrate.

**Figure 1:**
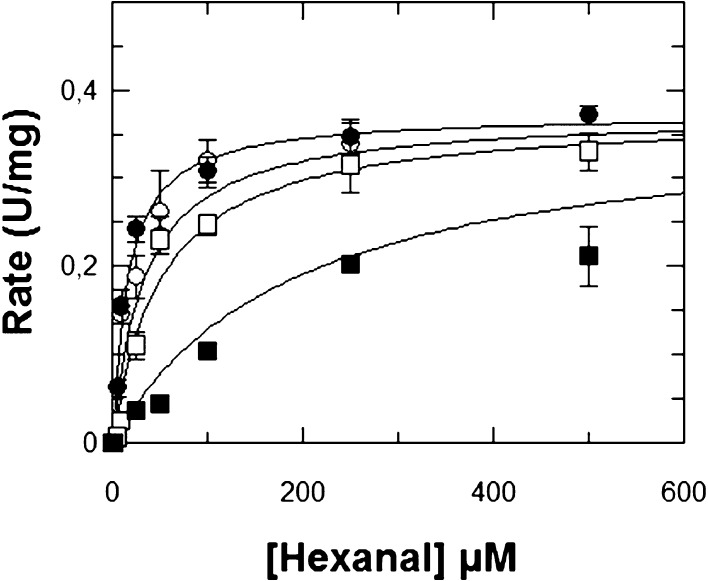
Inhibition kinetics of ALDH1A3 by compound **14** at various concentrations of inhibitor: −○– 0 μM; −●– 0.25 μM; −□– 2.5 μM; and −■– 5 μM. Hexanal was used as the substrate. The values of the kinetic parameters calculated from a fit to the competitive inhibition equation are *V*_max_ = 0.37 ± 0.01 U/mg; *K*_m_ = 16.1 ± 4 μM; and *K*_i_ = 0.46 ± 0.15 μM. Results are the mean ± SE of duplicate experiments.

The type of inhibition and *K*_i_ values determined for compounds **14**, **15**, and **16** against ALDH1A3 are shown in Figures 1, S4, and S5, respectively. It can be observed that when increasing the inhibitor concentration, the *K*_m_ value tends to increase, whereas the *V*_max_ value barely changes. This behavior best fits with a competitive type of inhibition. For these compounds, *Ki* values are all below 1 μM, in good agreement with the IC_50_ values. The calculated value for **14** was *K*_i_ = 0.46 ± 0.15 μM, which indicates that this compound is an excellent inhibitor for ALDH1A3.

With all observations taken together, it can be concluded that compound **14** yielded the most promising inhibition parameters while also displaying very low activity as a substrate for the ALDH1A1 and ALDH3A1 isoforms (lower than 5% at 10 μM for both isoforms).

### ALDH3A1

Compounds **14**, **18**, and **19** were shown to display IC_50_ values below 10 μM, with both **18** (IC_50_ = 1.61 μM) and **19** (IC_50_ = 1.29 μM) approximately threefold more potent than DEAB (IC_50_ = 5.67 μM). The latter two compounds share similar chemical structures, and the inhibitory activity is most likely related to three structural features: the lipophilicity of the para-substituted functional groups, the side chain flexibility involving free rotations, and the meta-substituted functional group. They differ in the para substitution groups diethylamine and dipropylamine. Compared to other analogues, these groups appear to be better accommodated in the active site and show more activity than those compounds with a similar *m*-NO_2_ group but with *p*-substituted groups of lower lipophilicity (i.e., morpholine **32** or pyrrolidine **34**).

It can be concluded that the NO_2_ group at the meta position seems to be providing these compounds with higher inhibitory activity. The docking analysis suggested that Tyr115, one of the ALDH3A1 residues that normally interacts with the CHO part of the molecule, presents some interaction with the NO_2_ group as well. This additional binding might be related to the better inhibitory capacity.

The only structural difference among **18** and **19** is the N-substituted group at the para position to the aldehyde, and although the results of the ALDH3A1 inhibition are very similar, their ability to act as substrates differs, especially with ALDH1A1 (17% of **18** vs 69% of **19**, Figure S2). Other para-substituted dipropylamine analogues (**1**, **7**, **14**, **17**, **18**, and **38**) were evaluated, but only those with electron-withdrawing groups demonstrated the capacity to significantly inhibit ALDH3A1 activity (below 15% remaining activity). Selectivity might be linked to a Gln122 residue, as a site for providing selectivity for ALDH3A1 only.^29^

**Figure 2:**
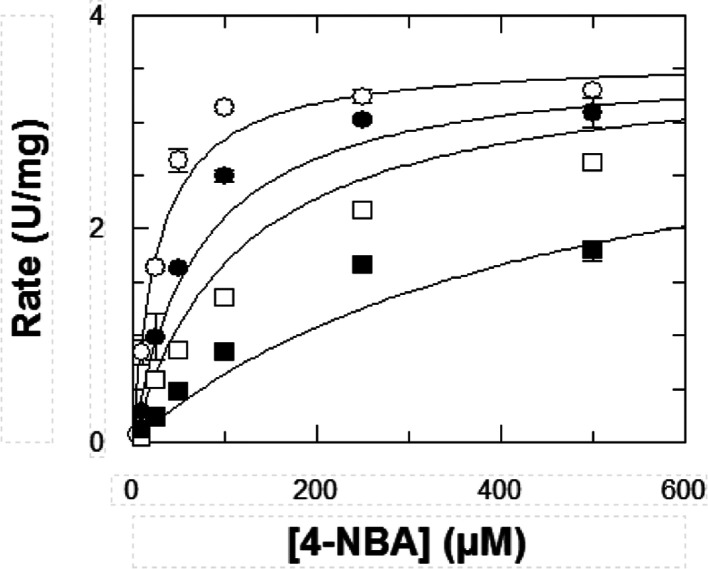
Inhibition kinetics of ALDH3A1 by compound 18 at various concentrations of inhibitor: −○– 0 μM; −●– 0.5 μM; −□– 1 μM; and −■– 5 μM. 4-Nitrobenzaldehyde (4-NBA) was used as the substrate. The values of the kinetic parameters calculated from a fit to the competitive inhibition equation are *V*_max_ = 3.59 ± 0.16 U/mg; *K*_m_ = 26.92 ± 5.41 μM; and *K*_i_ = 0.30 ± 0.06 μM. Results are the mean ± SE of duplicate experiments.

**Figure 3:**
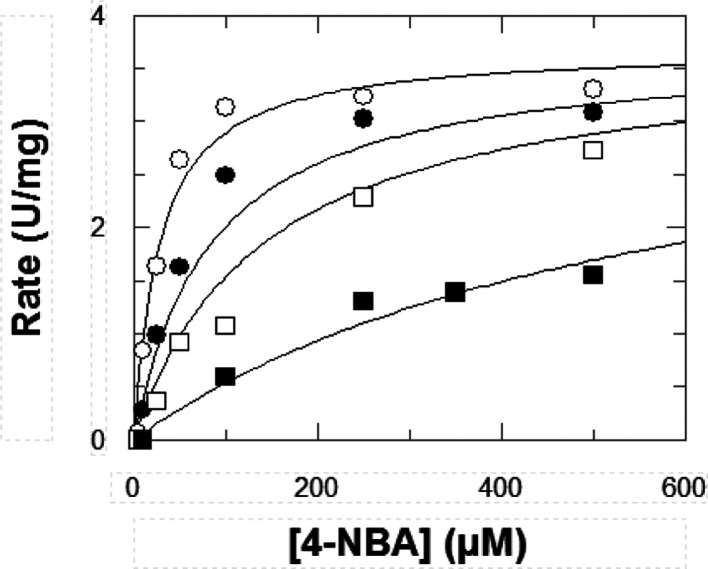
Inhibition kinetics of ALDH3A1 by compound 19 at various concentrations of inhibitor: −○– 0 μM; −●– 0.5 μM; −□– 1 μM; and −■– 5 μM. 4-NBA was used as the substrate. The values of the kinetic parameters calculated from a fit to the competitive inhibition equation are *V*_max_ = 3.69 ± 0.16 U/mg; *K*_m_ = 27.81 ± 5.36 μM; and *K*_i_ = 0.24 ± 0.04 μM. Results are the mean ± SE of duplicate experiments.

Kinetic evaluation of both **18** and **19** revealed them as excellent candidates for ALDH3A1 inhibition. Both compounds were further characterized as ALDH3A1 inhibitors by determination of the type of inhibition and *K*_i_ value. Data are shown in Figures 2 and 3. Compounds were best fitted to a competitive type of inhibition. The calculated value for *Ki* was 0.3**0** ± 0.**06** μM for **18** and 0.24 ± 0.04 μM for **19**, which indicates that compounds **18** and **19** are excellent inhibitors of ALDH3A1 functional activity.

**Figure 4:**
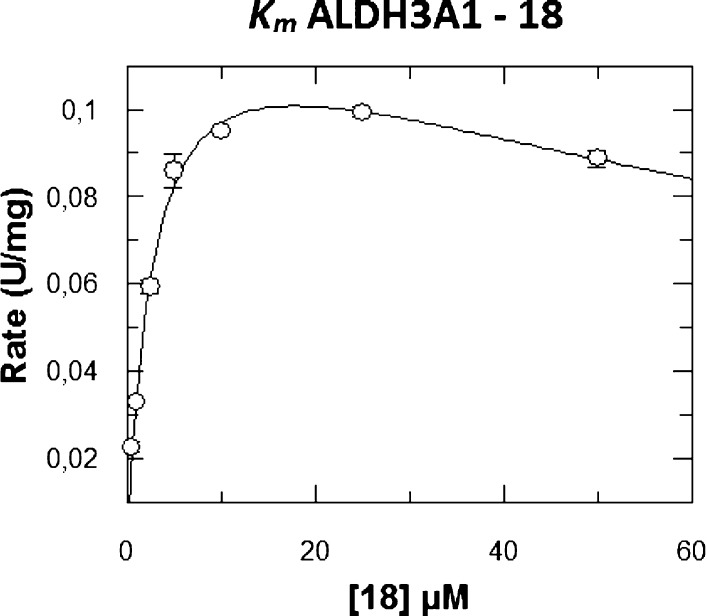
*K*_m_ value for ALDH3A1 using compound **18** as a substrate. Experimental values were fitted to the substrate inhibition equation and the kinetic values were *K*_m_ = 2.82 ± 0.35 μM, *K*_si_ = 113 ± 25 μM, and *k*_cat_ of 13.4 ± 0.7 min^–1^. Data were the result of duplicate experiments and expressed as the mean ± SE.

Additionally, we decided to further investigate the role of **18** as an ALDH3A1 substrate (4.21% with 10 μM, Figure S2) to assess whether it would interfere with the inhibition experiments. Kinetic analysis of the saturation profile for **18** with ALDH3A1 was best fitted to the Michaelis–Menten equation modified for substrate inhibition (Figure 4), producing a *K*_m_ of 2.82 ± 0.35 μM and a *K*_si_ of 113 ± 25 μM. For comparison, the *K*_m_ of the best ALDH3A1 substrate, 4-NBA, is 10-fold higher, with a *K*_m_ of 31.0 ± 4.9 μM. Regarding *k*_cat_, 4-NBA has a 26-fold higher turnover number (359 ± 14 min^–1^) than compound **18**, which has a *k*_cat_ value of 13.4 ± 0.7 min^–1^. These findings suggest that **18** performs better as an inhibitor than as an ALDH3A1 substrate. We also performed two different IC_50_ calculations for compound **18**. The aim was to check whether **18** was acting as a substrate during the standard 5-min preincubation time, when NADP^+^ and the enzyme were added but with no standard substrate in the reaction mixture. One IC_50_ was calculated by incubating with the cofactor and the other was calculated without it, and no differences were observed (Figure S6), indicating that the function of compound **18** as an inhibitor was not being affected by its role as a substrate under the assayed conditions.

### Antiproliferative Activity of DEAB Analogues in Prostate Cancer Cell Lines

**Figure 5:**
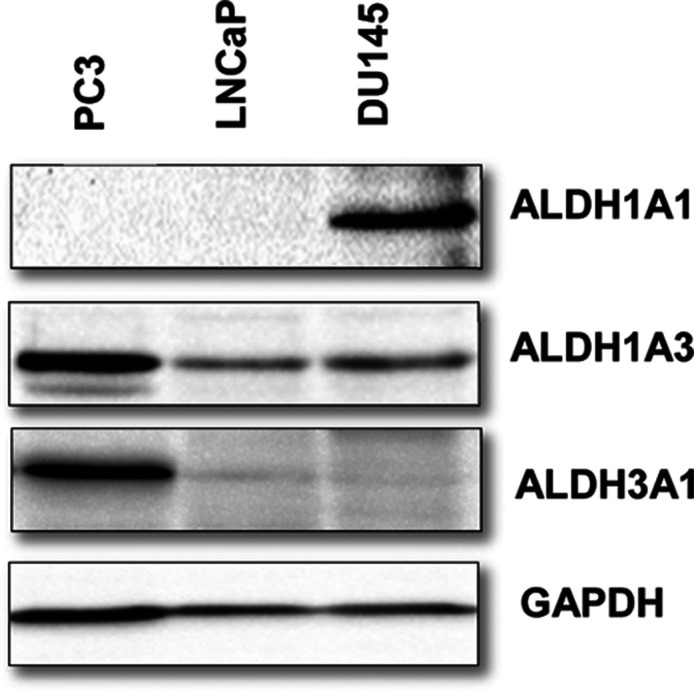
Immunoblot analysis was carried out using ALDH1A1, 1A3, and 3A1 specific antibodies in a panel of immortalized PCa cell lines (PC-3, LNCaP, and DU145) and using GAPDH as a control.

Several members of the ALDH family of enzymes have been shown to be expressed in PCa,^30−34^ and ALDH1A1 and 1A3 isoforms have been reported to be expressed at higher levels in tumor tissues compared to benign prostatic hyperplasia and normal prostate.^35^ ALDHs have also been acknowledged to promote clonogenic and migration cell capabilities *in vitro* and enhance the metastatic potential *in vivo*,^34,36^ while the expression correlates with a higher Gleason score (G8-9) *in vivo*.^20,31,35^ We have previously shown the importance of the RA pathway in PCa^37,38^ and ALDHs as a potential target,^20^ and in this context, it was suitable to explore the new DEAB library of compounds. Due to their ALDH expression profile (Figure 5), we chose to use a small panel consisting of the three prostate cancer cell lines PC3, DU145, and LNCaP.

All compounds were initially assessed using the MTT assay in an initial two-dose point screen (96 h exposure) to identify hit compounds. The results revealed clear dose-dependent trends (Figure S14), which, together with observations from the biochemical screening, led to 17 compounds including DEAB to be further investigated in a five-dose screen.

All DEAB analogues displayed IC_50_ values in the micromolar range (10–200 μM) as measured using the MTT assay. Most compounds showed equipotent antiproliferative activity in PC_3_ (expressing ALDH1A1 and 3A1) and DU145 (expressing ALDH1A1) cell lines and increased potency against LNCaP (expressing ALDH1A3) cells (Table 2); the cellular potency is likely to be multifactorial and not just a direct correlation of the ALDH isoform expression. Nonetheless, the dipropyl moiety appeared to provide compound **14** with a higher antiproliferative potency than diethyl-based analogue **13** and piperidine analogue **23** across all three cell lines. These findings were also found for compound **18** with the dipropyl moiety, which showed higher antiproliferative activity than diethyl analogue **19**. Therefore, analogues **14** and **18** were shown to be the most promising compounds in agreement with data obtained from the biochemical screening. In addition, compound **14** showed the highest antiproliferative activity in both DU145 and PC_3_ cell lines with IC_50_ values of 61 and 47 μM, respectively; this correlated with the biochemical assay IC_50_ data obtained for ALDH1A1 (7.08 μM), ALDH1A3 (0.63 μM), and ALDH3A1 (8.00 μM).

**Table 2:** Antiproliferative Effect of DEAB Analogues Against PC3, LNCaP, and DU145 PCa Cell Lines

	IC_50_ (μM)		
Cmpd	PC3	LNCaP	DU145
1	>200	73 ± 14	190 ± 25
6	172 ± 30	137 ± 14	133 ± 11
7	77 ± 17	10 ± 3	90 ± 27
8	130 ± 9	37 ± 1.4	110 ± 3
13	166 ± 41	47 ± 0.6	123 ± 12
14	47 ± 6	25 ± 1	61 ± 5
15	123 ± 29	61 ± 14	106 ± 13
16	>200	>200	>200
17	106 ± 25	61 ± 18	103 ± 4
18	98 ± 12	31 ± 6	100 ± 13
19	>200	136 ± 13	>200
21	>200	179 ± 14	187 ± 14
22	196 ± 4	82 ± 10	155 ± 32
23	84 ± 6	46 ± 10	84 ± 12
26	>200	172 ± 8	>200
38	75 ± 10	50 ± 3	71 ± 11
DEAB	>200	>200	>200

IC_50_ values represent the concentration of the compound that decreases cell survival by 50%. Results are expressed in μM ± SD and derived from at least three independent experiments.

Cell line sensitivity after treatment with ALDH-targeting compounds might not only be a reflection of the ALDH expression and functional activity. Previous reports have demonstrated that there is a striking difference in the metabolic phenotypes of the three cell lines used in this study.^39^ Due to a mitochondrial dysfunction, PC3 and DU145 cells have been shown to have an increased glycolytic reliance, unlike LNCaP which is highly oxidative, which might contribute to ALDH activity and compound sensitivity. Resistance mechanisms might contribute to compound sensitivity. For example, the PC3 cell line represents the type of cancer that is difficult to treat as it is an androgen-independent metastatic prostate cancer type.^20^ Nevertheless, apart from compound **16**, it was noticeable that all analogues selected for chemosensitivity screening displayed more potent antiproliferative effects when compared with DEAB.

**Figure 6:**
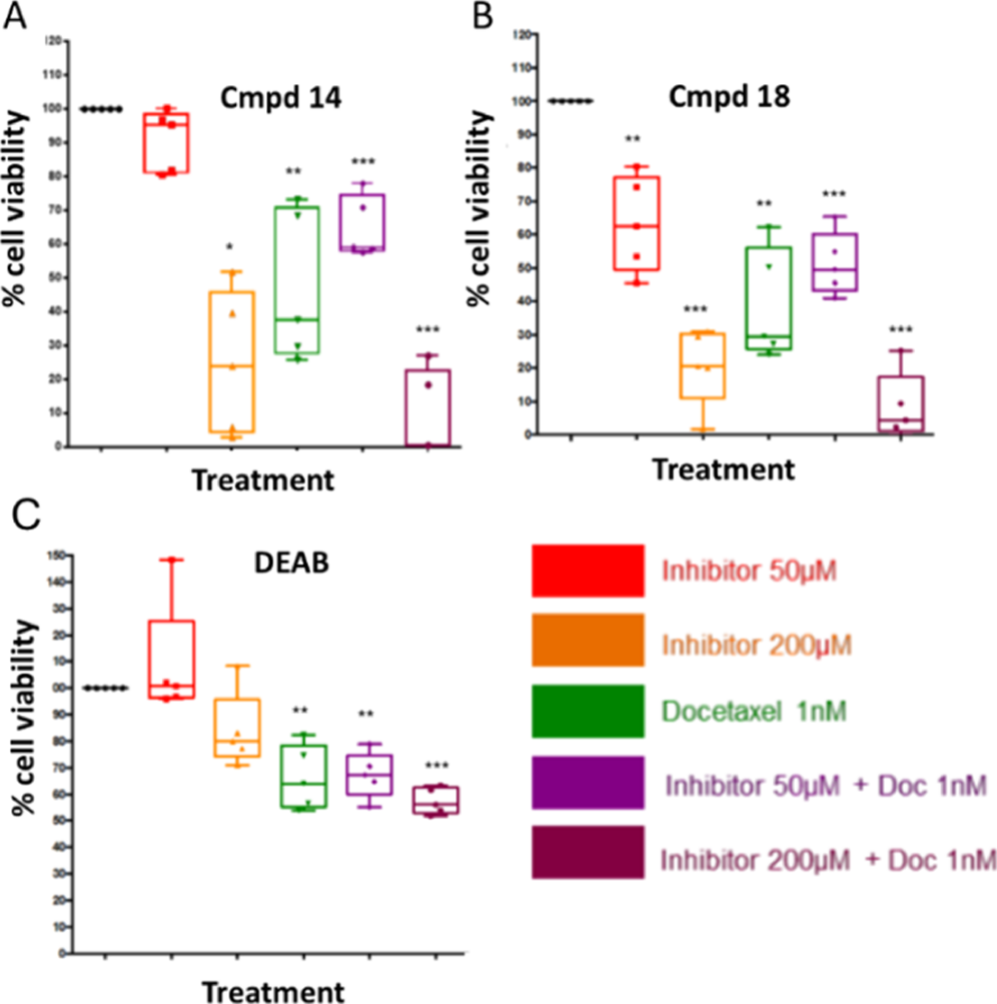
Cell viability of primary cells following treatment with **14** (**A**), **18** (**B**), and DEAB (**C**) as a single or combination treatment with docetaxel. Patient samples assessed included BPH sample H415/15 and cancer samples H568/15 RM, H431/14 LM, H488/14 RM, and H517/15 RM. The experiment was carried out in triplicate and values are represented as the mean. Statistical significance was calculated using a paired two-tailed Student’s *t*-test, in which the mean of untreated cells was compared with the mean of treated cells, *p < 0.05, **p < 0.01, and ***p < 0.001, combination treatment with docetaxel.

ALDHs have also been linked to chemo- and radioresistance in cancer therapy.^40,41^ An increased expression of ALDH can have a chemoprotective effect on cells due to their metabolic and detoxifying abilities [reviewed in ref (41)]. The taxanes paclitaxel and docetaxel have been reported to be less effective in ALDH-expressing cells.^42−44^ Given that docetaxel is the most commonly used cytotoxic drug for the treatment of advanced PCa, we next decided to evaluate three compounds (**14**, **18**, and DEAB) as single agents and in combination with docetaxel against five patient samples (four cancers with Gleason score 7 and one BPH) to investigate the potential of such a combination in clinical samples. The compounds were evaluated using two different concentrations (50 and 200 μM), and results revealed dose-dependent reduction in percentage cell viability of primary prostate epithelial cultures (Figure 6A–C). In line with the PCa cell line data, analogues **14** and **18** were more potent than DEAB, a pattern also evident in the combination with docetaxel (1 nM). To gain further insights into the observations obtained from both the biochemical assays and chemosensitivity experiments, docking studies were performed for compounds **14** and **18**, as they showed promising results.

### Docking Studies of Compounds **14** and **18** on ALDH1A1, ALDH1A3, and ALDH3A1 Isoforms

**Figure 7:**
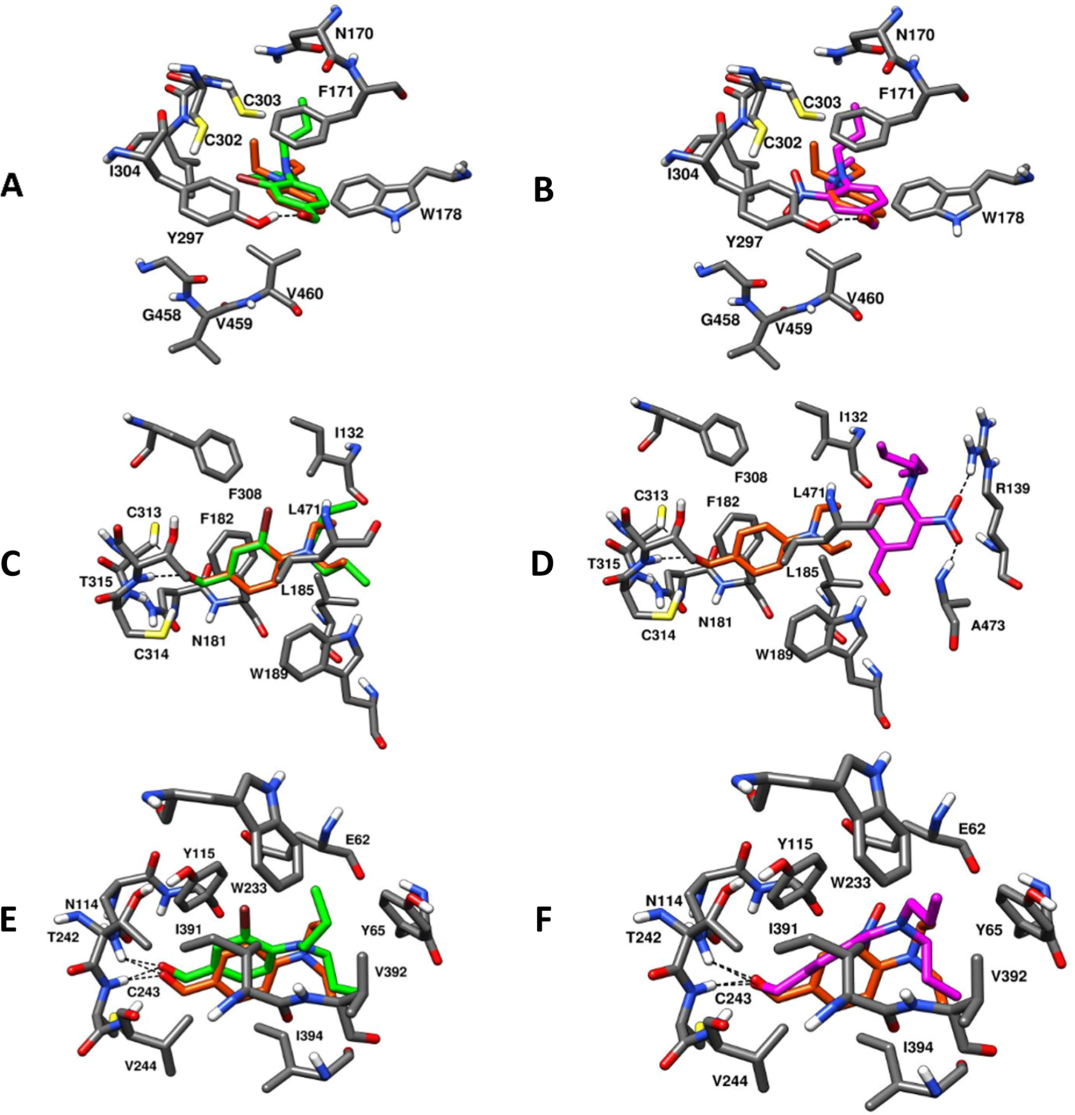
Molecular docking of compounds **14** (green) and **18** (magenta) into ALDH1A1 (A,B, PDB ID: 4WPN), ALDH1A3 (C,D, PDB ID: 5FHZ), and ALDH3A1 (E,F, PDB ID: 4H80) binding sites. Best fit binding modes are compared to DEAB (orange).

Analogues **14** and **18** were docked into the three investigated isoforms (ALDH1A1, 1A3, and 3A1), with all analysis shown in Figures S7–S13. In addition, binding modes with DEAB were included to compare its binding affinity to these three analogues, as shown in Figure 7. From the docking study of analogue **14** with the ALDH1A1 binding site (Figure 7A), the aldehyde oxygen of **14** was found to form an H-bond with Tyr297, and the phenyl ring established face-to-face π–π stacking with Phe171. The electron-rich *m*-Br group was found to be in close proximity of Phe171, Tyr297, and Ile304, capable of constituting van der Waals contacts with these residues. One of the propyl groups of the N-substituted side chain established van der Waals contacts with Trp178. Compound **14** was found to superimpose on DEAB, maintaining the abovementioned protein–ligand interactions. Compound **18** (Figure 7B) displayed similar protein–ligand interactions to **14** and DEAB, in the binding pocket of ALDH1A1. However, the presence of *m*-NO_2_ in the place of an *m*-Br group did not result in an H-bond interaction within the binding site.

As depicted in Figure 7C, analogue **14** occupied the binding pocket of ALDH1A3 with high affinity through several intermolecular forces: the aldehyde oxygen formed a H-bond contact with Thr315, the *m*-Br phenyl ring established van der Waals interactions with Phe182, Trp189, and Leu471, and the electronic interactions between bromine and both Phe308 and Ile132 residues significantly contributed to enhanced binding affinity with the target, which may explain the results obtained in the ALDH1A3 biochemical studies (Table 1). In addition, compound **14** was found to be superimposed with the binding disposition of DEAB, altogether contributing to the improved binding affinity of **14** for ALDH1A3. Analogue **18**, as shown in Figure 7D, did not properly occupy the ALDH1A3 binding site, justifying the poor/inactive potencies against the ALDH1A3 isoform obtained from the biochemical studies. The presence of the *m*-NO_2_ group in **18**, as an H-bond acceptor, completely altered the binding disposition of this analogue within the enzyme binding site, by forming an H-bond network at the entrance of the ALDH1A3 binding pocket with Arg139 and Ala473 residues, thus restricting their deeper entry into the active site.

Both ligands constituted the fundamental H-bonds with Cys243 and Asn114 of ALDH3A1 (Figure 7E,F). In particular, compound **18** exhibited a preference binding for ALDH3A1 when compared with 1A1 and 1A3 isoforms. Apart from H-bond contacts, both analogues displayed significant van der Waals interactions in the ALDH3A1 binding pocket; the phenyl ring of **14** was observed to establish an edge-to-face π–π stacking with Tyr115 and van der Waals contact with the Ile394 side chain. The *m*-Br group of **14** was found to be in proximity of Ile391, thus favoring the electronic interaction, and one of the propyl of the N-substituted side chain formed a hydrophobic contact with Tyr65. Figure 7F also shows that the phenyl ring of ligand **18** formed a face-to-face π–π stacking with Tyr315 and the N-substituted side chain provided a van der Waals contact with Tyr65. In addition, the docking results of **14** and **18** were compared with those for DEAB in the ALDH3A1 binding pocket (Figure 7E,F). DEAB was superimposed on the binding orientation of **14** within the ALDH3A1 binding site, exhibiting identical H-bond and hydrophobic interactions. The phenyl ring of DEAB formed an edge-to-face π–π stacking with Tyr115 and van der Waals contacts with Ile394, whereas the ethyl group from one of the *N*-ethyl side chains of DEAB established another van der Waals contact with Tyr65. The phenyl ring of **18** was oriented differently relative to the phenyl ring of **14**.

## Conclusions

DEAB has been shown to be effective in treating aggressive ALDH^high^-expressing subpopulations with SC-like propensity *in vitro* and features as a key component of the Aldefluor assay used to identify cancer cells with SC properties. In regard to the former, it is possible that the inclusion of a therapeutic agent aimed at eradicating prostate CSCs could increase the overall survival rate.^19,45−47^ In PCa, patients who no longer respond to androgen therapy (ADT) develop an aggressive disease known as castrate-resistant prostate cancer (CRPC), which has high propensity for metastasis and short median survival rates ranging from 12.1 to 27.0 months.^48−50^ Currently, drugs that are used to treat advanced PCa include small-molecule-based therapies such as the androgen receptor (AR) inhibitor enzalutamide,^51^ the CYP17A1 inhibitor abiraterone acetate,^51^ and the taxanes docetaxel^52^ and cabazitaxel.^53^ Unfortunately, these therapies are rarely curative, necessitating the identification of new molecular targets and/or development of therapeutic strategies to treat aggressive PCa. In this study, we wanted to (i) generate a DEAB library to help understand ALDH isoform selectivity and (ii) investigate the potential of treating PCa cells as single agents and combination with docetaxel. Accordingly, we synthesized a small library of 40 compounds with a benzaldehyde scaffold and evaluated them against three ALDH isoforms—1A1, 1A3, and 3A1—known to be expressed in PCa. The results showed that an electron-donating group (amine or oxygen) at the para position to the aldehyde group is important for activity, particularly when connecting with a lipophilic carbon chain that participates in van der Waals interactions with ALDH active sites. In addition, the presence of an electron-withdrawing group at the meta position to the aldehyde also was found to increase affinity to the binding site. The synthesized compounds showed promising inhibitory properties, notably identifying compounds **14**–**16**, **18**, and **19** with superior activity against ALDH1A3 and 3A1 isoforms when compared with DEAB.

The antiproliferative activity of the compounds was also evaluated in three PCa cell lines, and interestingly, most compounds outperformed DEAB in cellular potency. We further investigated DEAB and analogues **14** and **18** in combination with docetaxel in primary PCa cells from tumors with a high Gleason score of 7. Although the results are only indicative, they do suggest that docetaxel treatment of aggressive primary PCa cells might benefit from the inclusion of an ALDH inhibitor such as compound **14** or **18**. In uterine endometrial cancer, paclitaxel treatment has been shown to increase the proportion of ALDH^high^ cells in clinical samples and in spheroids.^54^ Spheroids are enriched in CSCs, which mainly depend on an enhanced glycolytic metabolic pathway for their proliferation and survival. This glycolytic activation is mediated by the expression of ALDH and its crucial downstream effector GLUT1. Interestingly, the combination of ALDH/GLUT1 inhibitors with paclitaxel has been shown to suppress the proliferation of endometrial cancer cells in a synergistic manner, indicating that ALDH-dependent GLUT activation might be relevant for the maintenance of chemoresistance of CSCs.

In conclusion, further mechanistic studies are required to fully understand how inhibition of ALDH activity might be linked to potentiation of docetaxel treatment in PCa. Given the well-established capacity of DEAB to inhibit ALDH^high^-expressing CSCs, it also remains to be explored whether analogues from our DEAB library, such as **14** and **18**, can be used as chemical probes to further unravel the significance of ALDH expression in CSCs and possible link to taxane resistance. An additional outcome of the present work might indicate that the Aldefluor assay specificity could be improved using compounds **14** and **15** to label ALDH1A3-expressing cells or **18** and **19** for ALDH3A1-expressing cells, given their increased selectivity as compared with DEAB.

## Experimental Section

### General Procedures

All materials and reagents were used as received with no further purification. 4-Fluoro-3-nitrobenzaldehyde, 3-cyano-4-fluorobenzaldehyde, 2-bromo-4-fluorobenzaldehyde, and 4-fluoro-2-methoxybenzaldehyde were purchased from fluorochem, 3-bromo-4-benzaldehyde, 4-fluorobenzaldehyde, 4-fluoro-3-methoxybenzaldehyde, 3-chloro-4-fluorobenzaldehyde 4-fluoro-3-methylbenzaldehyde, diethylamine, dipropylamine, morpholine, piperidine, pyrrolidine, DMSO, and 4-diethylaminobenzaldehyde (DEAB) were purchased Sigma-Aldrich; 1-methylpiperazine and 5-nitrovanillin were purchased from Acros Organics. Chemical reactions were monitored by analytical thin-layer chromatography using Merck 9385 silica gel 60 F254 aluminum-backed plates through visualizing the spotted plates under ultraviolet (UV) at 254 and 366 nm. Intermediates and final products were purified by column chromatography using silicagel 60A 40–63 μm. Proton and carbon NMR spectra were analyzed for all intermediates and final products on a Bruker AMX400 (400 MHz) nuclear magnetic resonance spectrometer. Chemical shifts were reported in parts per million (δ, ppm) downfield from internal TMS. Coupling constants (*J*) were expressed in Hertz (Hz). High-resolution mass spectra were obtained by the Engineering and Physical Sciences Research Council (EPSRC) mass spectrometry service, Swansea. Melting points were measured with a Gallenkamp melting point apparatus. All compounds biologically evaluated were >95% pure by HRMS/HPLC analysis except analogue 40 (82%); HPLC traces for all compounds can be found in the Supporting Information.

### General Procedures for the Synthesis of Compounds 1–15, 17–32, 34, 36–38

To a stirred solution of starting aldehyde (1 equiv) in DMF (10 mL), the corresponding amine (6.0 equiv) and K_2_CO_3_ (2.0 equiv) were added and stirred at (25–100) °C for hours–days. The reaction was then cooled to room temperature, and DMF volume was reduced by evaporation under vacuum. Water (30 mL) was then added to the mixture and stirred for 30 min. The mixture was then extracted with ethyl acetate (EA) (3 × 20 mL), and the organic fractions were combined, washed with water, and dried with MgSO_4_. EA was then evaporated under vacuum to give the product as crude, which was purified by silica gel column chromatography, affording the desired targeted compounds.

### General Procedures for the Synthesis of Compounds 16, 33, and 35

To a stirred solution of the starting aldehyde (1 equiv) in DMF (10 mL), iodopropane (4.0 equiv) and K_2_CO_3_ (2 equiv) were added and stirred at 90 °C for 6 h. The reaction was then cooled to room temperature, and DMF volume was reduced by evaporation under vacuum. Water (30 mL) was then added to the mixture and stirred for 30 min. The mixture was then extracted with EA (2 × 20 mL), and the organic fractions were combined, washed with water, and dried with MgSO_4_. EA then evaporated under vacuum to give the product as crude, which was then purified by silica gel column chromatography, affording the desired targeted compounds.

### 4-(Dipropylamino)benzaldehyde (1)

Starting reagents were 4-fluorobenzaldehyde and dipropylamine, processed at 100 °C for 48 h. The crude was purified by column chromatography using 5% EA in petroleum ether (PE), affording 56% of the title product as a yellow oil. *R*_f_ = 0.26 (EA/PE, 1:10). ^1^H NMR (400 MHz, CDCl_3_): δ 9.72 (s, 1H), 7.72 (d, 2H, *J* = 8.9 Hz), 6.67 (d, 2H, *J* = 8.9 Hz), 3.40–3.25 (t, 4H, *J* = 7.2 Hz), 1.78–1.53 (m, 4H), 0.98 (t, 6H, *J* = 7.4 Hz). ^13^C NMR (101 MHz, CDCl_3_): δ 190.0, 152.5, 132.2, 124.8, 111.0, 53.0, 20.3, 11.4. HRMS (ESI): calcd for C_13_H_19_NO [M + H]^+^, 206.1539; found, 206.1535.

### 4-(Piperidin-1-yl)benzaldehyde (2)

Starting reagents were 4-fluorobenzaldehyde and piperidine, processed at 100 °C for 24 h. The crude was purified by column chromatography using EA/PE (0.05:10) to afford 66.7% of the title product as a yellow solid. *R*_f_ = 0.13 (EA/PE, 1:10). mp 70 ± 1 °C. ^1^H NMR (400 MHz, CDCl_3_): δ 9.66 (s, 1H), 7.64 (d, 2H, *J* = 8.9 Hz), 6.81 (d, 2H, *J* = 8.9 Hz), 3.32 (m, 4H), 1.59 (m, 6H). ^13^C NMR (101 MHz, CDCl_3_): δ 190.3, 155.1, 132.0, 126.2, 113.3, 48.4, 25.3, 24.3. HRMS (ESI): calcd for C_12_H_15_NO [M + H]^+^, 190.1226; found, 190.1221.

### 4-Morpholinobenzaldehyde (3)

Starting reagents were 4-fluorobenzaldehyde and morpholine, processed at 100 °C for 48 h. The crude was purified by column chromatography using 10% EA in petroleum ether, affording 56.6% of the title product as a yellow solid. *R*_f_ = 0.47 (EA/PE, 1:1). mp 63 ± 1 °C. ^1^H NMR (400 MHz, CDCl_3_): δ 9.73 (s, 1H), 7.70 (d, 2H, *J* = 8.9 Hz), 6.85 (d, 2H, *J* = 8.9 Hz), 3.78 (t, 4H, *J* = 4.9 Hz), 3.27 (t, 4H, *J* = 4.9 Hz). ^13^C NMR (101 MHz, CDCl_3_): δ 190.6, 155.2, 131.8, 127.6, 113.5, 66.5, 47.3. HRMS (ESI): calcd for C_11_H_13_NO_2_ [M + H]^+^, 192.1019; found, 192.1015.

### 4-(Pyrrolidin-1-yl)benzaldehyde (4)

Starting reagents were 4-fluorobenzaldehyde and pyrrolidine, processed at 100 °C for 48 h. The crude was purified by column chromatography using EA/PE (0.5:10) to afford 87.5% of the title product as a yellow solid. *R*_f_ = 0.31 (EA/PE, 2:10). mp 92 ± 1 °C. ^1^H NMR (400 MHz, CDCl_3_): δ 9.73 (s, 1H), 7.74 (d, 2H, *J* = 8.8 Hz), 6.60 (d, 2H, *J* = 8.8 Hz), 3.40 (t, 4H, *J* = 6.6 Hz), 2.07 (t, 4H, *J* = 6.6 Hz). ^13^C NMR (101 MHz, CDCl_3_): δ 190.3, 151.9, 132.2, 124.9, 111.4, 47.8, 25.4. HRMS (ESI): calcd for C_11_H_13_NO [M + H]^+^, 176.1070; found, 176.1065.

### 4-(4-Methylpiperazin-1-yl)benzaldehyde (5)

Starting reagents were 4-fluorobenzaldehyde and 1-methylpiperazine, processed at 90 °C for 24 h. The crude was purified by column chromatography using (CH_3_OH/CH_2_Cl_2_, 0.5:10) to afford 96.7% of the title product as a yellow solid. *R*_f_ = 0.49 (CH_3_OH/CH_2_Cl_2_, 1:10). mp 74 ± 1 °C. ^1^H NMR (400 MHz, CDCl_3_): δ 9.73 (s, 1H), 7.70 (d, 2H, *J* = 8.9 Hz), 6.87 (d, 2H, *J* = 8.9 Hz), 3.40–3.31 (m, 4H), 2.58–2.45 (m, 4H), 2.31 (s, 3H). ^13^C NMR (101 MHz, CDCl_3_): δ 190.3, 155.0, 131.8, 127.0, 113.5, 54.6, 47.0, 46.1. HRMS (ESI): calcd for C_12_H_16_N_2_O [M + H]^+^, 205.1335; found, 205.1331.

### 4-(1*H*-Imidazole-1-yl)benzaldehyde (6)

Starting reagents were 4-fluorobenzaldehyde and 1*H*-imidazole, processed at 100 °C for 24 h. The crude was purified by column chromatography using (CH_3_OH/CH_2_Cl_2_, 0.1:10) to afford 38.9% of the title product as a pale-yellow solid. *R*_f_ = 0.29 (CH_3_OH/CH_2_Cl_2_, 0.05:10). mp 161 ± 1 °C. ^1^H NMR (400 MHz, CDCl_3_): δ 10.05 (s, 1H), 8.05–7.99 (m, 3H), 7.59 (d, 2H, *J* = 8.5 Hz), 7.38 (s, 1H), 7.26 (s, 1H). ^13^C NMR (101 MHz, CDCl_3_): δ 190.6, 141.7, 135.4, 135.0, 131.6, 131.2, 121.1, 117.7. HRMS (ESI): calcd for C_10_H_8_N_2_O [M + H]^+^, 173.0709; found, 173.0705.

### 3-Chloro-4-(dipropylamino)benzaldehyde (7)

Starting reagents were 3-chloro-4-fluorobenzaldehyde and dipropylamine, processed at 100 °C for 24 h. The crude was purified by column chromatography using EA/PE (0.1:10) to afford 33.1% of the title product as a yellow oil. *R*_f_ = 0.42 (EA/PE, 1:10). ^1^H NMR (400 MHz, CDCl_3_): δ 9.73 (s, 1H), 7.75 (d, 1H, *J* = 1.9 Hz), 7.58 (dd, 1H, *J* = 8.4, 1.9 Hz), 6.99 (d, 1H, *J* = 8.4 Hz), 3.21–3.05 (m, 4H), 1.58–1.38 (m, 4H), 0.80 (t, 6H, *J* = 7.4 Hz). ^13^C NMR (101 MHz, CDCl_3_): δ 189.7, 153.8, 132.9, 130.1, 128.7, 127.7, 121.2, 53.8, 20.6, 11.4. HRMS (ESI): calcd for C_13_H_18_ClNO [M + H]^+^, 240.1150; found, 240.1150.

### 3-Chloro-4-(piperidin-1-yl)benzaldehyde (8)

Starting reagents were 3-chloro-4-fluorobenzaldehyde and piperidine, processed at 100 °C for 24 h. The crude was purified by column chromatography using EA/PE (0.2:10) to afford 65.0% of the title product as a yellow oil. *R*_f_ = 0.08 EA/PE (0.2:10). ^1^H NMR (400 MHz, CDCl_3_): δ 9.84 (s, 1H), 7.86 (d, 1H, *J* = 1.9 Hz), 7.71 (dd, 1H, *J* = 8.2, 1.9 Hz), 7.11 (d, 1H, *J* = 8.2 Hz), 3.22–3.08 (m, 4H), 1.86–1.70 (m, 4H), 1.65 (m, 2H). ^13^C NMR (101 MHz, CDCl_3_): δ 189.9, 155.6, 132.1, 130.9, 129.6, 128.3, 120.0, 52.2, 25.9, 24.1. HRMS (ESI): calcd for C_12_H_14_ClNO [M + H]^+^, 224.0837; found, 224.0832.

### 3-Chloro-4-morpholinobenzaldehyde (9)

Starting reagents were 3-chloro-4-fluorobenzaldehyde and morpholine, at processed 100 °C for 24 h. The crude was purified by column chromatography using EA/PE (0.5:10) to afford 87% of the title product as a yellow solid. *R*_f_ = 0.24 (EA/PE, 2:10). mp 83 ± 1 °C. ^1^H NMR (400 MHz, CDCl_3_): δ 9.88 (s, 1H), 7.89 (d, 1H, *J* = 1.9 Hz), 7.76 (dd, 1H, *J* = 8.3, 1.9 Hz), 7.12 (d, 1H, *J* = 8.3 Hz), 3.98–3.80 (m, 4H), 3.27–3.12 (m, 4H). ^13^C NMR (101 MHz, CDCl_3_): δ 189.9, 154.2, 132.1, 131.7, 129.7, 128.5, 119.9, 66.8, 51.1. HRMS (ESI): calcd for C_11_H_12_ClNO_2_ [M + H]^+^, 226.0629; found, 226.0630.

### 3-Chloro-4-(4-methylpiperazin-1-yl)benzaldehyde (10)

Starting reagents were 3-chloro-4-fluorobenzaldehyde and *N*-methylpiperazine, processed at 100 °C for 24 h. The crude was purified by column chromatography starting with CH_2_Cl_2_ and gradually increasing CH_3_OH to final mixture of CH_3_OH/CH_2_Cl_2_ (0.5:10), affording 80.5% of the title product as a yellow solid. *R*_f_ = 0.20 (CH_3_OH/CH_2_Cl_2_, 0.5:10). mp 42 ± 1 °C. ^1^H NMR (400 MHz, CDCl_3_): δ 9.76 (s, 1H), 7.77 (d, 1H, *J* = 1.9 Hz), 7.63 (dd, 1H, *J* = 8.3, 1.9 Hz), 7.02 (d, 1H, *J* = 8.3 Hz), 3.15 (m, 4H), 2.55 (m, 4H), 2.29 (s, 3H). ^13^C NMR (101 MHz, CDCl_3_): δ 189.8, 154.5, 132.0, 131.4, 129.7, 128.3, 120.0, 54.9, 50.6, 46.0. HRMS (ESI): calcd for C_12_H_15_ClN_2_O [M + H]^+^, 239.0944; found, 239.0946.

### 3-Chloro-4-(diethylamino)benzaldehyde (11)

Starting reagents were 3-chloro-4-fluorobenzaldehyde and diethylamine, processed at 55 °C for 48 h. The crude was purified by column chromatography using EA/PE (0.1:10) to afford 65.9% of the title product as a yellow oil. *R*_f_ = 0.60 (EA/PE, 2:10). ^1^H NMR (400 MHz, CDCl_3_): δ 9.73 (s, 1H), 7.76 (d, 1H, *J* = 1.9 Hz), 7.59 (dd, 1H, *J* = 8.5, 1.9 Hz), 6.99 (d, 1H, *J* = 8.5 Hz), 3.24 (q, 4H, *J* = 7.1 Hz), 1.05 (t, 6H, *J* = 7.1 Hz). ^13^C NMR (101 MHz, CDCl_3_): δ 189.8, 153.5, 132.7, 130.2, 128.8, 127.8, 121.0, 45.7, 12.5. HRMS (ESI): calcd for C_11_H_14_ClNO [M + H]^+^, 212.0837; found, 212.0835.

### 3-Chloro-4-(pyrrolidin-1-yl)benzaldehyde (12)

Starting reagents were 3-chloro-4-fluorobenzaldehyde and pyrrolidine, processed at 60 °C for 24 h. The crude was purified by column chromatography using EA/PE (0.5:10) to yield 81.9% of the title product as a yellow oil. *R*_f_ = 0.54 (EA/PE, 2:10). ^1^H NMR (400 MHz, CDCl_3_): δ 9.61 (s, 1H), 7.66 (d, 1H, *J* = 1.9 Hz), 7.50 (dd, 1H, *J* = 8.6, 1.9 Hz), 6.66 (d, 1H, *J* = 8.6 Hz), 3.52 (t, 4H, *J* = 6.6 Hz), 1.97–1.77 (m, 4H). ^13^C NMR (101 MHz, CDCl_3_): δ 189.3, 150.7, 133.9, 129.5, 127.1, 119.5, 115.3, 51.2, 25.8. HRMS (ESI): calcd for C_11_H_12_ClNO [M + H]^+^, 210.0680; found, 210.0678.

### 3-Bromo-4-(diethylamino)benzaldehyde (13)

Starting reagents were 3-bromo-4-fluorobenzaldehyde and diethylamine, processed at 55 °C for 48 h. The crude was purified by column chromatography using EA/PE (0.1:10) to yield 72.6% of the title product as a yellow oil. *R*_f_ = 0.43 (EA/PE, 1:10). ^1^H NMR (400 MHz, CDCl_3_): δ 9.74 (s, 1H), 7.98 (d, 1H, *J* = 2.0 Hz), 7.65 (dd, 1H, *J* = 8.3, 2.0 Hz), 7.02 (d, 1H, *J* = 8.3 Hz), 3.20 (q, 1H, *J* = 7.1 Hz), 1.03 (t, 1H, *J* = 7.1 Hz). ^13^C NMR (101 MHz, CDCl_3_): δ 189.8, 155.1, 136.0, 131.2, 129.2, 122.2, 119.2, 46.0, 12.4. HRMS (ESI): calcd for C_11_H_14_BrNO [M + H]^+^, 256.0332; found, 256.0333, calculated for C_11_H_14_BrNO [M + H_+2_]^+^, 258.0311; found, 258.0309.

### 3-Bromo-4-(dipropylamino)benzaldehyde (14)

Starting reagents were 3-bromo-4-fluorobenzaldehyde and dipropylamine, processed at 80 °C for 24 h. The crude was purified by column chromatography using EA/PE (0.05:10) to yield 48.5% of the title product as a yellow oil. *R*_f_ = 0.45 (EA/PE, 1:10). ^1^H NMR (400 MHz, CDCl_3_): δ 9.73 (s, 1H), 7.97 (d, 1H, *J* = 1.9 Hz), 7.64 (dd, 1H, *J* = 8.3, 1.9 Hz), 7.02 (d, 1H, *J* = 8.3 Hz), 3.17–3.04 (m, 4H), 1.54–1.38 (m, 4H), 0.79 (t, 6H, *J* = 7.4 Hz). ^13^C NMR (101 MHz, CDCl_3_): δ 189.8, 155.4, 136.2, 131.0, 129.2, 122.2, 118.8, 54.0, 20.4, 11.5. HRMS (ESI): calcd for C_13_H_18_BrNO [M + H]^+^, 284.0645; found, 284.0645, calculated for C_13_H_18_BrNO [M + H_+2_]^+^, 286.0624; found, 286.0620.

### 3-Methyl-4-(piperidin-1-yl)benzaldehyde (15)

Starting reagents were 4-fluoro-3-methylbenzaldehyde and piperidine, processed at 100 °C for 24 h. The crude was purified by column chromatography using EA/PE (0.1:10) to yield 64.7% of the title product as a yellow oil. *R*_f_ = 0.46 (EA/PE, 1:10). ^1^H NMR (400 MHz, CDCl_3_): δ 9.77 (s, 1H), 7.60 (s, 1H) 7.58–7.53 (m, 1H), 6.94 (d, 1H, *J* = 8.1 Hz), 2.95–2.78 (m, 4H), 2.25 (s, 3H), 1.71–1.59 (m, 4H), 1.53 (dt, 2H, *J* = 10.9, 5.6 Hz). ^13^C NMR (101 MHz, CDCl_3_): δ 191.5, 158.6, 132.5, 132.0, 130.4, 129.3, 118.5, 52.5, 26.3, 24.3, 18.6. HRMS (ESI): calcd for C_13_H_17_NO [M + H]^+^, 204.1383; found, 204.1381.

### 4-Isopropoxybenzaldehyde (16)

Starting reagents were 4-hydroxybenzaldehyde and 2-iodopropane. The crude was purified by column chromatography using EA/PE (0.1:10) to yield quantitatively the title compound as a pale-yellow oil. *R*_f_ = 0.38 (EA/PE, 1:10). ^1^H NMR (400 MHz, CDCl_3_): δ 9.87 (s, 1H), 7.95–7.68 (m, 2H), 7.09–6.84 (m, 2H), 4.68 (Sep, 1H, *J* = 6.1 Hz), 1.38 (d, 6H, *J* = 6.1 Hz). ^13^C NMR (101 MHz, CDCl_3_): δ 190.8, 163.2, 132.1, 129.5, 115.6, 70.3, 21.9. HRMS (ESI): calcd for C_10_H_12_O_2_ [M + H]^+^, 165.0910; found, 165.0906.

### 4-(Dipropylamino)-3-methoxybenzaldehyde (17)

Starting reagents were 4-fluoro-3-methoxybenzaldehyde and dipropylamine, processed at 100 °C for 5 days. The crude was purified by column chromatography using EA/PE (0.1:10) to yield 65.9% of the title compound as a yellow oil. *R*_f_ = 0.12 (EA/PE, 0.5:10). ^1^H NMR (400 MHz, CDCl_3_): δ 9.68 (s, 1H), 7.28 (dd, 1H, *J* = 8.7, 1.7 Hz), 7.26 (d, 1H, *J* = 1.7 Hz), 6.75 (d, 1H, *J* = 8.7 Hz), 3.80 (s, 3H), 3.25–3.09 (m, 4H), 1.59–1.40 (m, 4H), 0.80 (t, 6H, *J* = 7.4 Hz). ^13^C NMR (101 MHz, CDCl_3_): δ 190.5, 151.4, 146.4, 128.5, 126.7, 116.9, 109.9, 55.6, 54.2, 20.8, 11.5. HRMS (ESI): calcd for C_14_H_21_NO_2_ [M + H]^+^, 236.1645; found, 236.1640.

### 4-(Dipropylamino)-3-Nitrobenzaldehyde (18)

Starting reagents were 4-fluoro-3-nitrobenzaldehyde and dipropylamine, processed at 25 °C for 1 h. The crude was purified by column chromatography using CH_2_Cl_2_ to yield 96% of the title product as a dark yellow solid. *R*_f_ = 0.44 (EA/PE, 2:10). mp 42 ± 0.5 °C. ^1^H NMR (400 MHz, CDCl_3_): δ 9.78 (s, 1H), 8.18 (d, 1H, *J* = 2.0 Hz), 7.84 (dd, 1H, *J* = 8.9, 2.0 Hz), 7.12 (d, 1H, *J* = 8.9 Hz), 3.28–3.12 (m, 4H), 1.71–1.52 (m, 4H), 0.87 (t, 6H, *J* = 7.4 Hz). ^13^C NMR (101 MHz, CDCl_3_): δ 188.7, 148.9, 139.0, 132.2, 130.4, 125.8, 119.7, 53.7, 20.7, 11.2. HRMS (ESI): calcd for C_13_H_18_N_2_O_3_ [M + H]^+^, 251.1390; found, 251.1388.

### 4-(Diethylamino)-3-Nitrobenzaldehyde (19)

Starting reagents were 4-fluoro-3-nitrobenzaldehyde and dirthylamine, processed at 25 °C for 1 h. The crude was purified by column chromatography using CH_2_Cl_2_ to yield 91.7% of the title product as a dark yellow solid. *R*_f_ = 0.29 (EA/PE, 2:10). mp 50 ± 0.5 °C. ^1^H NMR (400 MHz, CDCl_3_): δ 9.77 (s, 1H), 8.14 (d, 1H, *J* = 2.0 Hz), 7.83 (dd, 1H, *J* = 8.9, 2.0 Hz), 7.10 (d, 1H, *J* = 8.9 Hz), 3.32 (q, 4H, *J* = 7.1 Hz), 1.20 (t, 6H, *J* = 7.1 Hz). ^13^C NMR (101 MHz, CDCl_3_): δ 188.7, 148.1, 138.9, 132.3, 130.2, 125.8, 119.2, 46.0, 12.4. HRMS (ESI): calcd for C_11_H_14_N_2_O_3_ [M + H]^+^, 223.1077; found, 223.1074.

### 4-(Diethylamino)-3-methoxybenzaldehyde (20)

Starting reagents were 4-fluoro-3-methoxybenzaldehyde and diethylamine, processed at 100 °C for 5 days. The crude compound was purified by column chromatography starting with EA/PE (0.1:10) and gradually increasing to EA/PE (0.5:10) to yield 60.4% of the title compound as a yellow oil. *R*_f_ = 0.26 (EA/PE, 1:10). ^1^H NMR (400 MHz, CDCl_3_): δ 9.70 (s, 1H), 7.30 (dd, 1H, *J* = 7.9, 1.5 Hz), 7.27 (d, 1H, *J* = 1.5 Hz), 6.80 (d, 1H, *J* = 7.9 Hz), 3.81 (s, 3H), 3.26 (q, 4H, *J* = 7.0 Hz), 1.05 (t, 6H, *J* = 7.1 Hz). ^13^C NMR (101 MHz, CDCl_3_): δ 190.6, 151.7, 146.1, 128.9, 126.6, 117.3, 109.6, 55.6, 45.7, 12.7. HRMS (ESI): calcd for C_12_H_17_NO_2_ [M + H]^+^, 208.1332; found, 208.1330.

### 3-Bromo-4-(4-methylpiperazin-1-yl)benzaldehyde (21)

Starting reagents were 3-bromo-4-fluorobenzaldehyde and *N*-methylpiperazine, processed at 100 °C for 24 h. The crude was purified by column chromatography starting with CH_2_Cl_2_ and followed by a gradual increase of polarity to (CH_3_OH/CH_2_Cl_2_, 0.5:10) to yield 93.0% of the title product as a yellow oil, which was solidified under vacuum. *R*_f_ = 0.13 (CH_3_OH/CHCl2, 0.5:10). mp 55 ± 1 °C. ^1^H NMR (400 MHz, CDCl_3_): δ 9.74 (s, 1H), 7.95 (d, 1H, *J* = 1.9 Hz), 7.67 (dd, 1H, *J* = 8.3, 1.9 Hz), 7.02 (d, 1H, *J* = 8.3 Hz), 3.21–3.10 (m, 4H), 2.61–2.49 (m, 4H), 2.29 (s, 3H). ^13^C NMR (101 MHz, CDCl_3_): δ 189.6, 155.8, 135.3, 131.9, 130.2, 120.4, 118.7, 54.8, 51.0, 46.0. HRMS (ESI): calcd for C_12_H_15_BrN_2_O [M + H]^+^, 283.0441; found, 283.0443. [M_+2_ + H]^+^, 285.0420; found, 285.0419.

### 3-Bromo-4-morpholinobenzaldehyde (22)

Starting reagents were 3-bromo-4-fluorobenzaldehyde and morpholine, processed at 100 °C for 48 h. The crude was purified by column chromatography using CH_2_Cl_2_ to yield 71.5% of the title product as a yellow solid. *R*_f_ = 0.20 (Ch3OH/CH_2_Cl_2_, 0.5:10). mp 96 ± 1 °C. ^1^H NMR (400 MHz, CDCl_3_): δ 9.78 (s, 1H), 8.00 (d, 1H, *J* = 1.8 Hz), 7.71 (dd, 1H, *J* = 8.2, 1.9 Hz), 7.03 (d, 1H, *J* = 8.3 Hz), 3.93–3.71 (m, 4H), 3.18–3.02 (m, 4H). ^13^C NMR (101 MHz, CDCl_3_): δ 189.8, 155.6, 135.5, 132.3, 130.2, 120.4, 118.9, 66.8, 51.5. HRMS (ESI): calcd for C_11_H_12_BrNO_2_ [M + H]^+^, 270.0124; found, 270.0126. [M_+2_ + H]^+^, 272.0104; found, 272.0103.

### 3-Bromo-4-(piperidin-1-yl)benzaldehyde (23)

Starting reagents were 3-bromo-4-fluorobenzaldehyde and piperidine, processed at 100 °C for 24 h. The crude was purified by column chromatography using EA/PE (0.1:10) to yield 84.6% of the title product as a yellow oil. *R*_f_ = 0.33 (EA/PE, 1:10). ^1^H NMR (400 MHz, CDCl_3_): δ 9.81 (s, 1H), 8.03 (d, 1H, *J* = 1.9 Hz), 7.73 (dd, 1H, *J* = 8.3, 1.9 Hz), 7.07 (d, 1H, *J* = 8.3 Hz), 3.18–2.99 (m, 4H), 1.85–1.68 (m, 4H), 1.62 (m, 2H). ^13^C NMR (101 MHz, CDCl_3_): δ 189.8, 157.1, 135.4, 131.5, 130.1, 120.4, 118.9, 52.7, 25.9, 24.0. HRMS (ESI): calcd for C_12_H_14_BrNO [M + H]^+^, 268.0332; found, 268.0333. [M_+2_ + H]^+^, 270.0311; found, 270.0310.

### 3-Methyl-4-(4-methylpiperazin-1-yl)benzaldehyde (24)

Starting reagents were 4-fluoro-3-methylbenzaldehyde and *N*-methylpiperazine, processed at 100 °C for 6 days. The crude was purified by column chromatography starting with CH_2_Cl_2_ and gradually increasing polarity to CH_3_OH/CH_2_Cl_2_ (0.5:10) to yield 71.4% of the title product as a pale yellow solid. *R*_f_ = 0.420 (CH_3_OH/CH_2_Cl_2_, 1:10). mp 68 ± 0.5 °C. ^1^H NMR (400 MHz, CDCl_3_): δ 9.82 (s, 1H), 7.63 (s, 1H), 7.60 (m, 1H), 7.02 (d, 1H, *J* = 8.0 Hz), 2.98 (t, 4H, *J* = 4.78 Hz), 2.56 (s, 4H), 2.33 (s, 3H), 2.30 (s, 3H). ^13^C NMR (101 MHz, CDCl_3_): δ 191.32, 157.19, 132.43, 131.97, 130.90, 129.31, 118.54, 55.21, 50.92, 46.06, 18.52. HRMS (ESI): calcd for C_13_H_18_N_2_O [M + H]^+^, 219.1495; found, 219.1497.

### 4-(4-Methylpiperazin-1-yl)-3-nitrobenzaldehyde (25)

Starting reagents were 4-fluoro-3-nitrobenzaldehyde and *N*-methylpiperazine, processed at 25 °C for 1 h. The crude was purified by column chromatography using CH_2_Cl_2_ followed by (CH_3_OH/CH_2_Cl_2_, 0.1:10) to yield quantitatively the title compound as a yellow solid. *R*_f_ = 0.24 (CH_3_OH/CH_2_Cl_2_, 0.5:10). mp 101 ± 1 °C. ^1^H NMR (400 MHz, CDCl_3_): δ 9.82 (s, 1H), 8.23 (d, 1H, *J* = 2.0 Hz), 7.90 (dd, 1H, *J* = 8.7, 2.0 Hz), 7.13 (d, 1H, *J* = 8.7 Hz), 3.32–3.16 (m, 4H), 2.64–2.47 (m, 4H), 2.34 (s, 3H). ^13^C NMR (101 MHz, CDCl_3_): δ 188.7, 149.4, 139.8, 133.2, 129.7, 127.7, 119.9, 54.4, 50.5, 45.9. HRMS (ESI): calcd for C_12_H_15_N_3_O_3_ [M + H]^+^, 250.1191; found, 250.1192.

### 3-Methyl-4-(pyrrolidin-1-yl)benzaldehyde (26)

Starting reagents were 4-fluoro-3-methylbenzaldehyde and pyrrolidine, processed at 100 °C for 48 h. The crude was purified by column chromatography using first EA/PE (0.1:10) before increasing to EA/PE (0.2:10) to yield 70.4% of the title compound as a dark yellow oil. *R*_f_ = 0.10 (EA/PE, 0.5:10). ^1^H NMR (400 MHz, CDCl_3_): δ 9.64 (s, 1H), 7.51–7.48 (m, 1H), 7.48–7.45 (m, 1H), 6.64 (d, 1H, *J* = 9.0 Hz), 3.44–3.28 (m, 4H), 2.34 (s, 3H), 1.94–1.80 (m, 4H). ^13^C NMR (101 MHz, CDCl_3_): δ 191.5, 154.4, 134.1, 129.9 127.1, 124.7, 113.8, 51.0, 25.7, 22.0. HRMS (ESI): calcd for C_12_H_15_NO [M + H]^+^, 190.1226; found, 190.1221.

### 3-Methyl-4-morpholinobenzaldehyde (27)

Starting reagents were 4-fluoro-3-methylbenzaldehyde and morpholine, at 100 °C for 6 days. The crude was purified by column chromatography using first EA/PE (0.5:10) followed by EA/PE (1:10) to yield 45.9% of the title product as a yellow solid. *R*_f_ = 0.08 (EA/PE, 1:10). mp 67 ± 1 °C. ^1^H NMR (400 MHz, CDCl_3_): δ 9.81 (s, 1H), 7.62–7.59 (m, 1H), 7.58–7.62 (m, 1H), 7.00 (d, 1H, *J* = 7.9 Hz), 3.91–3.64 (m, 4H), 3.02–2.82 (m, 4H), 2.29 (s, 3H). ^13^C NMR (101 MHz, CDCl_3_): δ 191.4, 156.9, 132.6, 132.2, 131.3, 129.4, 118.5, 67.1, 51.6, 18.5. HRMS (ESI): calcd for C_12_H_15_NO_2_ [M + H]^+^, 206.1176; found, 206.1173.

### 3-Methoxy-4-(piperidin-1-yl)benzaldehyde (28)

Starting reagents were 4-fluoro-3-methoxybenzaldehyde and piperidine, processed at 100 °C for 48 h. The crude was purified by column chromatography using first EA/PE (0.1:10) followed with gradual increase up to EA/PE (2:10) to yield 70.4% of the title compound as a yellow oil. *R*_f_ = 0.50 (EA/PE, 2:10). ^1^H NMR (400 MHz, CDCl_3_): δ 9.75 (s, 1H), 7.32 (dd, 1H, *J* = 8.1, 1.8 Hz), 7.28 (d, 1H, *J* = 1.8 Hz), 6.89 (d, 1H, *J* = 8.1 Hz), 3.84 (s, 3H), 3.11–2.96 (m, 4H), 1.74–1.60 (m, 4H), 1.53 (m, 2H). ^13^C NMR (101 MHz, CDCl_3_): δ 191.0, 152.2, 148.6, 130.4, 126.6, 117.4, 109.1, 55.6, 51.5, 26.0, 24.3. HRMS (ESI): calcd for C_13_H_17_NO_2_ [M + H]^+^, 220.1332; found, 220.1330.

### 3-Methoxy-4-(pyrrolidin-1-yl)benzaldehyde (29)

Starting reagents were 4-fluoro-3-methoxybenzaldehyde and pyrrolidine, processed at 100 °C for 48 h. The crude was purified by column chromatography using first EA/PE (0.1:10) and then EA/PE (0.5:10) to yield 57.9% of the title compound as a yellow solid. *R*_f_ = 0.25 (EA/PE, 0.1:10). mp 38 ± 0.5 °C. ^1^H NMR (400 MHz, CDCl_3_): δ 9.60 (s, 1H), 7.23 (dd, 1H, *J* = 8.0, 1.7 Hz), 7.20 (d, 1H, *J* = 1.8 Hz), 6.50 (d, 1H, *J* = 8.0 Hz), 3.74 (s, 3H), 3.44 (t, 4H, *J* = 6.7 Hz), 1.84 (t, 4H, *J* = 6.7 Hz). ^13^C NMR (101 MHz, CDCl_3_): δ 190.0, 148.7, 145.1, 128.1, 126.4, 112.6, 109.7, 55.7, 50.6, 25.5. HRMS (ESI): calcd for C_12_H_15_NO_2_ [M + H]^+^, 206.1176; found, 206.1174.

### 3-Methoxy-4-(4-methylpiperazin-1-yl)benzaldehyde (30)

Starting reagents were 4-fluoro-3-methoxybenzaldehyde and *N*-methylpiperazine, processed at 100 °C for 48 h. The crude was purified by column chromatography using first CH_3_OH/CH_2_Cl_2_ (0.1:10) with gradual increase of CH_3_OH to CH_3_OH/CH_2_Cl_2_ (0.5:10) to yield 66.9% of the title product as a yellow solid. *R*_f_ = 0.28 (CH_3_OH/CH_2_Cl_2_, 0.5:10). mp 78 ± 1 °C. ^1^H NMR (400 MHz, Acetone): δ 9.85 (s, 1H), 7.47 (dd, 1H, *J* = 8.1, 1.7 Hz), 7.40 (d, 1H, *J* = 1.7 Hz), 7.03 (d, 1H, *J* = 8.1 Hz), 3.93 (s, 3H), 3.21 (t, 4H, *J* = 4.7 Hz), 2.51 (t, 4H, *J* = 4.7 Hz), 2.27 (s, 3H). ^13^C NMR (101 MHz, acetone): δ 191.2, 153.1, 148.3, 131.7, 126.5, 118.1, 110.8, 56.0, 55.9, 50.6, 46.4. HRMS (ESI): calcd for C_13_H_18_N_2_O_2_ [M + H]^+^, 235.1441; found, 235.1439.

### 3-Methoxy-4-morpholinobenzaldehyde (31)

Starting reagents were 4-fluoro-3-methoxybenzaldehyde and morpholine, processed at 100 °C for 48 h. The crude was purified by column chromatography using first EA/PE (0.5:10) then gradually increase of EA to EA/PE (2:10) to yield 68.4% of the title compound as a pale-yellow solid. *R*_f_ = 0.59 (EA/PE, 1:1). mp 96 ± 1 °C. ^1^H NMR (400 MHz, acetone): δ 9.72 (s, 1H), 7.35 (dd, 1H, *J* = 8.1, 1.8 Hz), 7.27 (d, 1H, *J* = 1.8 Hz), 6.90 (d, 1H, *J* = 8.1 Hz), 3.79 (s, 3H), 3.62 (t, 4H, *J* = 4.6 Hz), 3.13 (t, 4H, *J* = 4.6 Hz). ^13^C NMR (101 MHz, acetone): δ 191.3, 153.1, 148.0, 132.0, 126.4, 118.0, 110.8, 67.4, 56.0, 51.2. HRMS (ESI): calcd for C_12_H_15_NO_3_ [M + H]^+^, 222.1125; found, 222.1123.

### 4-Morpholino-3-Nitrobenzaldehyde (32)

Starting reagents were 4-fluoro-3-nitrobenzaldehyde and morpholine, processed at 25 °C for 1 h. The crude was purified by column chromatography using CH_2_Cl_2_ and gradual increase to CH_3_OH/CH_2_Cl_2_ (0.1:10) to yield quantitatively the title compound as a dark yellow oil. *R*_f_ = 0.35 (EA/PE, 4:10). ^1^H NMR (400 MHz, Acetone): δ 9.93 (s, 1H), 8.31 (d, 1H, *J* = 1.9 Hz), 8.03 (dd, 1H, *J* = 8.7, 1.9 Hz), 7.42 (d, 1H, *J* = 8.7 Hz), 3.80 (t, 4H, *J* = 4.7 Hz), 3.26 (t, 4H, *J* = 4.7 Hz). ^13^C NMR (101 MHz, CDCl_3_): δ 189.9, 150.0, 141.1, 134.0, 129.7, 129.2, 121.1, 66.9, 51.6. HRMS (ESI): calcd for C_11_H_12_N_2_O_4_ [M + H]^+^, 237.0875; found, 237.0879.

### 4-Isopropoxy-3-methoxybenzaldehyde (33)

Starting reagents were 4-hydroxy-3-methoxybenzaldehyde and 2-iodopropane. The crude was purified by column chromatography using CH_2_Cl_2_ to yield quantitatively the title compound as a yellow oil. *R*_f_ = 0.18 (CH_2_Cl_2_). ^1^H NMR (400 MHz, Acetone): δ 9.86 (s, 1H), 7.50 (dd, 1H, *J* = 8.2, 1.9 Hz), 7.43 (d, 1H, *J* = 1.9 Hz), 7.12 (d, 1H, *J* = 8.2 Hz), 4.75 (sept, 1H, *J* = 6.1 Hz), 3.89 (s, 3H), 1.36 (d, 6H, *J* = 6.1 Hz). ^13^C NMR (101 MHz, Acetone): δ 191.2, 154.0, 151.5, 131.1, 126.6, 114.4, 111.0, 71.7, 56.1, 22.2. HRMS (ESI): calcd for C_11_H_14_O_3_ [M + H]^+^, 195.1016; found, 195.1013.

### 3-Nitro-4-(pyrrolidin-1-yl)benzaldehyde (34)

Starting reagents were 4-fluoro-3-nitrobenzaldehyde and pyrrolidine, processed at 25 °C for 1 h. The crude was purified by column chromatography using CH_2_Cl_2_ to yield quantitatively the title compound as a yellow solid. *R*_f_ = 0.20 (EA/PE, 2:10). mp 118 ± 1 °C. ^1^H NMR (400 MHz, Acetone): δ 9.81 (s, 1H), 8.19 (d, 1H, *J* = 2.0 Hz), 7.87 (dd, 1H, *J* = 8.9, 2.0 Hz), 7.13 (d, 1H, *J* = 8.9 Hz), 3.33 (t, 4H, *J* = 6.5 Hz), 2.05 (t, 4H, *J* = 6.5 Hz). ^13^C NMR (101 MHz, acetone): δ 189.3, 146.5, 137.3, 132.6, 130.7, 125.4, 117.5, 51.5, 26.2. HRMS (ESI): calcd for C_11_H_12_N_2_O_3_ [M + H]^+^, 221.0926; found, 221.0932.

### 4-Isopropoxy-3-methoxy-5-Nitrobenzaldehyde (35)

Starting reagents were 4-hydroxy-3-methoxy-5-nitrobenzaldehyde and 2-iodopropane. The crude was purified by column chromatography using EA/PE (0.5:10) with a gradual increase to EA/PE (3:10) to yield 25.8% of the title compound as a pale yellow solid. *R*_f_ = 0.41 (EA/PE, 3:10). mp 71 ± 0.5 °C. ^1^H NMR (400 MHz, Acetone): δ 9.85 (s, 1H), 7.76 (d, 1H, *J* = 1.8 Hz), 7.63 (d, 1H, *J* = 1.8 Hz), 4.88–4.76 (sept, 1H, *J* = 6.1 Hz), 3.93 (s, 3H), 1.15 (d, 6H, *J* = 6.1 Hz). ^13^C NMR (101 MHz, Acetone): δ 190.4, 155.3, 146.9, 145.5, 132.6, 118.7, 114.8, 78.1, 57.2, 22.6. HRMS (ESI): calcd for C_11_H_13_NO_5_ [M + H]^+^, 240.0872; found, 240.0869.

### 3-Nitro-4-(piperidin-1-yl)benzaldehyde (36)

Starting reagents were 4-fluoro-3-nitrobenzaldehyde and piperidine, processed at 25 °C for 1 h. The crude was purified by column chromatography using CH_2_Cl_2_ to yield quantitatively the title compound as a yellow oil. *R*_f_ = 0.31 (EA/PE, 2:10). ^1^H NMR (400 MHz, acetone): δ 9.75 (s, 1H), 8.12 (d, 1H, *J* = 2.0 Hz), 7.82 (dd, 1H, *J* = 8.7, 2.0 Hz), 7.23 (d, 1H, *J* = 8.7 Hz), 3.14–3.07 (m, 4H), 1.67–1.44 (m, 6H). ^13^C NMR (101 MHz, acetone): δ 189.7, 150.6, 140.7, 133.6, 130.0, 128.1, 121.1, 52.4, 26.4, 24.4. HRMS (ESI): calcd for C_12_H_14_N_2_O_3_ [M + H]^+^, 235.1083; found, 235.1084.

### 4-(Diethylamino)-2-methoxybenzaldehyde (37)

Starting reagents were with 4-fluoro-2-methoxybenzaldehyde and diethylamine, processed at 100 °C for 48 h. The crude was purified by column chromatography using EA/PE (1:10) to yield 59.4% of the title compound as yellow solid. *R*_f_ = 0.20 (EA/PE, 2:10). mp 101 ± 1 °C. ^1^H NMR (400 MHz, Acetone): δ 9.98 (s, 1H), 7.45 (d, 1H, *J* = 8.9 Hz), 6.23 (dd, 1H, *J* = 8.9, 2.1 Hz), 6.10 (d, 1H, *J* = 2.1 Hz), 3.78 (s, 3H), 3.37 (q, 4H, *J* = 7.1 Hz), 1.07 (t, 6H, *J* = 7.1 Hz). ^13^C NMR (101 MHz, acetone) δ 186.0, 165.0, 154.8, 130.3, 115.0, 105.1, 93.6, 55.7, 45.2, 12.8. HRMS (ESI): calcd for C_12_H_17_NO_2_ [M + H]^+^, 208.1332; found, 208.1329.

### 4-(Dipropylamino)-2-methoxybenzaldehyde (38)

Starting reagents were 4-fluoro-2-methoxybenzaldehyde and dipropylamine, processed at 100 °C for 48 h. The crude was purified by column chromatography using EA/PE (1:10) to yield 43.7% of the title compound as yellow solid. *R*_f_ = 0.38 (EA/PE, 2:10). mp 92 ± 1 °C. ^1^H NMR (400 MHz, acetone) δ 9.97 (s, 1H), 7.44 (d, 1H, *J* = 8.9 Hz), 6.22 (dd, 1H, *J* = 8.9, 2.1 Hz), 6.08 (d, 1H, *J* = 2.1 Hz), 3.77 (s, 3H), 3.28 (t, 4H, *J* = 7.6 Hz), 1.62–1.43 (sextet, 4H, *J* = 7.5 Hz), 0.81 (t, 6H, *J* = 7.4 Hz). ^13^C NMR (101 MHz, acetone) δ 186.0, 164.9, 155.3, 130.3, 115.0, 105.3, 93.8, 55.7, 53.2, 21.2, 11.5. HRMS (ESI): calcd for C_14_H_21_NO_2_ [M + H]^+^, 236.1645; found, 236.1643.

### *tert*-Butyl 4-(4-formyl-2-nitrophenyl)piperazine-1-carboxylate (39)

To a solution of 4-fluoro-3-nitrobenzaldehyde (1 equiv) in acetone (20 mL), pyridine (1 mL) and 1-Boc-piperazine (1.2 equiv) were added and stirred at room temperature for 4 h. The solvent was then evaporated under vacuum to yield a dark-yellow oil, which was purified by column chromatography using EA/PE (1:10) to afford 39.4% of the title compound as a yellow solid. *R*_f_ = 0.19 (EA/PE, 2:10). mp 141 ± 1 °C. ^1^H NMR (400 MHz, acetone): δ 9.94 (s, 1H), 8.33 (d, 1H, J = 2.0 Hz), 8.03 (dd, 1H, J = 8.9, 2.0 Hz), 7.44 (d, 1H, J = 8.9 Hz), 3.76–3.43 (m, 4H), 3.43–3.15 (m, 4H), 1.48 (s, 9H). 13C NMR (101 MHz, acetone): δ 189.91, 155.04, 150.11, 141.03, 133.88, 129.70, 129.18, 121.35, 80.08, 51.03, 44.23, 28.52. HRMS (ESI): calcd for C_16_H_21_N_3_O_5_ [M + H]^+^, 336.1559; found, 336.1560.

### 3-Nitro-4-(piperazin-1-yl)benzaldehyde (40)

To a solution of **39** (1 equiv) in CH_2_Cl_2_ (15 mL), trifluoroacetic acid (5 mL) was added and stirred at room temperature for 2 h. CH_2_Cl_2_ was then evaporated under vacuum to give a dark-yellow oil, which was purified by column chromatography using (CH_3_OH/CH_2_Cl_2_, 0.5:10) to yield quantitatively the title compound as a yellow-orange solid (82%). *R*_f_ = 0.38 (CH_3_OH/CH_2_Cl_2_, 1:10). mp 144 ± 1 °C. ^1^H NMR (400 MHz, acetone) δ 9.98 (s, 1H), 8.38 (d, 1H, J = 1.9 Hz), 8.10 (dd, 1H, J = 8.6, 1.9 Hz), 7.56 (d, 1H, J = 8.6 Hz), 3.71–3.61 (m, 4H), 3.61–3.50 (m, 4H), 2.10 (s, 1H). 13C NMR (101 MHz, acetone) δ 190.19, 149.65, 141.82, 134.47, 130.55, 129.34, 122.25, 48.60, 43.97. HRMS (ESI): calcd for C_11_H_13_N_3_O_3_ [M + H]^+^, 236.1030; found, 236.1031. Compound **40** was obtained in 82% purity.

### Purification of Recombinant Human ALDHs and Enzymatic Assays

Human ALDH1A1, ALDH1A3, and ALDH3A1 were cloned and recombinantly expressed from the pET-30 Xa/LIC vector. Protein purification was achieved by affinity chromatography on a nickel-charged chelating Sepharose Fast Flow 5-mL column (His Trap column, Cytiva), which specifically binds the protein due to its N-terminal (His)_6_ tag, using an ÄKTA FPLC system (Cytiva), as previously described.^55^ Enzymes including the His tag were stored at −80 °C in 20 mM Tris/HCl and 0.5 M NaCl, pH 8.0, until use. Reaction buffers were as follows: ALDH1A1 was assayed in 50 mM HEPES, 0.5 mM EDTA, and 0.5 mM DTT, pH 8.0; ALDH1A3 was assayed in 50 mM HEPES, 30 mM MgCl_2_, and 5 mM DTT, pH 8.0; and ALDH3A1 was assayed in 50 mM Tris–HCl and 5 mM DTT, pH 8.0. Activity under standard conditions was measured fluorimetrically at 25 °C using a Cary Eclipse (Varian) fluorimeter to follow the purification procedure and to check the enzyme concentration before each kinetic experiment. The fluorescence of NADH was characterized at 460 nm with excitation at 340 nm, and 5 μM NAD(P)H was added as an internal standard to obtain the absolute reaction rates.^55^ Standard activity was measured at saturating concentrations of substrate using 30 μM hexanal (ALDH1A1), 250 μM hexanal (ALDH1A3), or 250 μM 4-NBA (ALDH3A1). NAD^+^ was 500 μM for ALDH1A1 and ALDH1A3, and NADP^+^ was 1 mM for ALDH3A1.

### Inhibition Screening

All compounds tested were dissolved in DMSO and assayed at a final concentration of 1% (v/v) DMSO. Single-point measurements of enzymatic activity at 10 μM inhibitor were performed for the 40 DEAB analogues against the three isoforms (ALDH1A1, ALDH1A3, and ALDH3A1). For the initial screening, the enzymatic activity was measured in 96-well plates (final volume of 200 μL) in a Victor 3 Multilabel Plate Reader (Perkin Elmer), by monitoring the fluorescence of the NAD(P)H produced during the reaction (excitation at 340 nm and emission at 460 nm). Alternatively, the enzymatic activity was measured in a Varian Cary 400 UV–vis spectrophotometer, by monitoring the increase in the absorbance of NAD(P)H at 340 nm (ε = 6.22 mM^–1^·cm^–1^) or in a Cary Eclipse Varian fluorimeter, as described above. The inhibition screening was preferably performed at two substrate concentrations (near the *K*_m_ value and at a saturating substrate concentration), except for ALDH1A1, which was only tested at substrate saturation due to a lack of sensitivity when its low substrate concentration was used in the assay. For ALDH1A1, 5 μM hexanal (Sigma) was used. For ALDH1A3, 10 and 250 μM hexanal (Sigma) were used. For ALDH3A1, 4-NBA (Sigma) was used at 31 and 250 μM. All substrates were prepared in the corresponding assay buffer at a concentration of 2 mM and further diluted to reach the final concentrations required per experiment. The concentration of the enzyme was kept from 50- to 100-fold lower than that of the substrate for all enzymatic assays.

### Enzyme Kinetics with DEAB Analogues as Substrates

According to their structure with a carbonyl group, compounds were tested for their substrate properties against ALDH1A1, ALDH1A3, and ALDH3A1. For this assay, the conditions were the same as previously described except using the DEAB analogues as substrates instead of hexanal or 4-NBA at a concentration of 10 μM. Results are expressed as the percentage of activity at 10 μM versus the activity at 10 μM of their standard substrate (hexanal or 4-NBA). Values are expressed as the mean ± SE.

To calculate the *K*_m_ value of compound **18** as a substrate for ALDH3A1, several concentrations of compound **18** were used. Experimental values were fitted to the adaptation of the Michaelis–Menten equation for substrate inhibition  and shown as the mean ± SE.

### Determination of the Kinetic Constants (IC_50_ and *K*_i_)

In order to determine the IC_50_ values, reaction rates were determined at various concentrations of inhibitor at a fixed concentration of substrate. As substrates for ALDH1A1, ALDH1A3, and ALDH3A1, 5 μM hexanal, 6 μM hexanal, and 31 μM 4-NBA, respectively, were used. The IC_50_ values were calculated by nonlinear fitting of the obtained data to a sigmoidal plot using GraFit 5.0 (Erithacus software), with the following 4-parameter equation , where *y* is the specific activity, *x* is the inhibitor concentration, background is the minimum *y* value, range is the fitted uninhibited value minus the background, and *s* is the slope factor. Values are expressed as the mean ± SE.

Activity assays to determine the type of inhibition and *K*_i_ value were performed using various substrate and inhibitor concentrations maintaining the same conditions as for the IC_50_ experiments and using GraFit 5.0 for data processing. The data of enzymatic activities at different inhibitor concentrations were fitted to the Michaelis–Menten equation to determine the values of *K*_m_ and *V*_max_. Next, results were fitted to the equations for competitive, given by ; noncompetitive, given by ; uncompetitive, given by ; and mixed, given by .^56^ The type of inhibition was selected based on the lowest error value. Parameters were expressed as the mean ± SE.

Docking studies of compounds **14** and **18** on ALDH1A3 and ALDH3A1 isoforms.

(a) Protein and ligand preparation: The three X-ray complexes ALDH1A3, ALDH3A1, and ALDH1A1 were downloaded from the Protein Data Bank with their PBD IDs 5FHZ,^58^4H80,^59^ and 4WPN,^60^ respectively. The cocrystallized ligands, ions, and water molecules were removed from the X-ray complexes and H-bonds; missing residues were added to the protein with the aid of protein preparation wizard of Maestro. All the compounds were drawn using the Build panel of Maestro and subjected to a conformational search of 1000 steps in a water environment (using the generalized-Born/surface-area model) through Macromodel software. A Monte Carlo algorithm with the MMFF and a distance-dependent dielectric constant of 1.0 was applied while using Macromodel.^61^

(b) Molecular docking: All docking calculations were carried out on the X-ray structure of human ALDH1A1 in complex with selective inhibitor 1-{[1,3-dimethyl-7-(3-methylbutyl)-2,6-dioxo-2,3,6,7-tetrahydro-1*H*-purin-8-yl]methyl}piperidine-4-carboxamide (PDB ID: 4WPN), X-ray structure of human ALDH1A3 in complex with RA and NAD^+^ (PDB ID: 5FHZ), and X-ray structure of human ALDH3A1 in complex with selective inhibitor—*N*-[4-(4-methylsulfonyl-2-nitroanilino)phenyl]acetamide (PDB ID: 4H80). Glide 5.0 with the standard precision (SP) method^62^ was used for docking of all compounds on the three X-ray structures.

### GLIDE 5.0

The binding site was defined by a rectangular box of 10 Å along the *x*, *y*, and *z* axes centered on the ligand. The option of imposing a maximum value to the number of atoms that a ligand may have (when docked) was deactivated. Thus, all the ligands were docked independently from the number of their atoms, whereas the GLIDE defaults were used for the remaining parameters. The GlideScore fitness function is based on Chemscore but includes a steric-clash term and adds buried polar terms to penalize electrostatic mismatches and modifications on other secondary terms. The docking analyses were carried out using the SP method. A total of 50 docking solutions were generated for each ligand, and the top-ranked docking pose was considered as the final pose.

The reliability of the docking program: GLIDE 5.0 was assessed by performing self-docking analysis and calculating the root-mean-square deviation (RMSD) between the crystallographic position of the ligand and the ligand’s disposition predicted by docking. The rms_analysis program of the Gold suite was used to calculate the RMSD difference, considering only the heavy atoms of the ligand. The docking method is able to produce a binding pose within 2.0 Å RMSD of the crystallographic disposition, therefore considered as reliable.^63^

### Immunoblotting

The PCa cell lysates (DU145, LNCaP, and PC3) were prepared using the RIPA buffer. The lysates were briefly sonicated and centrifuged, and the protein concentrations were determined using the BCA protein assay kit (Thermo Fisher, CA, USA). A total of 40 μg of cell lysates was separated using 10% SDS-polyacrylamide gel electrophoresis and transferred on to Amersham Hybond ECL nitrocellulose membranes (Amersham, QC, Canada). The membranes were blocked using 5% skim milk in phosphate-buffered saline with 0.1% Tween-20 for 1 h and then incubated with specific primary antibodies overnight at 4 °C followed by incubation with HRP-conjugated secondary antibodies for 1 h at room temperature. Specific protein bands were visualized using an ECL detection kit (Amersham). The primary and secondary antibodies used were rabbit anti-ALDH1A1 (D9J7R, Cell signaling Technology, UK), mouse anti-GAPDH (6C_5_, Abcam, Cambridge, UK), rabbit anti-ALDH1A3 (N_2_C2, GeneTex, California, USA), mouse anti-ALDH3A1 (G-2, Santa Cruz Biotechnology, CA), anti-rabbit HRP (Dako), and goat anti-mouse HRP (Abcam, Cambridge, UK).

### Chemosensitivity Studies

MTT (3-(4, 5-dimethyl-2-thiazolyl)-2, 5-diphenyl-2*H*-tetrazolium bromide) assay was used to determine cell viability.^64^ Immortalized PCa cells (PC-3, Du-145, and LNCaP) were seeded in 96-well plates and allowed to adhere overnight at 37 °C, 5% CO_2_, and 100% humidity. The following day, cells were treated with the DEAB analogues at appropriate concentrations ranging from 12.5 to 200 μM. After 96 h of exposure, 200 μL of MTT (Sigma) solution (0.5 mg/mL) was added to each well and incubated at 37 °C for 4 h. The formazan crystals were dissolved in DMSO (Sigma), and the absorbance was read using a microplate reader (Multiskan EX; Thermo Fisher Scientific) at 540 nm. Data analysis was performed using Microsoft Excel 2013 and GraphPad Prism software.

Primary cells cultured at early passage from human prostate tissue biopsies included one BPH sample H415/15–BPH/PSA 0.61/age 74 and four PCa samples H568/15 RM–Gl7(3 + 4)/PSA 8.5/age 69, H431/14 LM–Gl7(3 + 4)/PSA 14/age 66, H488/14 RM–Gl7(3 + 4)/PSA 12.6/age 60, and H517/15 RM–Gl7(3 + 4)/PSA 4.4/age 65. Primary cells were obtained with ethical consent (REC ref 07/H1304/121) at radical prostatectomy (cancer) and transurethral resection (BPH) and were seeded as previously described^37^ in 96-well plates 5000 cells/well in 100 μL of SCM and incubated at 37 °C, 5% CO_2_ for 24 h. DEAB, **14**, and **18** were prepared in DMSO at a stock concentration of 200 mM. Cells were treated with 100 μL of ALDH inhibitors at concentrations of 50 and 200 μM as single treatment. Combination treatments included 100 μL of 50 μM ALDH inhibitor + 1 nM docetaxel and 100 μL of 200 μM ALDH inhibitor + 1 nM docetaxel. Cells were also treated with 100 μL of 1 nM docetaxel only. 1 nM docetaxel was chosen since it was the IC_50_ when other primary samples were analyzed in our laboratory. Control wells included blank (media only) and untreated cells (DMSO only). Each experiment was performed in triplicate, and cell seeding density was the same in all wells; therefore, changes in treated cells were compared to untreated DMSO control cells. Plates were returned to the incubator for a 72 h incubation before further processing. Alamar Blue solution was added at 10% of total sample volume, and plates were incubated at 37 °C for 1–4 h before absorbance was analyzed in a plate reader.^65^ Alamar Blue has an excitation wavelength of 530–560 nm and an emission wavelength of 590 nm. Total % cell viability/cell survival was calculated by dividing the absorbance of the treated sample by the absorbance of control and multiplying it by 100.

## Supporting Information Available

HPLC traces for all compounds. PDB codes 4WPN (ALDH1A1), 5FHZ (ALDH1A3) and 4H80 (ALDH3A1) were used for docking studies of compounds **14** and **18**, respectively (ZIP)
Molecular Formula Strings (CSV)
Additional data include results of inhibition screening as % of remaining ALDH activities in the presence of DEAB analogues, % of substrate activities of DEAB analogues,IC_50_ representations of **14** with ALDH1A3 and **18** with ALDH3A1, inhibition kinetics curves of ALDH1A3 by **15** and **16**, IC_50_ representations of **18** with ALDH3A1 with and without NADP^+^, docking studies of compounds **14** and **18** on ALDH1A1, ALDH1A3 and ALDH3A1 isoforms and representations of the preliminary two-dose point antiproliferative data using PC3 cells and the MTT assay (PDF)

## Abbreviations

ADT: androgen therapy
ALDH: aldehyde dehydrogenase
AR: androgen receptor
BPH: benign prostate hyperplasia
^13^C NMR: carbon-13 nuclear magnetic resonance
CH_2_Cl_2_: dichloromethane
CH_3_OH: methanol
CRPC: castrate-resistant prostate cancer
CYP: cytochrome p450
DEAB: 4-(diethylamino)benzaldehyde
DMF: dimethylformamide
DMSO: dimethyl sulfoxide
DPBA: 4-(dipropylamino)benzaldehyde
EA: ethyl acetate
GLUT1: glucose transporter 1
^1^H NMR: proton nuclear magnetic resonance
HRMS: high resolution mass spectrometry
Hz: hertz
IC_50_: half-maximal inhibitory concentration
*K*_i_: inhibitory constant
mp: melting point
MHz: megahertz
NAD^+^: nicotinamide adenine dinucleotide
NADH: nicotinamide adenine dinucleotide hydrogen
4-NBA: 4-nitrobenzaldehyde
PCa: prostate cancer
PDB: protein data bank
PE: petroleum ether
RA: retinoic acid
*R*_f_: retention factor
RMSD: root-mean-square deviation
SCs: stem cells
CSCs: cancer stem cells
TLC: thin layer chromatography
