# Supplementary material for: Expansion of the 4-(Diethylamino)benzaldehyde Scaffold to Explore the Impact on Aldehyde Dehydrogenase Activity and Antiproliferative Activity in Prostate Cancer
Source: J Med Chem. 2022 Feb 25;65(5):3833–48. doi: 10.1021/acs.jmedchem.1c01367 (PMC9007462; doi:10.1021/acs.jmedchem.1c01367)
Supplement: Supplementary file 3 — jm1c01367_si_023.pdf [file jm1c01367_si_023.pdf]

Supporting Information

Expansion of the 4-(diethylamino)benzaldehyde (DEAB) scaffold to explore impact on aldehyde dehydrogenase activity and antiproliferative activity in prostate cancer

Ali I. M. Ibrahim,[1,2] Elisabet Batlle,[1,3] Smarakan Sneha,[1] Rafael Jiménez.,[3] Raquel Pequerul,[3] Xavier Parés,[3] Till Rüngeler,[3] Vibhu Jha,[4] Tiziano Tuccinardi,[4] Maria Sadiq,[1,5] Fiona Frame,[5] Norman J. Maitland,[5] Jaume Farrés,[3*] Klaus Pors[1*]

[1] Institute of Cancer Therapeutics, School of Pharmacy and Medical Sciences, Faculty of Life Sciences, University of Bradford, West Yorkshire BD7 1DP, U.K.
[2] Faculty of Pharmacy, Al-Zaytoonah University of Jordan, Amman 11733, Jordan.
[3] Department of Biochemistry and Molecular Biology, Faculty of Biosciences, Universitat Autònoma de Barcelona, E-08193, Bellaterra, Barcelona, Spain.
[4] Department of Pharmacy, University of Pisa, Via Bonanno 6, 56126 Pisa, Italy.
[5] Cancer Research Unit, Department of Biology, University of York, Heslington, North Yorkshire YO10 5DD, U.K.

* Corresponding author. E-mail: jaume.farres@uab.cat (Jaume Farrés)
* Corresponding author. E-mail: k.Pors1@bradford.ac.uk (Klaus Pors)

Table of Content

Supplementary Figures

Kinetic supplementary figures

Remaining ALDHs activities (%) in the presence of 40 DEAB analogues S2

Substrate activity for the 40 DEAB analogues S3

IC_{50} representation of analogues **14** with ALDH1A3 and **18** with ALDH3A1 S4

K_i representations of analogue **15** with ALDH1A3 S4

K_i representations of analogue **16** with ALDH1A3 S5

IC_{50} representation of analogue **18** with ALDH3A1, with and without incubation with the $NADP^+$ S5

Docking studies figures S6

Biological testing figures

Two-dose point antiproliferative data of lead analogues using the MTT assay. S9

HPLC traces of compounds S13

SUPPLEMENTARY FIGURES

Kinetic supplementary figures

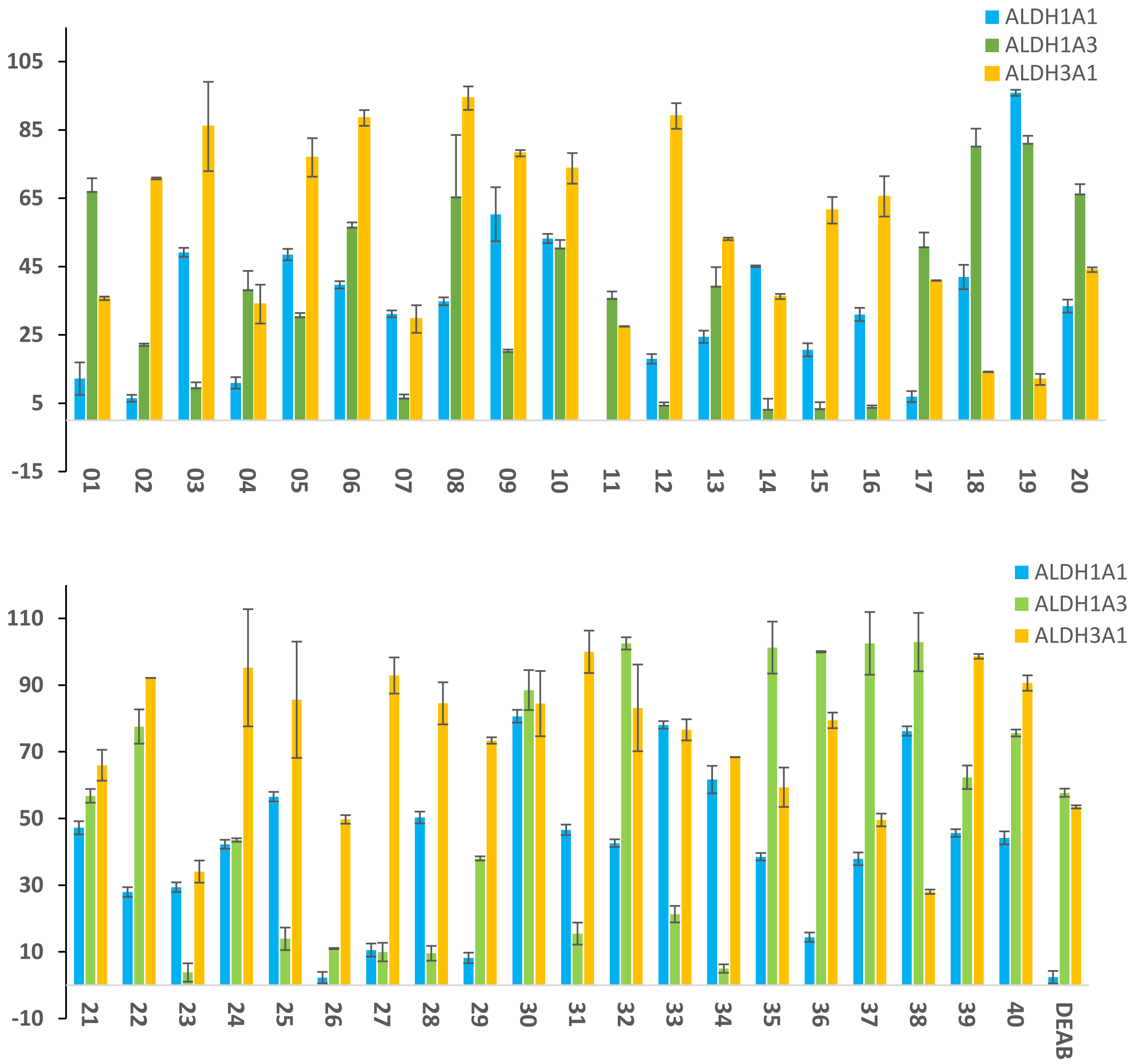

Figure S1. Remaining ALDHs activities (%) in the presence of DEAB derivatives **1** to **40**. The fluorescence of NAD(P)H produced during the reaction (excitation at 340 nm and emission at 460 nm) was measured in absence and presence of 10μM of inhibitor, using hexanal or 4-NBA as substrates. The changes in the slope are represented as the % of residual activity of duplicate experiments ± SD. Conditions of the assay are described in the experimental section of the manuscript.

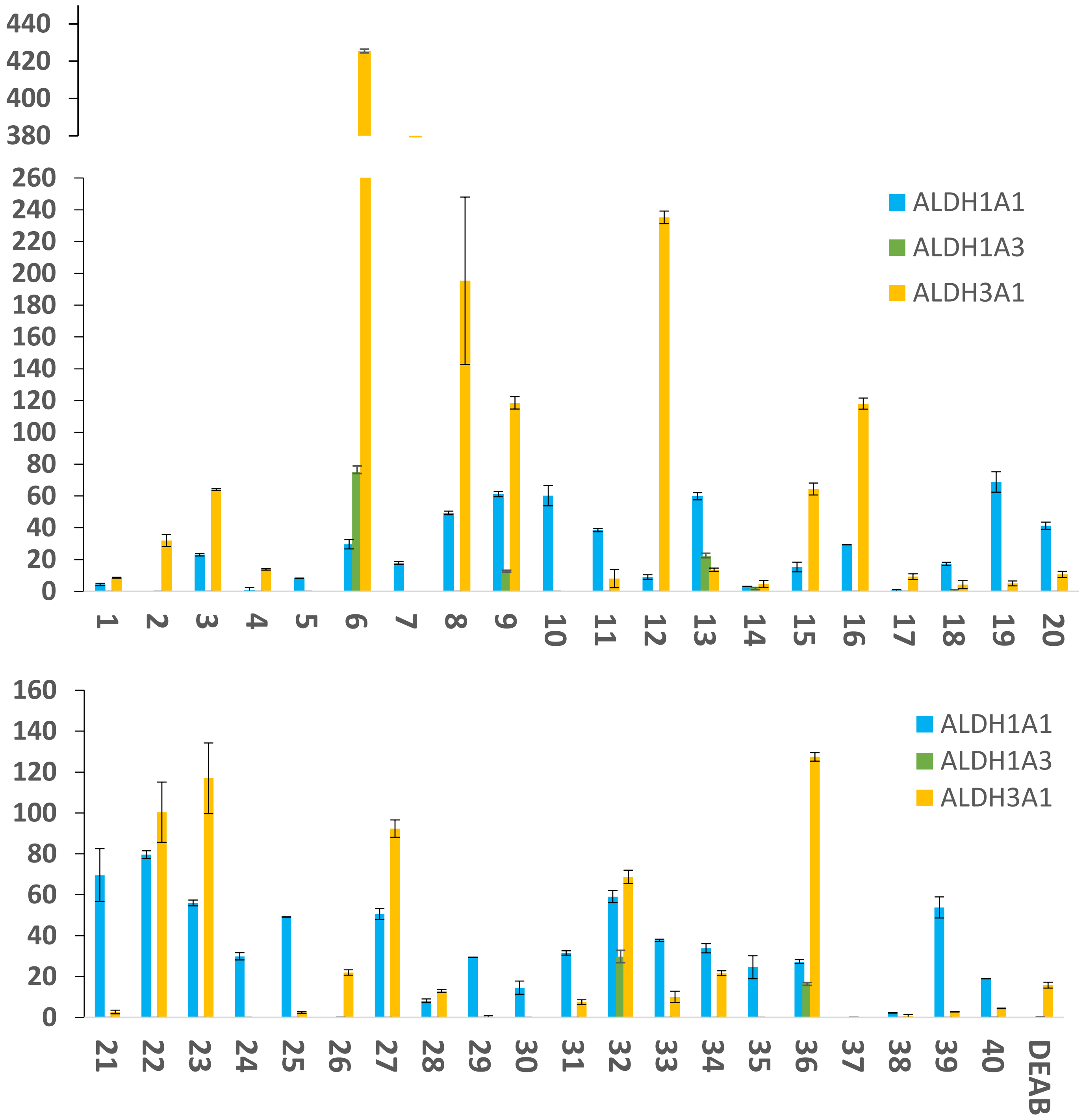

Figure S2. Enzymatic activity(%) using the **40** DEAB analogues as substrates. Conditions were the same as Figure S1 but using the DEAB analogues as substrates instead of hexanal or 4-NBA Enzyme activity ratio is represented as the activity observed with 10 μM of compound used as substrate against the activity observed using 10 μM of the standard substrate ± SD (%)

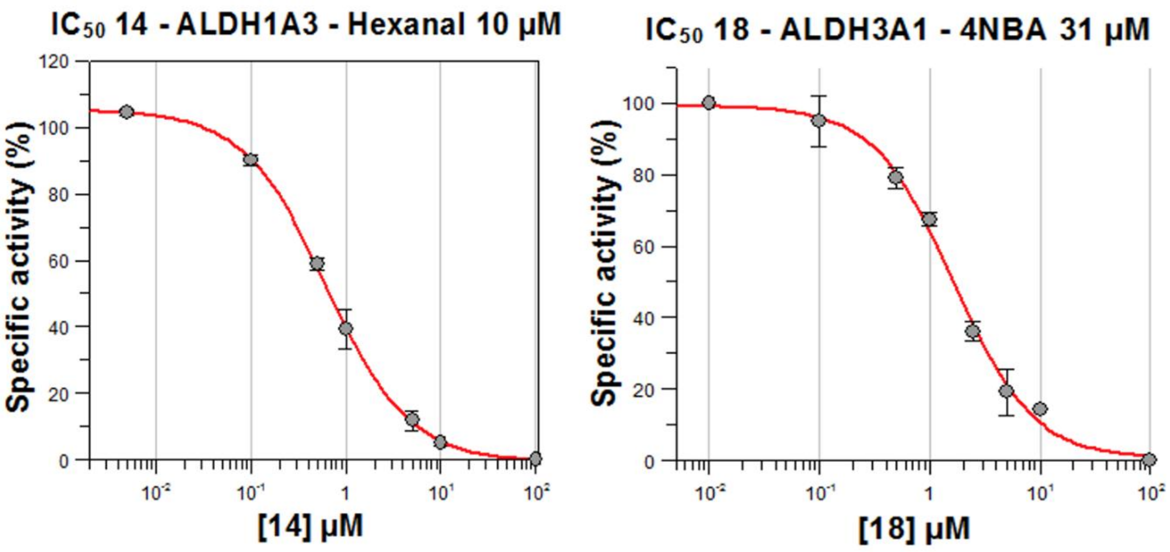

Figure S3. Representative sigmoidal curves to calculate the IC_{50} values of **14** with ALDH1A3 and **18** with ALDH3A1. The percentage of specific activity is plotted against the logarithm of inhibitor concentration. Experimental values are shown as the mean ± SE.

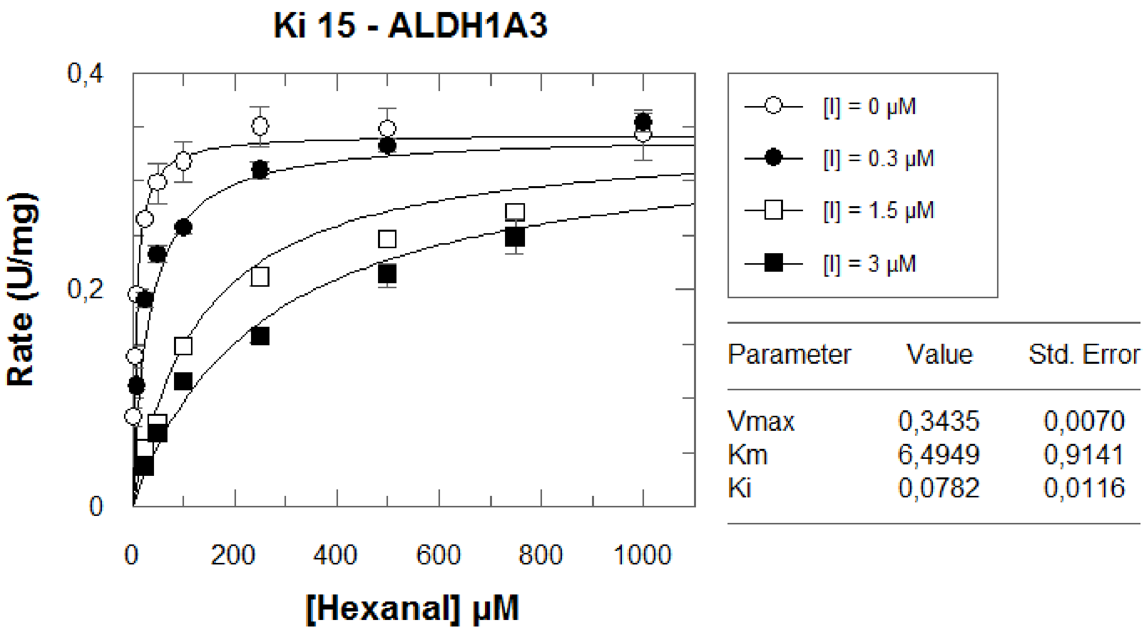

Figure S4. Inhibition kinetics of ALDH1A3 by **15** at various concentration of inhibitor, using hexanal as the substrate. The values of the kinetic parameters are calculated from a competitive inhibition fit. Results are the mean ± SE of duplicate experiments.

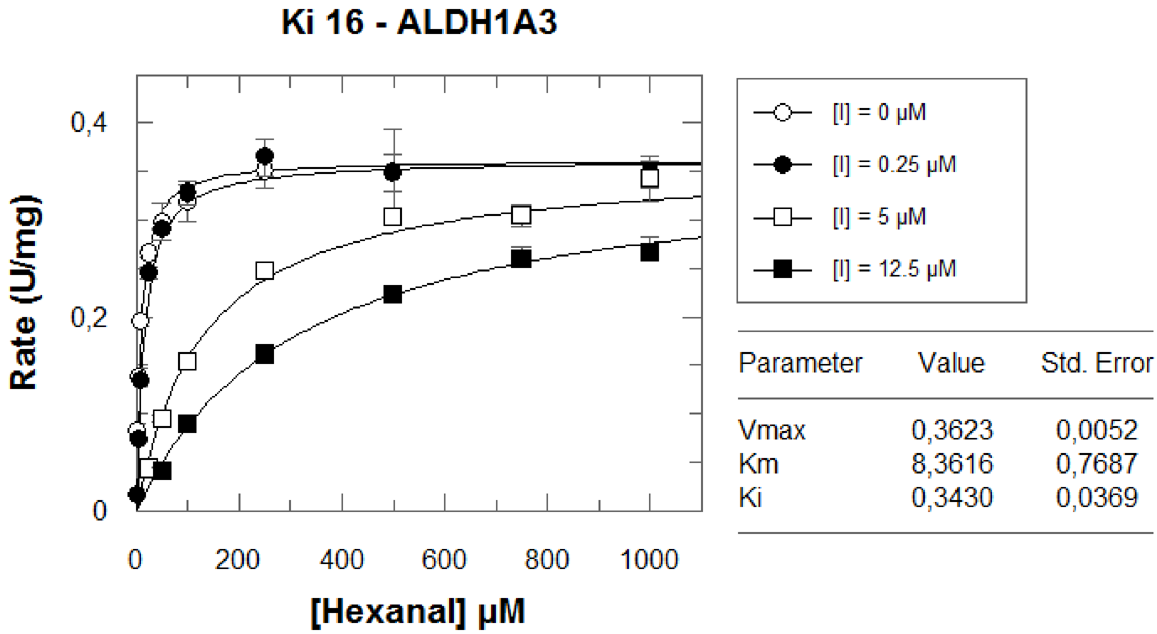

Figure S5. Inhibition kinetics of ALDH1A3 by **16** at various concentration of inhibitor, using hexanal as the substrate. The values of the kinetic parameters are calculated from a competitive inhibition fit. Results are the mean ± SE of duplicate experiments.

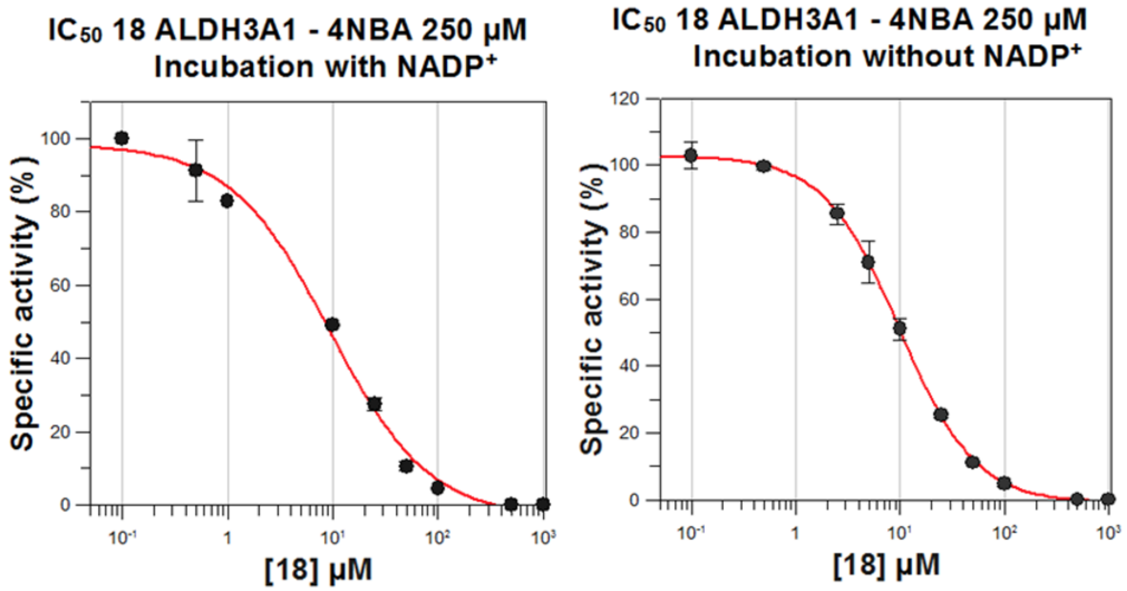

Figure S6. IC_{50} values for **18** at saturating concentration of 4-NBA, with and without incubation with the $NADP^+$. Percentage of specific activity is plotted against the logarithm of inhibitor concentration. Experimental values are indicated as the mean of duplicate experiments ± SE.

Docking studies figures

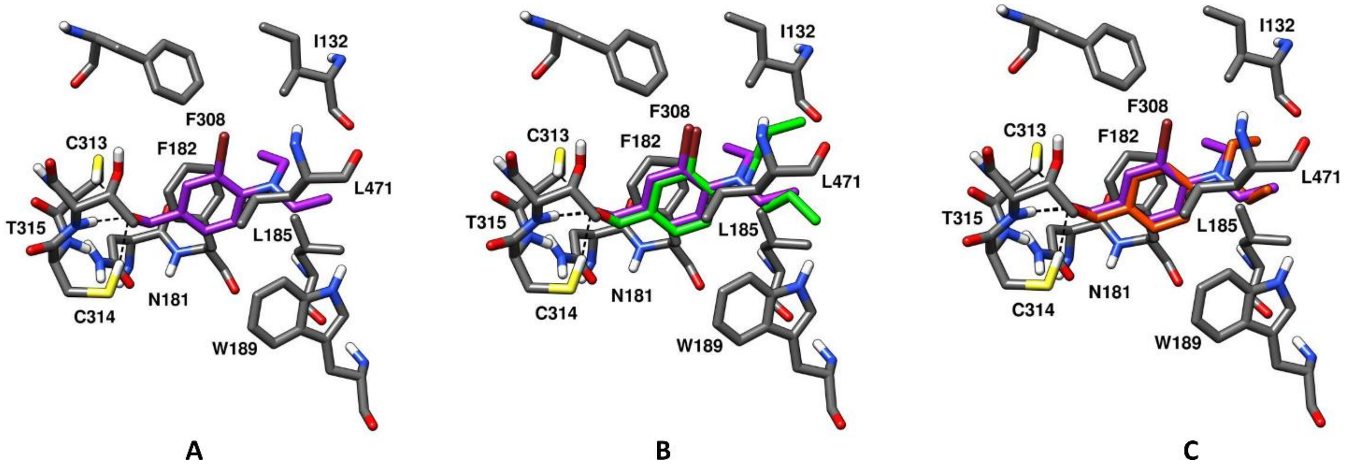

Figure S7. Molecular docking of (A) **13** (purple), (B) **13** (purple) and **14** (green), (C) **13** (purple) and DEAB (orange) into ALDH1A3 binding site (PDB ID: 5FHZ).

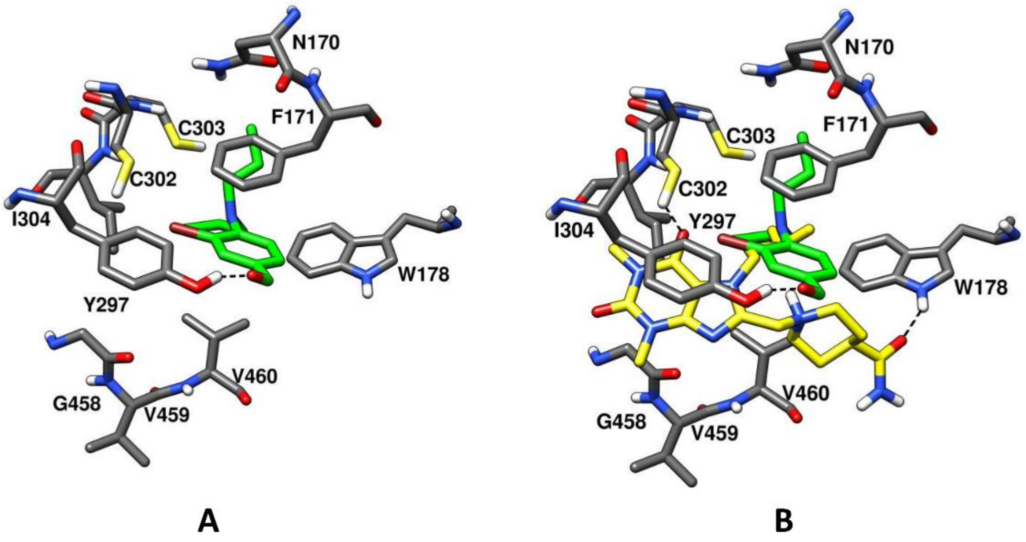

Figure S8. Molecular docking of (A) **14** (green), (B) **14** (green) and the co-crystallized inhibitor (yellow) into ALDH1A1 binding site (PDB ID: 4WPN).

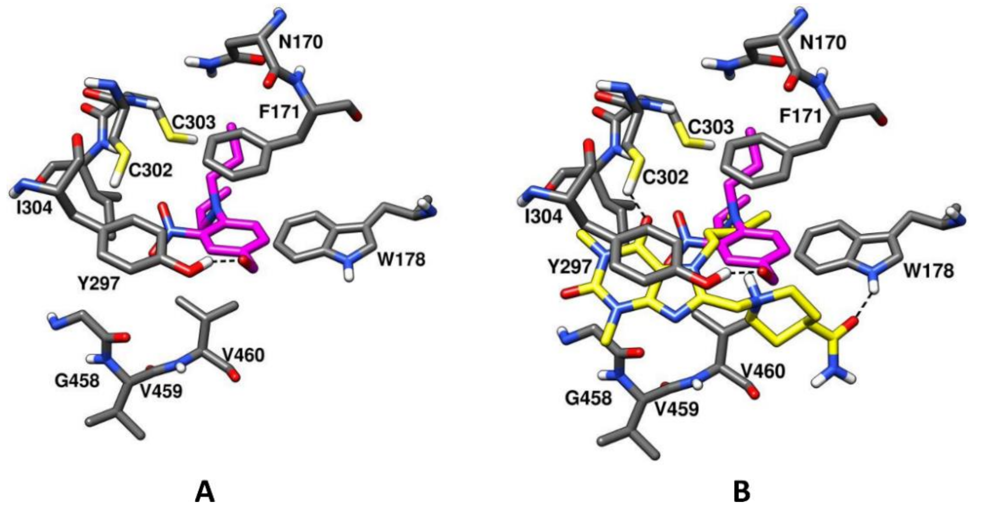

Figure S9. Molecular docking of (A) **18** (magenta), (B) **18** (magenta) and the co-crystallized inhibitor (yellow) into ALDH1A1 binding site (PDB ID: 4WPN).

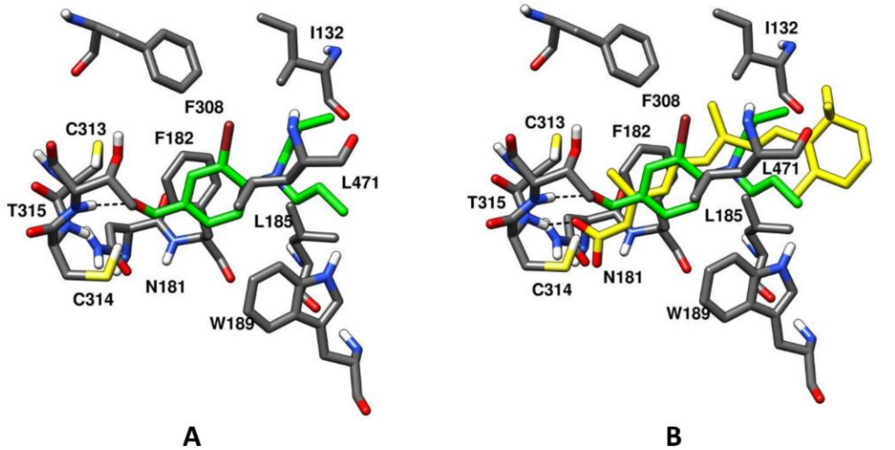

Figure S10. Molecular docking of (A) **14** (green), (B) **14** (green) and retinoic acid (yellow) into ALDH1A3 binding site (PDB ID: 5FHZ).

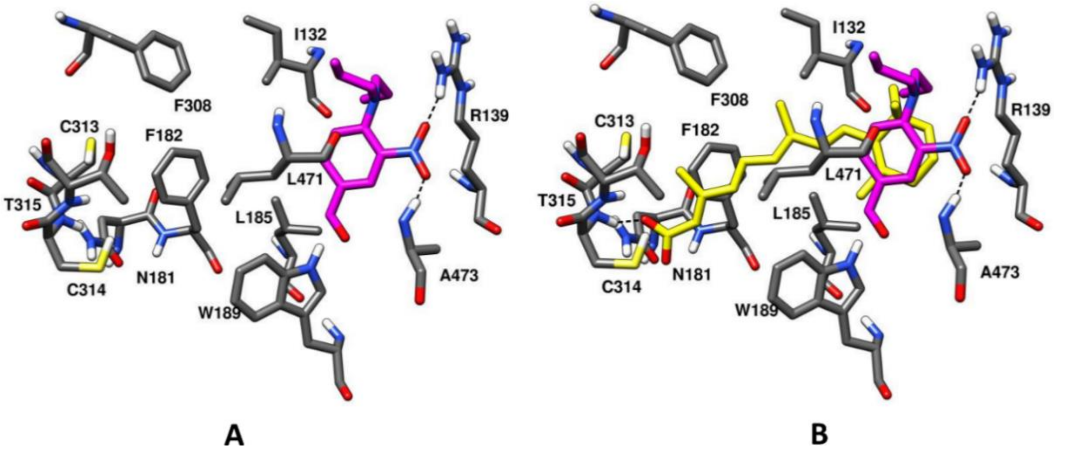

Figure S11. Molecular docking of (A) **18** (magenta), (B) **18** (magenta) and the retinoic acid (yellow) into ALDH1A3 binding site (PDB ID: 5FHZ).

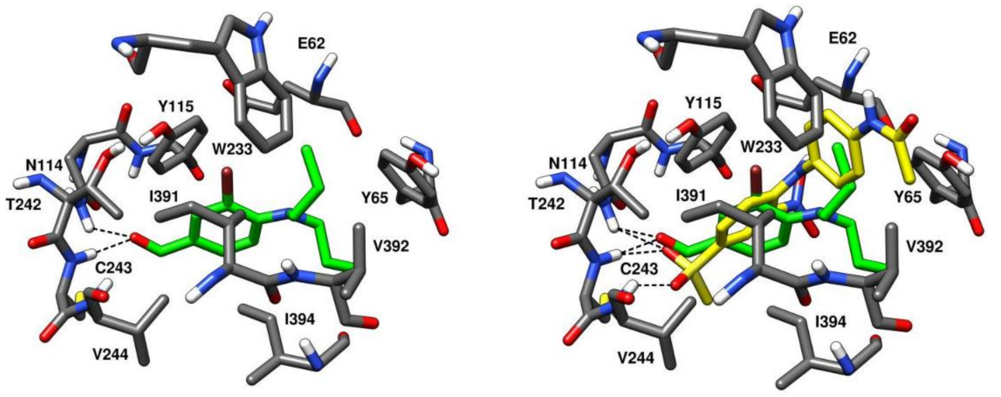

A B

Figure S12. Molecular docking of (A) **14** (green), (B) **14** (green) and the co-crystallized inhibitor (yellow) into ALDH3A1 binding site (PDB ID: 4H80).

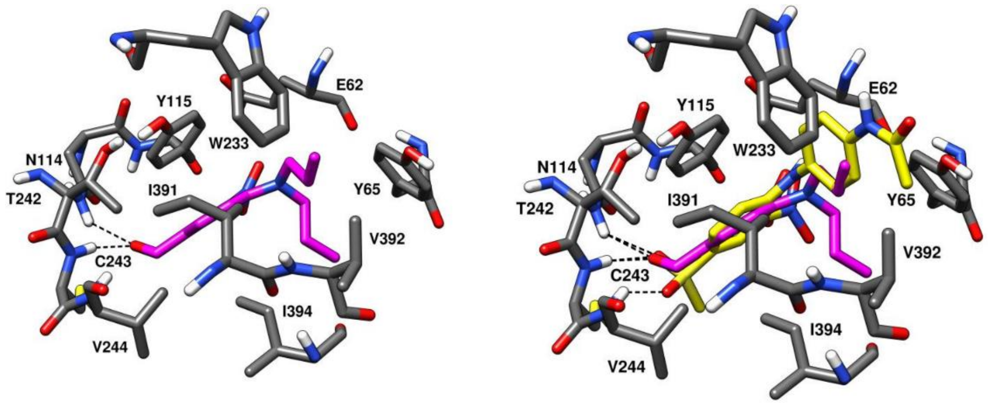

A B

Figure S13. Molecular docking of (A) **18** (magenta), (B) **18** (magenta) and the co-crystallized inhibitor (yellow) into ALDH3A1 binding site (PDB ID: 4H80).

Biological testing figures

1

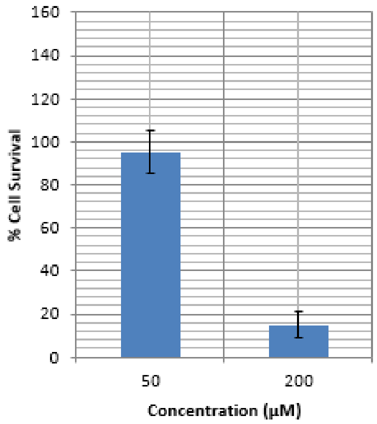

2

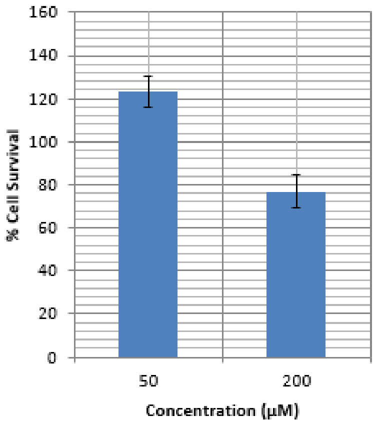

3

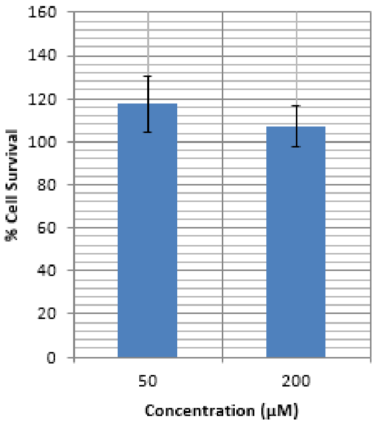

4

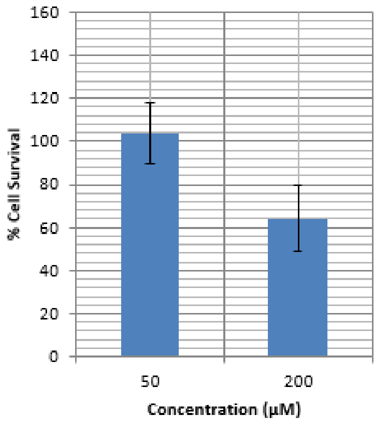

5

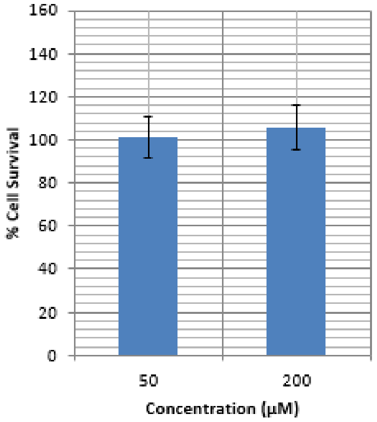

6

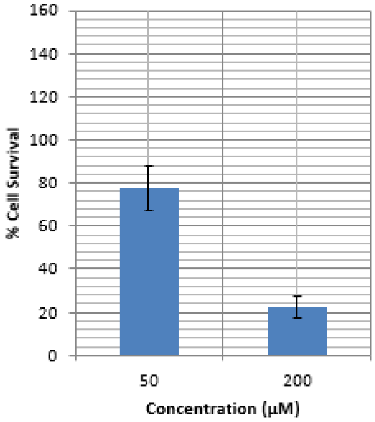

7

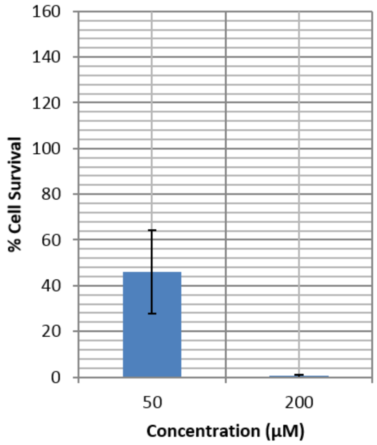

8

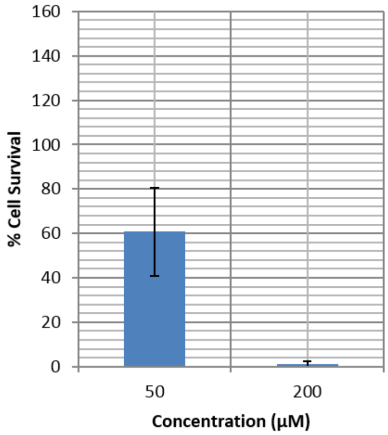

9

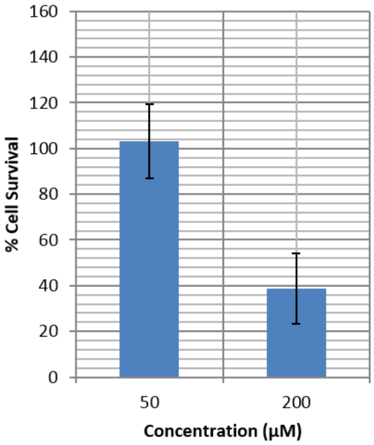

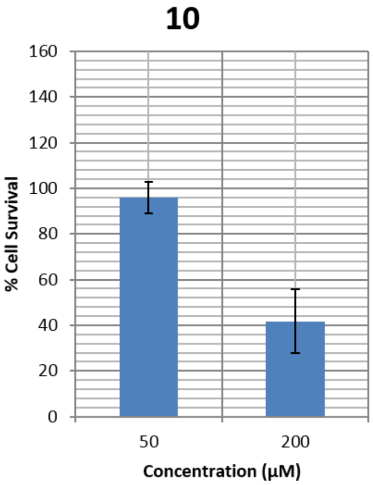
10
160
140
120
100
80
60
40
20
0
% Cell Survival
50
200
Concentration (μM)

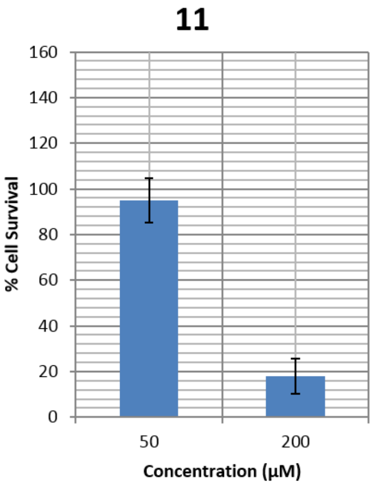
11
160
140
120
100
80
60
40
20
0
% Cell Survival
50
200
Concentration (μM)

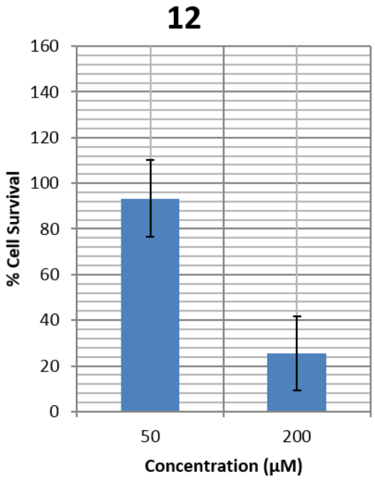
12
160
140
120
100
80
60
40
20
0
% Cell Survival
50
200
Concentration (μM)

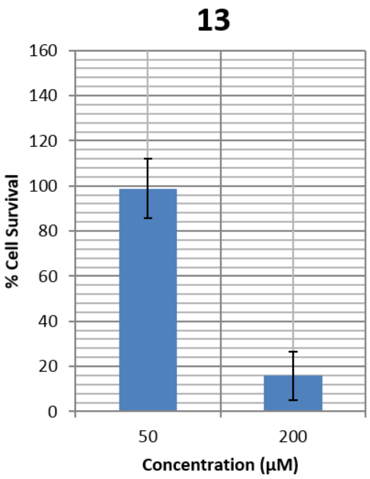
13
160
140
120
100
80
60
40
20
0
% Cell Survival
50
200
Concentration (μM)

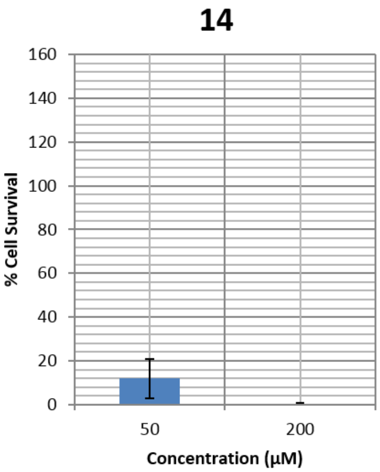
14
160
140
120
100
80
60
40
20
0
% Cell Survival
50
200
Concentration (μM)

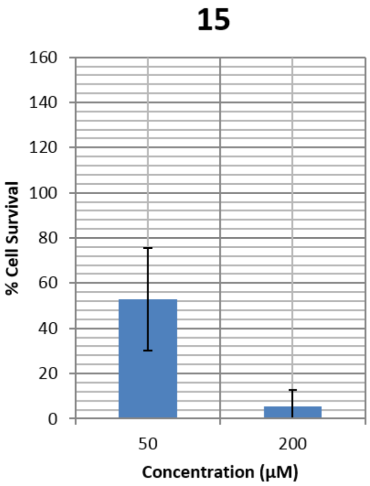
15
160
140
120
100
80
60
40
20
0
% Cell Survival
50
200
Concentration (μM)

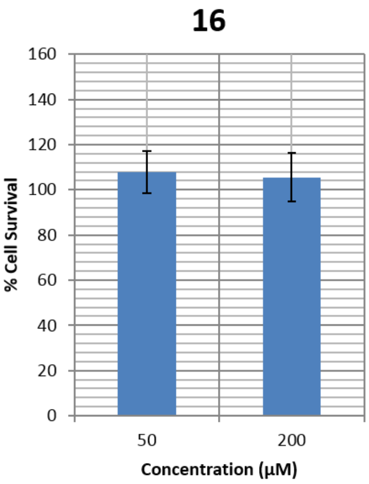
16
160
140
120
100
80
60
40
20
0
% Cell Survival
50
200
Concentration (μM)

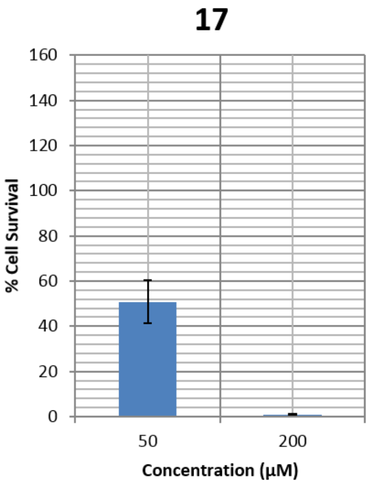
17
160
140
120
100
80
60
40
20
0
% Cell Survival
50
200
Concentration (μM)

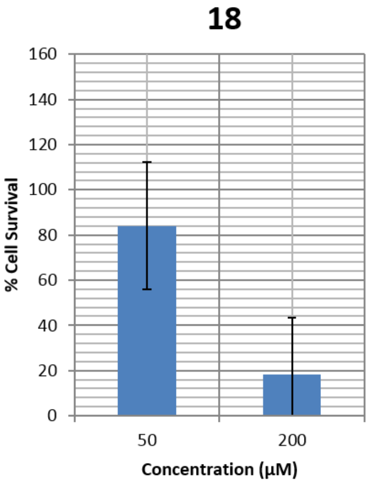
18
160
140
120
100
80
60
40
20
0
% Cell Survival
50
200
Concentration (μM)

19

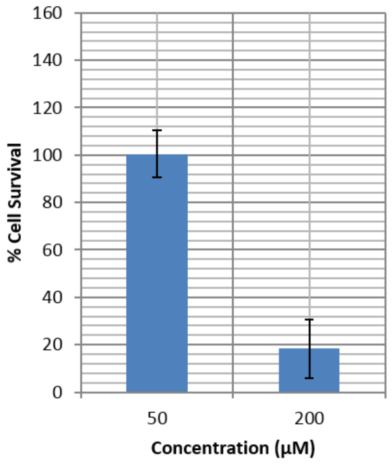

20

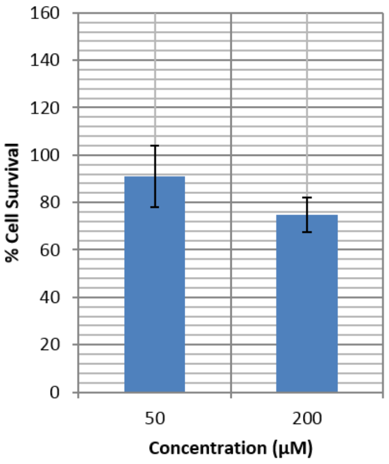

21

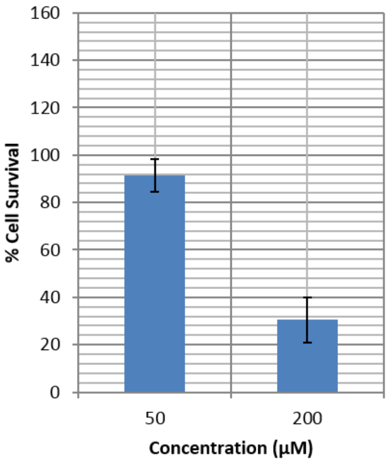

22

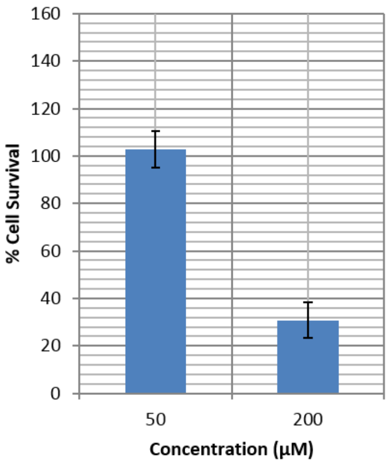

23

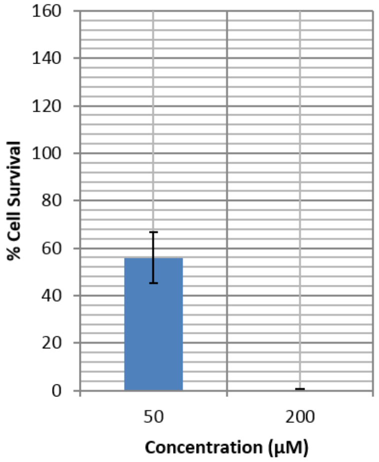

24

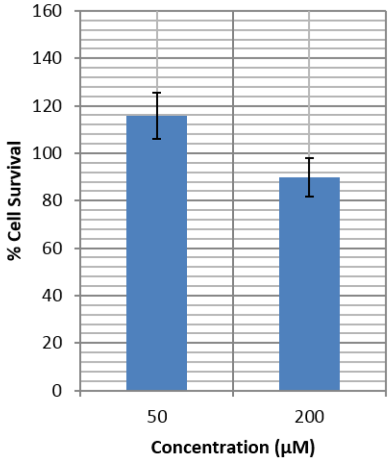

25

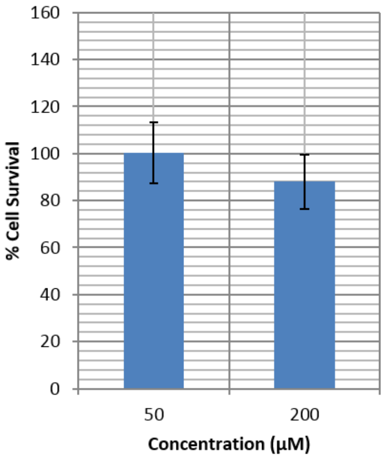

26

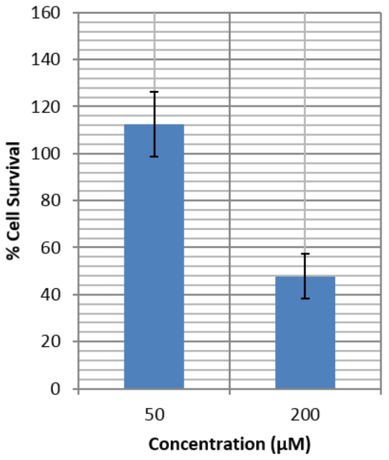

27

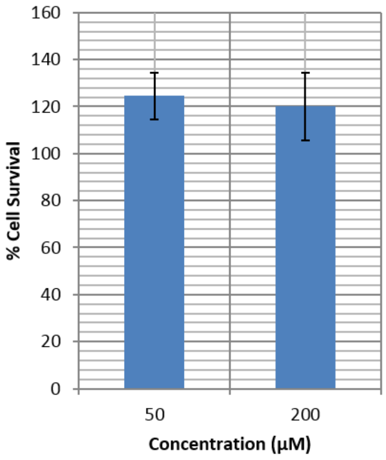

28

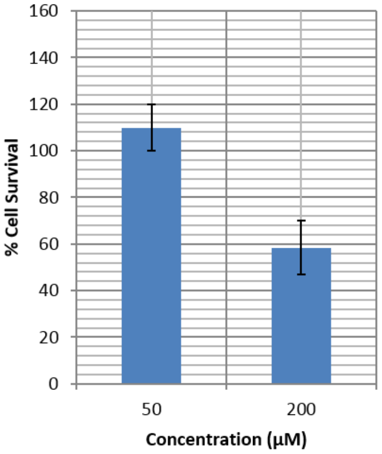

29

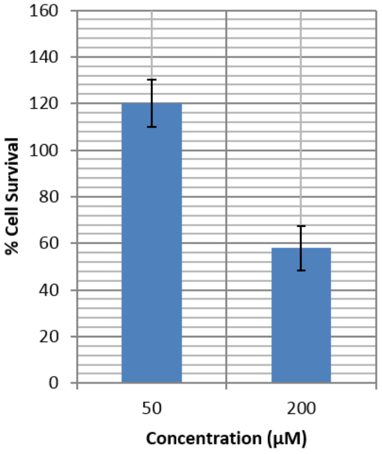

30

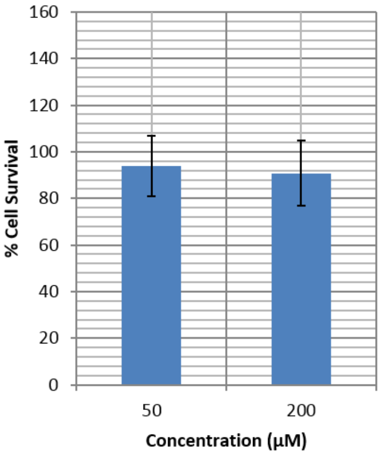

31

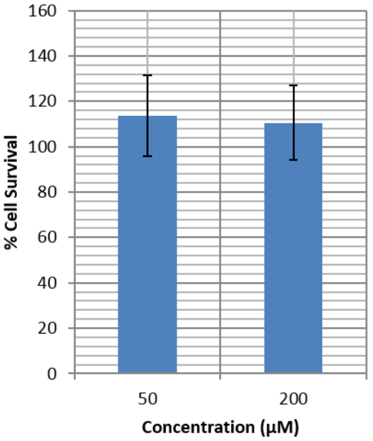

32

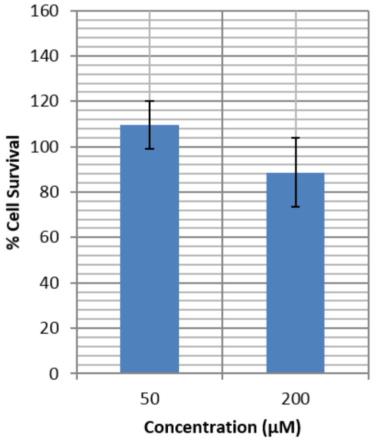

33

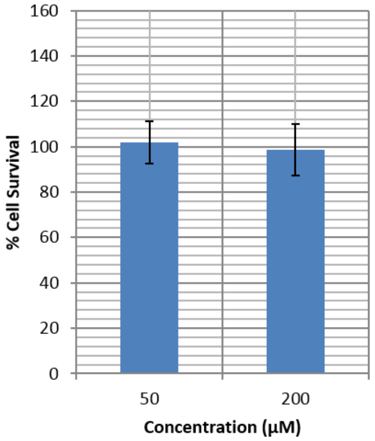

34

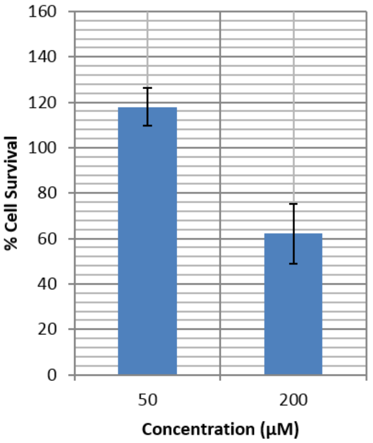

35

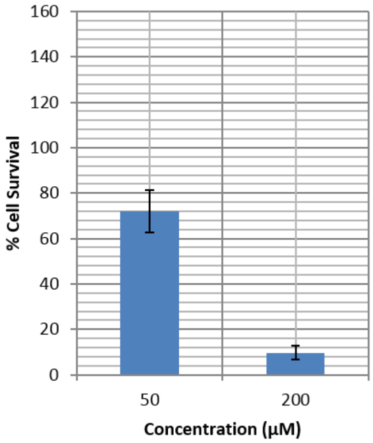

36

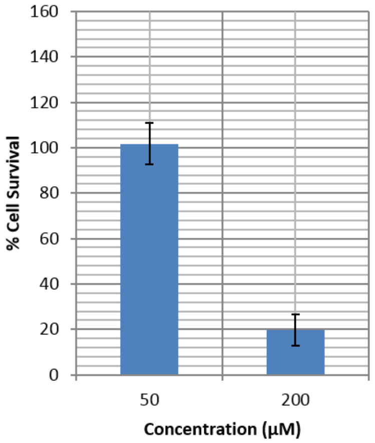

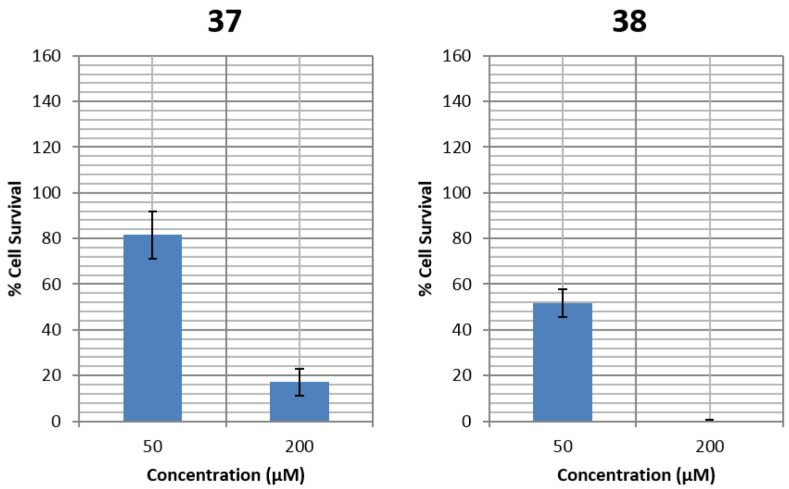

Figure S14. Two-dose point antiproliferative screen (MTT assay) exploring DEAB analogue library for dose-dependent effect in PC-3 prostate cancer cell line.

HPLC TRACES FOR COMPOUNDS

4-(dipropylamino)benzaldehyde (1) :

Additional Info : Peak(s) manually integrated

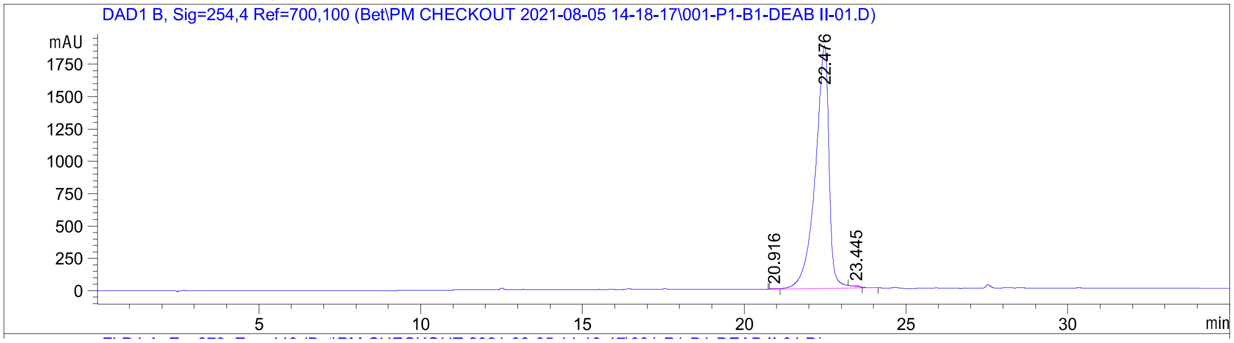

Signal 1: DAD1 B, Sig=254,4 Ref=700,100

Peak #	RetTime [min]	Type	Width [min]	Area [mAU*s]	Height [mAU]	Area %
1	20.916	BV E	0.1429	14.31065	1.56113	0.0257
2	22.476	VV R	0.4355	5.55644e4	1876.28784	99.8601
3	23.445	VB E	0.1573	63.54439	6.01465	0.1142

4-(piperidin-1-yl)benzaldehyde (2) :

Additional Info : Peak(s) manually integrated

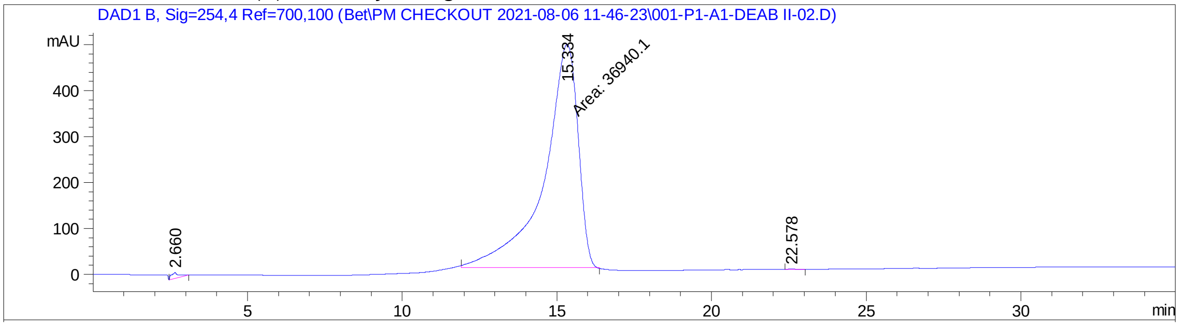

Signal 1: DAD1 B, Sig=254,4 Ref=700,100

Peak #	RetTime [min]	Type	Width [min]	Area [mAU*s]	Height [mAU]	Area %
1	2.660	BB	0.2122	216.20990	12.72620	0.5817
2	15.334	MM	1.2674	3.69401e4	485.78403	99.3843
3	22.578	BB	0.1746	12.63018	1.07849	0.0340

4-morpholinobenzaldehyde (3) :

Additional Info : Peak(s) manually integrated

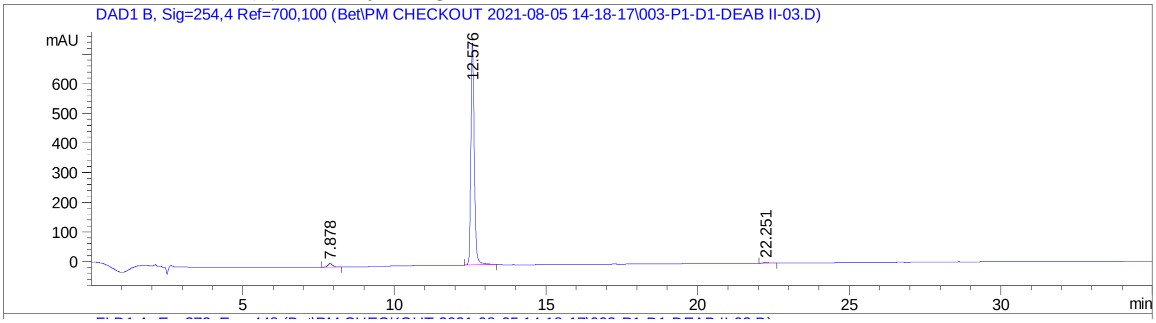

Signal 1: DAD1 B, Sig=254,4 Ref=700,100

Peak #	RetTime [min]	Type	Width [min]	Area [mAU*s]	Height [mAU]	Area %
1	7.878	BB	0.1629	124.25298	11.79721	2.0599
2	12.576	BB	0.1194	5869.52637	749.43933	97.3052
3	22.251	BB	0.1679	38.29994	3.49297	0.6349

4-(pyrrolidin-1-yl)benzaldehyde (4) :

Additional Info : Peak(s) manually integrated

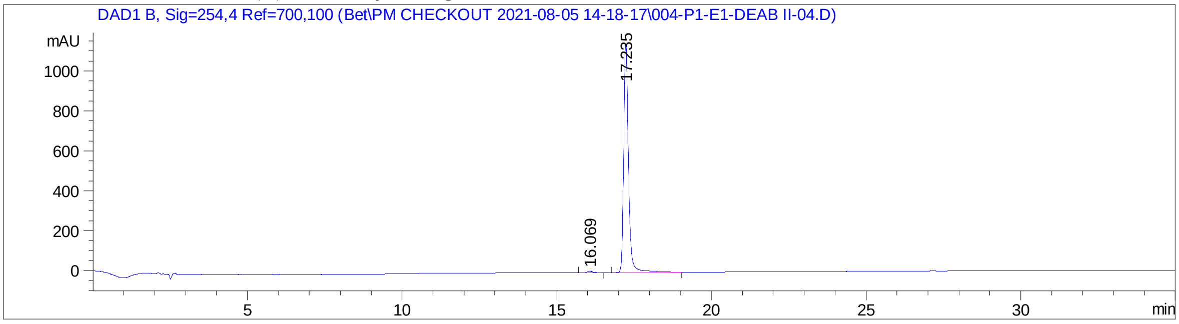

Signal 1: DAD1 B, Sig=254,4 Ref=700,100

Peak #	RetTime [min]	Type	Width [min]	Area [mAU*s]	Height [mAU]	Area %
1	16.069	BB	0.1633	81.63882	7.60382	0.6613
2	17.235	BB	0.1631	1.22641e4	1144.18396	99.3387

4-(4-methylpiperazin-1-yl)benzaldehyde (5) :

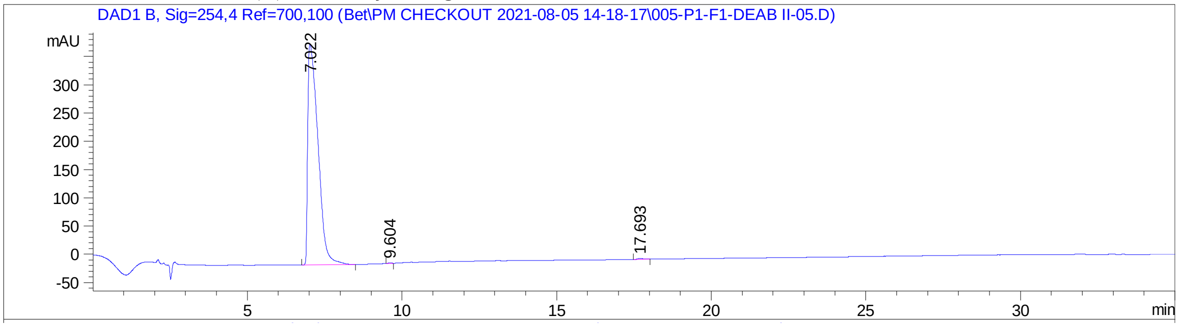

Signal 1: DAD1 B, Sig=254,4 Ref=700,100

Peak #	RetTime [min]	Type	Width [min]	Area [mAU*s]	Height [mAU]	Area %
1	7.022	BB	0.3798	8974.63574	390.99802	99.6859
2	9.604	BB	0.1020	7.01952	1.07803	0.0780
3	17.693	BB	0.1797	21.26141	1.75033	0.2362

4-(1H-imidazol-1-yl)benzaldehyde (6) :

Additional Info : Peak(s) manually integrated

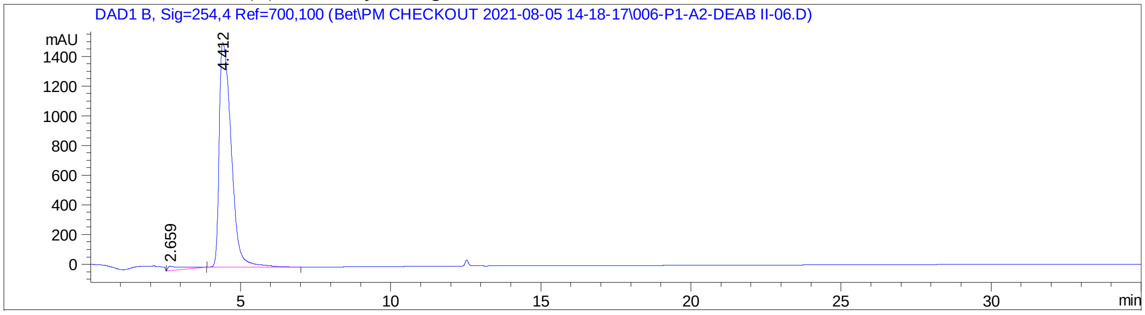

Peak #	RetTime [min]	Type	Width [min]	Area [mAU*s]	Height [mAU]	Area %
1	2.659	BB	0.4623	1024.65027	28.17360	2.3760
2	4.412	BB	0.4432	4.21011e4	1510.53052	97.6240

Signal 1: DAD1 B, Sig=254,4 Ref=700,100

3-chloro-4-(dipropylamino)benzaldehyde (7) :

Additional Info : Peak(s) manually integrated

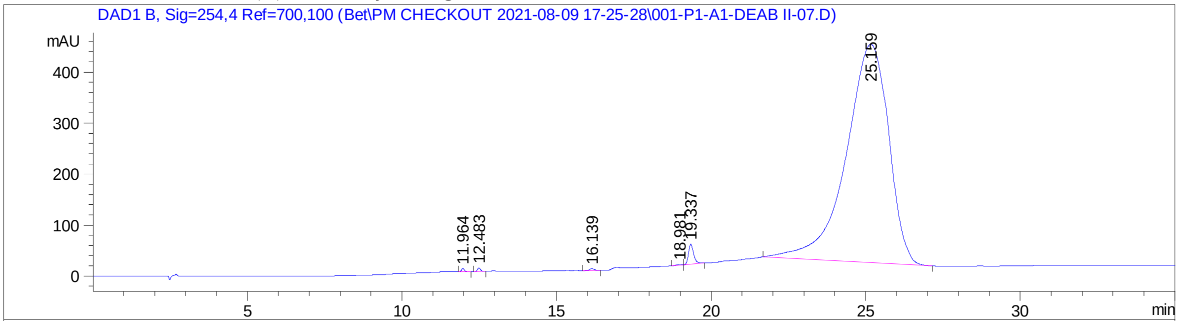

Signal 1: DAD1 B, Sig=254,4 Ref=700,100

Peak #	RetTime [min]	Type	Width [min]	Area [mAU*s]	Height [mAU]	Area %
1	11.964	BB	0.1092	43.71052	6.12898	0.1053
2	12.483	BB	0.1129	52.23473	7.17571	0.1258
3	16.139	BB	0.1605	34.88321	3.32226	0.0840
4	18.981	BB	0.1804	15.32135	1.32952	0.0369
5	19.337	BB	0.1732	445.13574	38.97570	1.0722
6	25.159	BB	1.3816	4.09258e4	427.98630	98.5758

3-chloro-4-(piperidin-1-yl)benzaldehyde (8):

Additional Info : Peak(s) manually integrated

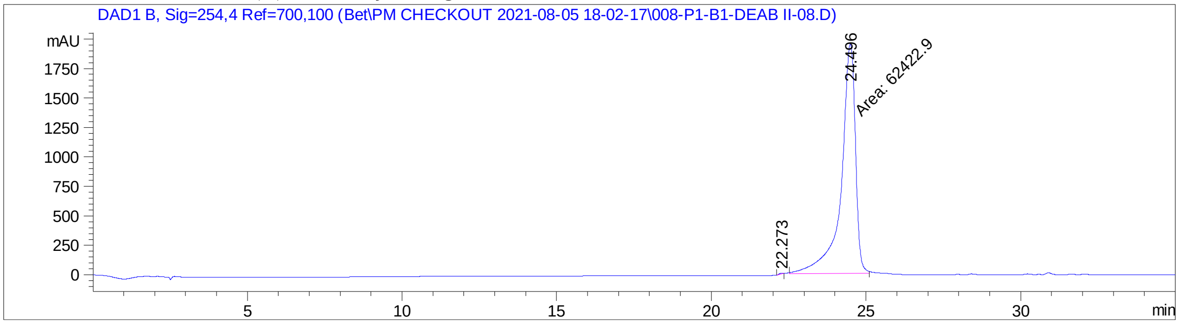

Signal 1: DAD1 B, Sig=254,4 Ref=700,100

Peak #	RetTime [min]	Type	Width [min]	Area [mAU*s]	Height [mAU]	Area %
1	22.273	BB	0.1337	51.63015	6.04026	0.0826
2	24.496	MM	0.5352	6.24229e4	1943.79456	99.9174

3-chloro-4-morpholinobenzaldehyde (9) :

Additional Info : Peak(s) manually integrated

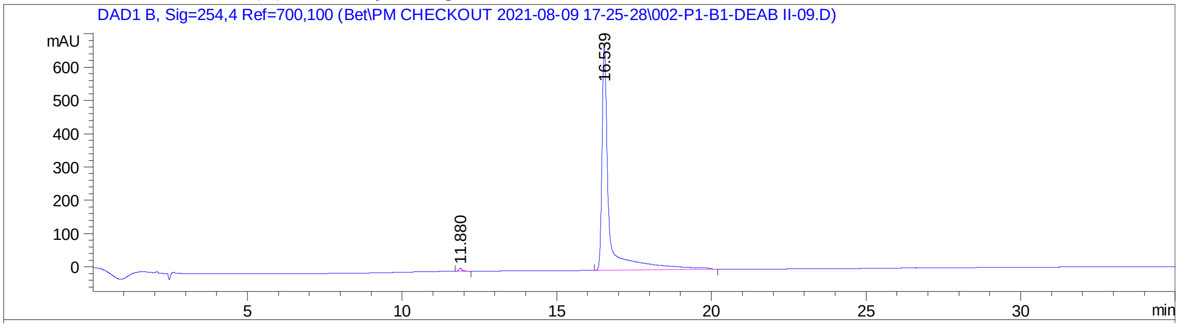

Signal 1: DAD1 B, Sig=254,4 Ref=700,100

Peak #	RetTime [min]	Type	Width [min]	Area [mAU*s]	Height [mAU]	Area %
1	11.880	BB	0.1160	67.64488	8.76545	0.6158
2	16.539	BB	0.2276	1.09173e4	678.97278	99.3842

3-chloro-4-(4-methylpiperazin-1-yl)benzaldehyde (10) :

Additional Info : Peak(s) manually integrated

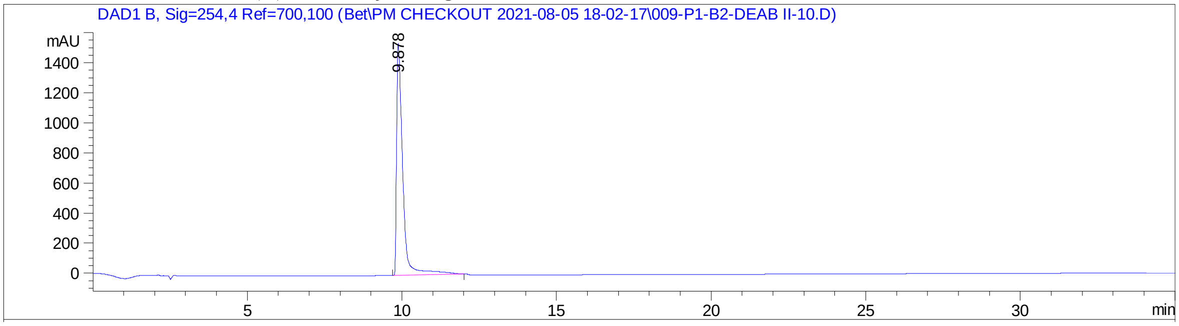

Signal 1: DAD1 B, Sig=254,4 Ref=700,100

Peak #	RetTime [min]	Type	Width [min]	Area [mAU*s]	Height [mAU]	Area %
1	9.878	BB	0.2052	2.12655e4	1539.51758	100.0000

3-chloro-4-(diethylamino)benzaldehyde (11):

Additional Info : Peak(s) manually integrated

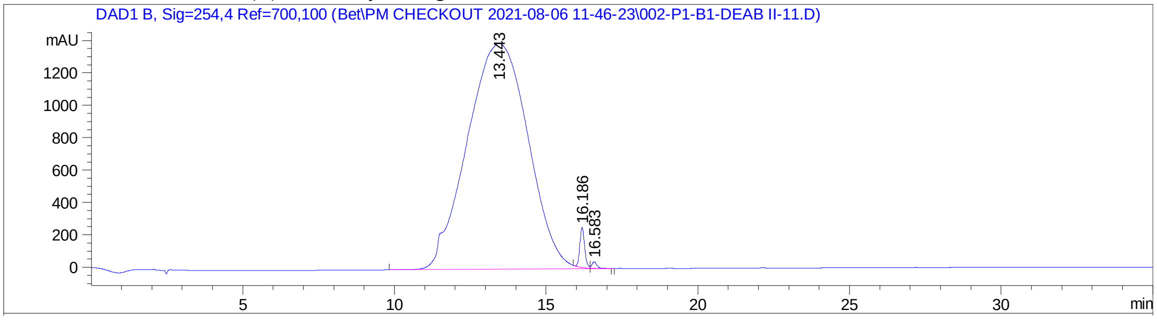

Signal 1: DAD1 B, Sig=254,4 Ref=700,100

Peak #	RetTime [min]	Type	Width [min]	Area [mAU*s]	Height [mAU]	Area %
1	13.443	BV R	1.9258	1.94126e5	1394.72510	98.3761
2	16.186	VV E	0.1688	2738.69507	248.08241	1.3879
3	16.583	VB E	0.1717	465.79083	40.64745	0.2360

3-chloro-4-(pyrrolidin-1-yl)benzaldehyde (12):

Additional Info : Peak(s) manually integrated

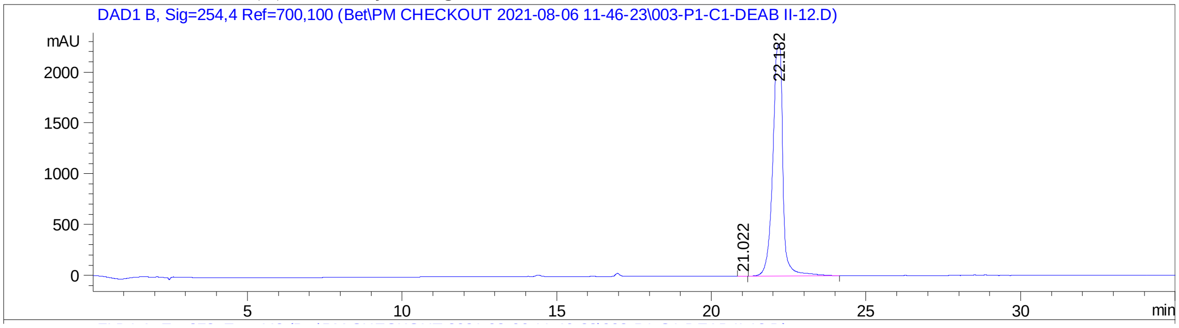

Signal 1: DAD1 B, Sig=254,4 Ref=700,100

Peak #	RetTime [min]	Type	Width [min]	Area [mAU*s]	Height [mAU]	Area %
1	21.022	BB	0.1413	12.34792	1.36748	0.0239
2	22.182	BB	0.3422	5.16156e4	2279.48999	99.9761

3-bromo-4-(diethylamino)benzaldehyde (13) :

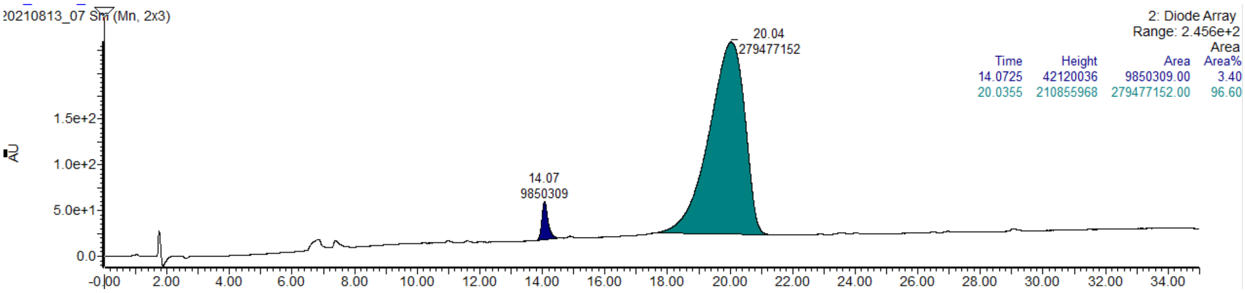

3-bromo-4-(dipropylamino)benzaldehyde (14):

Additional Info : Peak(s) manually integrated

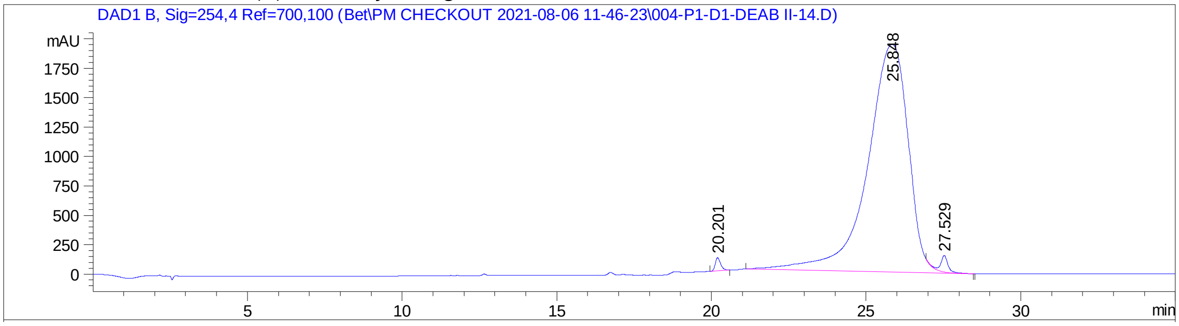

Signal 1: DAD1 B, Sig=254,4 Ref=700,100

Peak #	RetTime [min]	Type	Width [min]	Area [mAU*s]	Height [mAU]	Area %
1	20.201	BB	0.1865	1366.53235	111.89959	0.7542
2	25.848	BV R	1.3710	1.77454e5	1936.59839	97.9435
3	27.529	VB E	0.2461	2359.45093	140.37074	1.3023

3-methyl-4-(piperidin-1-yl)benzaldehyde (15) :

Additional Info : Peak(s) manually integrated

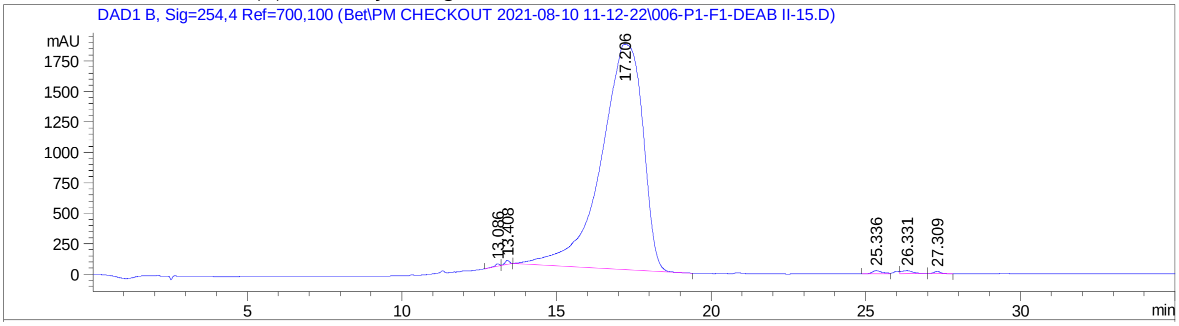

Signal 1: DAD1 B, Sig=254,4 Ref=700,100

Peak #	RetTime [min]	Type	Width [min]	Area [mAU*s]	Height [mAU]	Area %
1	13.086	BV	0.1577	233.68530	21.68569	0.1327
2	13.408	VB	0.1576	348.50089	33.98413	0.1979
3	17.206	BB	1.1617	1.74048e5	1849.82263	98.8169
4	25.336	BV	0.3194	593.99731	26.71027	0.3372
5	26.331	VB	0.3786	638.12390	24.93122	0.3623

4-isopropoxybenzaldehyde (16) :

Additional Info : Peak(s) manually integrated

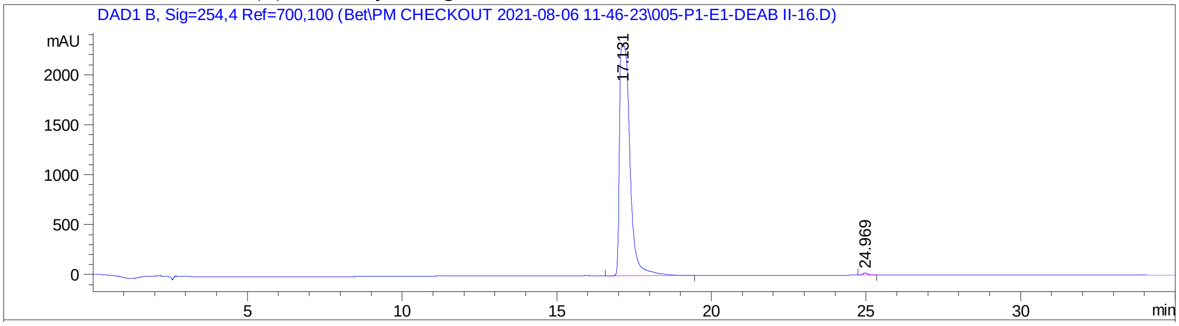

Signal 1: DAD1 B, Sig=254,4 Ref=700,100

Peak #	RetTime [min]	Type	Width [min]	Area [mAU*s]	Height [mAU]	Area %
1	17.131	BB	0.3682	5.44062e4	2314.95996	99.5705
2	24.969	BB	0.1617	234.68228	22.13058	0.4295

4-(dipropylamino)-3-methoxybenzaldehyde (17) :

Additional Info : Peak(s) manually integrated

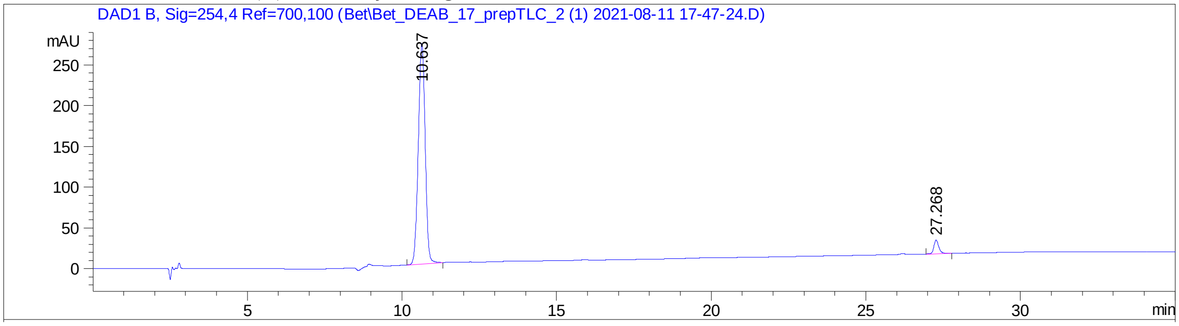

Signal 1: DAD1 B, Sig=254,4 Ref=700,100

Peak #	RetTime [min]	Type	Width [min]	Area [mAU*s]	Height [mAU]	Area %
1	10.637	BB	0.2426	4176.74902	269.87900	95.7622
2	27.268	BB	0.1649	184.83427	16.99200	4.2378

4-(dipropylamino)-3-nitrobenzaldehyde (18) :

Additional Info : Peak(s) manually integrated

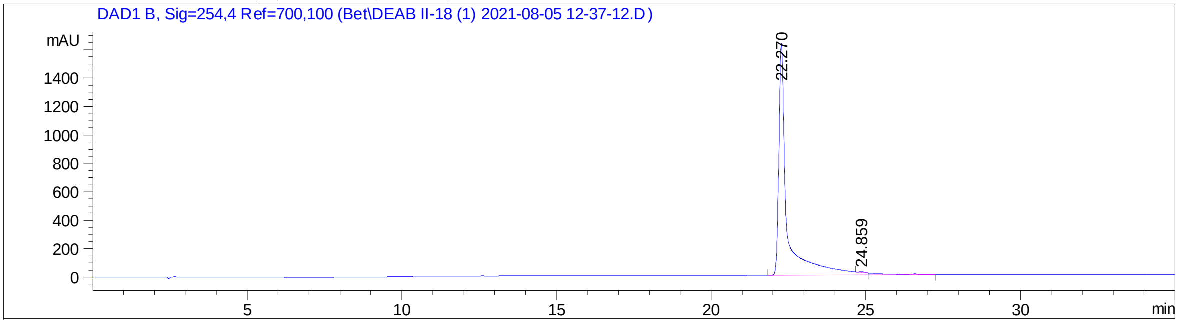

Signal 1: DAD1 B, Sig=254,4 Ref=700,100

Peak #	RetTime [min]	Type	Width [min]	Area [mAU*s]	Height [mAU]	Area %
1	22.270	BV R	0.2619	3.08126e4	1631.93091	99.8060
2	24.859	VV E	0.1542	59.90747	6.01718	0.1940

4-(diethylamino)-3-nitrobenzaldehyde (19) :

Additional Info : Peak(s) manually integrated

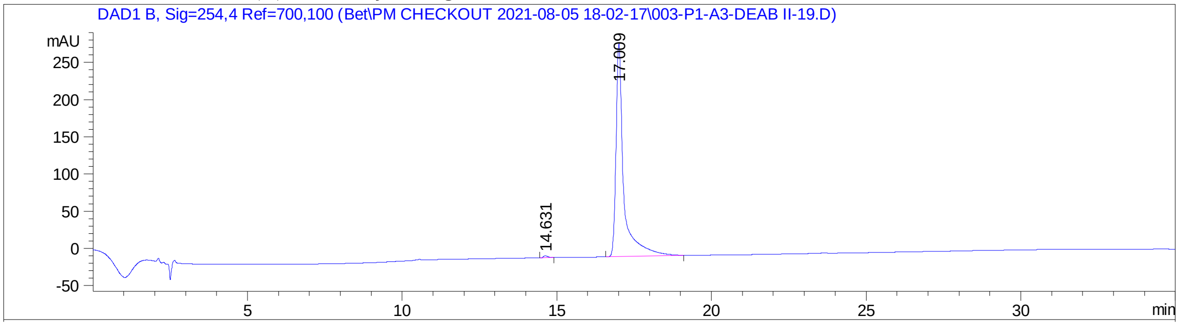

```
Signal 1: DAD1 B, Sig=254,4 Ref=700,100

Peak RetTime Type  Width     Area      Height     Area
  #   [min]        [min]   [mAU*s]     [mAU]        %
----|-------|----|-------|----------|----------|--------|
   1  14.631 BB    0.1441   23.97947    2.49540   0.4800
   2  17.009 BB    0.2491 4971.85547  285.60602  99.5200
```

4-(diethylamino)-3-methoxybenzaldehyde (20) :

Additional Info : Peak(s) manually integrated

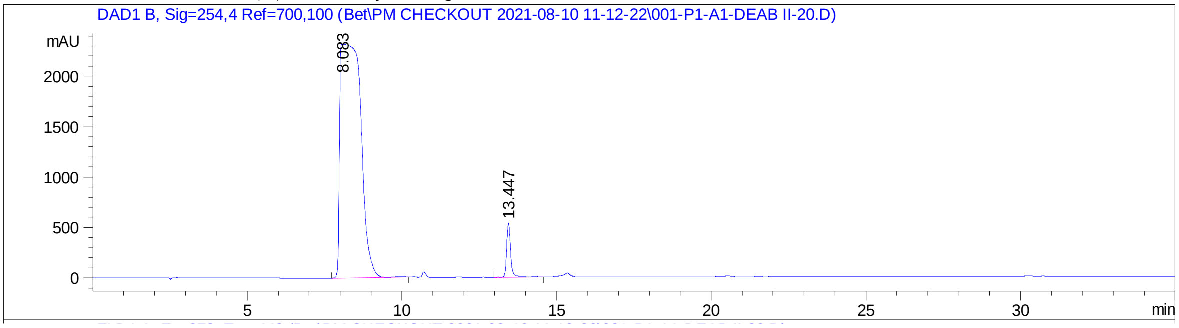


```
Signal 1: DAD1 B, Sig=254,4 Ref=700,100

Peak RetTime Type  Width     Area      Height     Area
  #   [min]        [min]   [mAU*s]     [mAU]        %
----|-------|----|-------|----------|----------|--------|
   1   8.083 BV R  0.5795 1.08581e5  2318.48730  95.7919
   2  13.447 BV R  0.1354 4769.98145  533.38348   4.2081
```

3-bromo-4-(4-methylpiperazin-1-yl)benzaldehyde (21) :

Additional Info : Peak(s) manually integrated

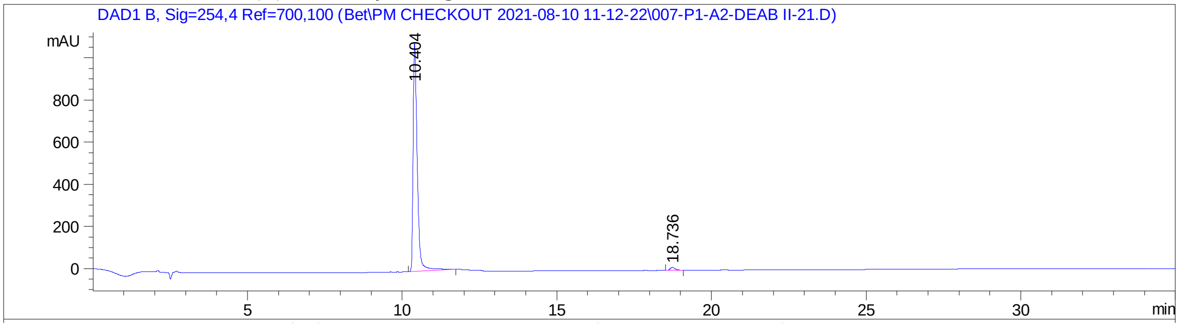


```
Signal 1: DAD1 B, Sig=254,4 Ref=700,100

Peak RetTime Type  Width     Area      Height     Area
  #   [min]        [min]   [mAU*s]     [mAU]        %
----|-------|----|-------|----------|----------|--------|
   1  10.404 BB    0.1415 1.01525e4  1081.52869  98.4948
   2  18.736 BB    0.1636  155.15344   14.40665   1.5052
```

3-bromo-4-morpholinobenzaldehyde (22) :

Additional Info : Peak(s) manually integrated

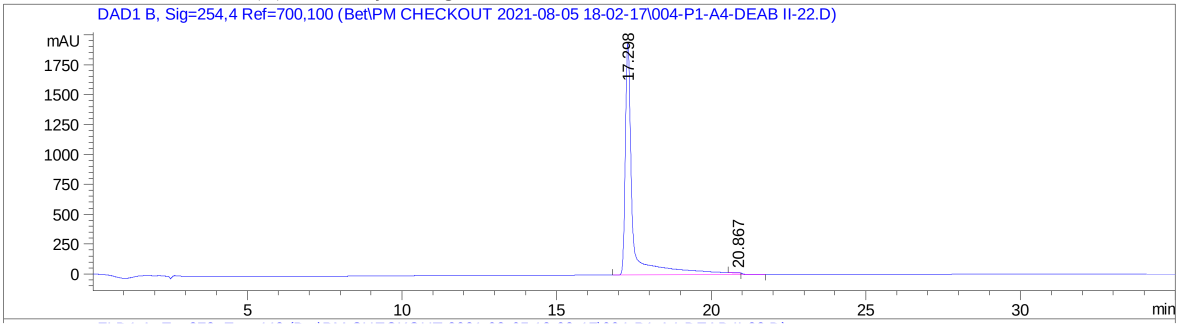

Signal 1: DAD1 B, Sig=254,4 Ref=700,100

Peak #	RetTime [min]	Type	Width [min]	Area [mAU*s]	Height [mAU]	Area %
1	17.298	BV R	0.2446	3.29267e4	1934.66089	99.8757
2	20.867	VB E	0.1624	40.97242	4.25174	0.1243

3-bromo-4-(piperidin-1-yl)benzaldehyde (23) :

Additional Info : Peak(s) manually integrated

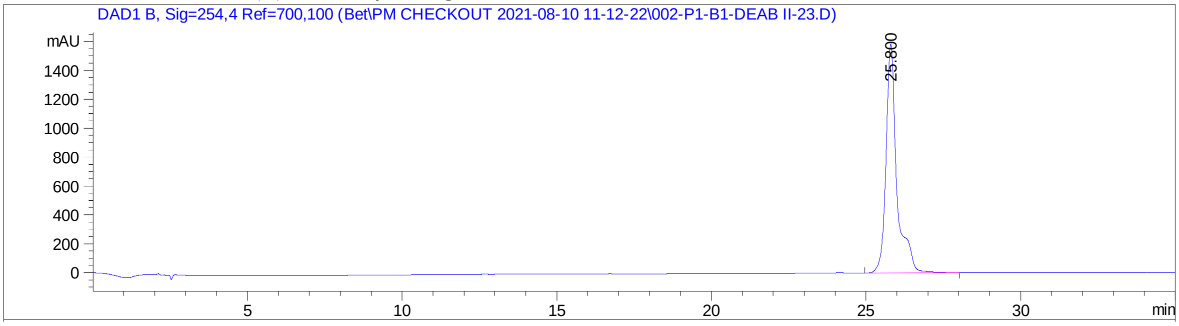

Signal 1: DAD1 B, Sig=254,4 Ref=700,100

Peak #	RetTime [min]	Type	Width [min]	Area [mAU*s]	Height [mAU]	Area %
1	25.800	BB	0.3578	3.88174e4	1584.67700	100.0000

3-methyl-4-(4-methylpiperazin-1-yl)benzaldehyde (24) :

Additional Info : Peak(s) manually integrated

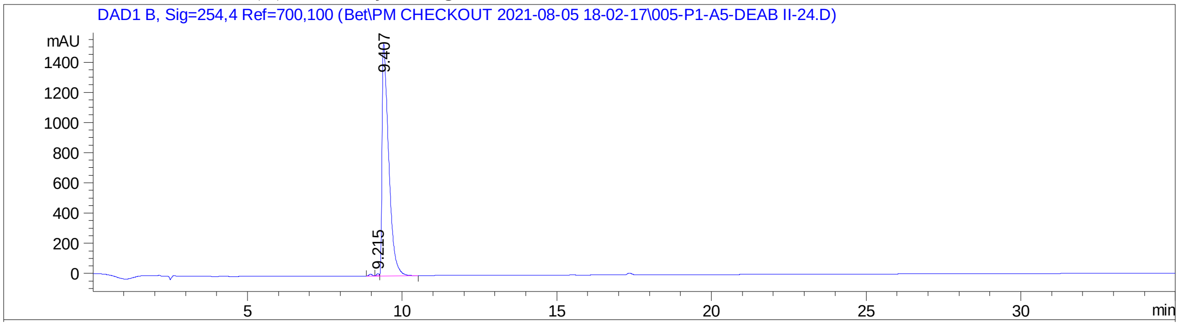

Signal 1: DAD1 B, Sig=254,4 Ref=700,100

Peak #	RetTime [min]	Type	Width [min]	Area [mAU*s]	Height [mAU]	Area %
1	9.215	VV E	0.0858	84.46116	15.45619	0.3658
2	9.407	VB R	0.2281	2.30029e4	1535.69080	99.6342

4-(4-methylpiperazin-1-yl)-3-nitrobenzaldehyde (25) :

Additional Info : Peak(s) manually integrated

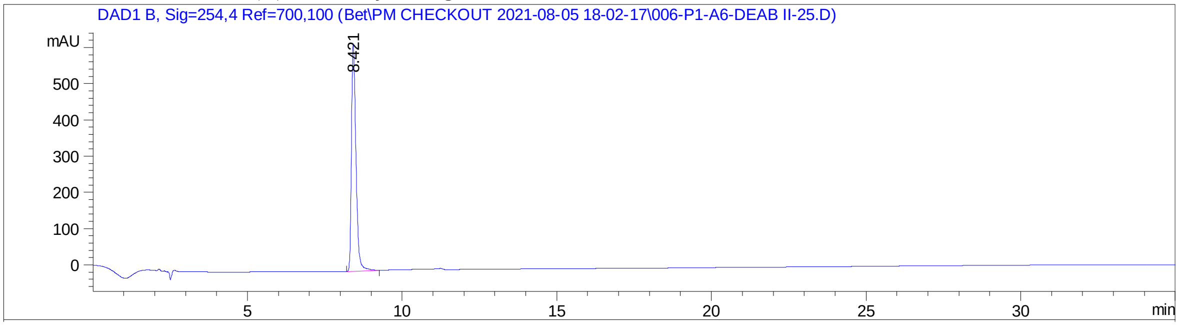

Signal 1: DAD1 B, Sig=254,4 Ref=700,100

Peak #	RetTime [min]	Type	Width [min]	Area [mAU*s]	Height [mAU]	Area %
1	8.421	BB	0.1562	6351.05127	626.62372	100.0000

3-methyl-4-(pyrrolidin-1-yl)benzaldehyde (26) :

Additional Info : Peak(s) manually integrated

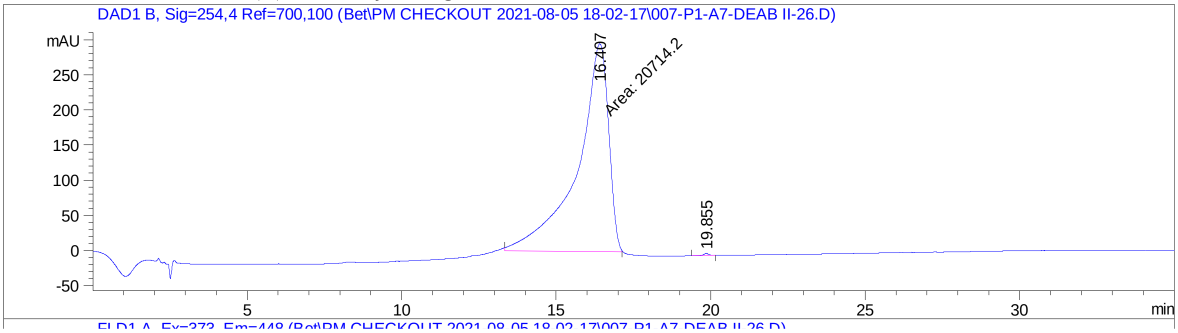

Signal 1: DAD1 B, Sig=254,4 Ref=700,100

Peak #	RetTime [min]	Type	Width [min]	Area [mAU*s]	Height [mAU]	Area %
1	16.407	MM	1.1679	2.07142e4	295.60068	99.8284
2	19.855	BB	0.1767	35.59895	2.99463	0.1716

3-methyl-4-morpholinobenzaldehyde (27) :

Additional Info : Peak(s) manually integrated

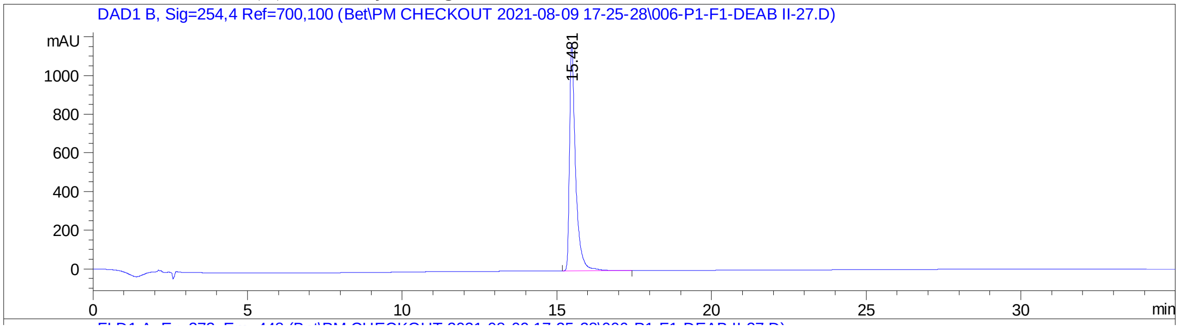

Signal 1: DAD1 B, Sig=254,4 Ref=700,100

Peak #	RetTime [min]	Type	Width [min]	Area [mAU*s]	Height [mAU]	Area %
1	15.481	BB	0.1967	1.59835e4	1174.73547	100.0000

3-methoxy-4-(piperidin-1-yl)benzaldehyde (28) :

Additional Info : Peak(s) manually integrated

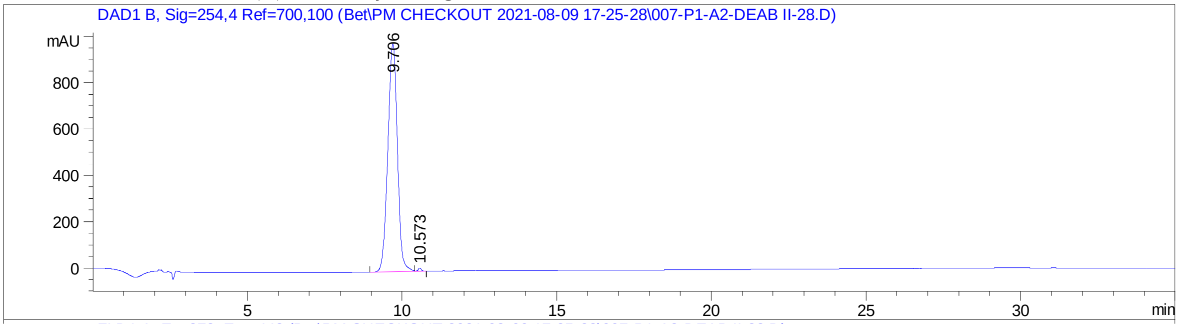

Signal 1: DAD1 B, Sig=254,4 Ref=700,100

Peak #	RetTime [min]	Type	Width [min]	Area [mAU*s]	Height [mAU]	Area %
1	9.706	BV R	0.3372	2.11668e4	983.10510	99.5441
2	10.573	VB E	0.1053	96.94885	14.25661	0.4559

3-methoxy-4-(pyrrolidin-1-yl)benzaldehyde (29) :

Additional Info : Peak(s) manually integrated

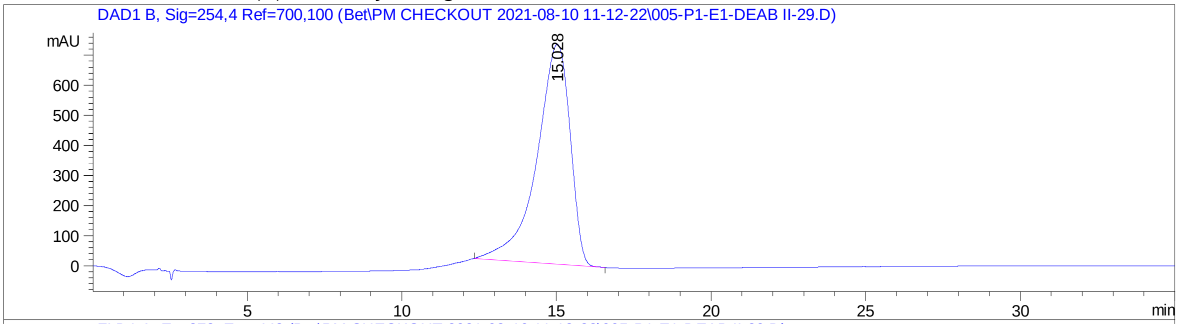

Signal 1: DAD1 B, Sig=254,4 Ref=700,100

Peak #	RetTime [min]	Type	Width [min]	Area [mAU*s]	Height [mAU]	Area %
1	15.028	BB	1.1462	5.47831e4	731.07794	100.0000

3-methoxy-4-(4-methylpiperazin-1-yl)benzaldehyde (30) :

Additional Info : Peak(s) manually integrated

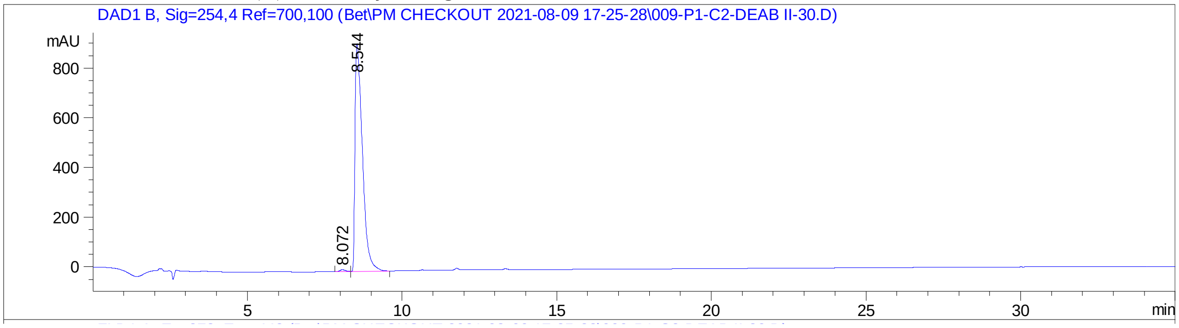

Signal 1: DAD1 B, Sig=254,4 Ref=700,100

Peak #	RetTime [min]	Type	Width [min]	Area [mAU*s]	Height [mAU]	Area %
1	8.072	BV	0.2051	96.92163	7.48186	0.6336
2	8.544	VB	0.2640	1.51992e4	914.25690	99.3664

3-methoxy-4-morpholinobenzaldehyde (31) :

Additional Info : Peak(s) manually integrated

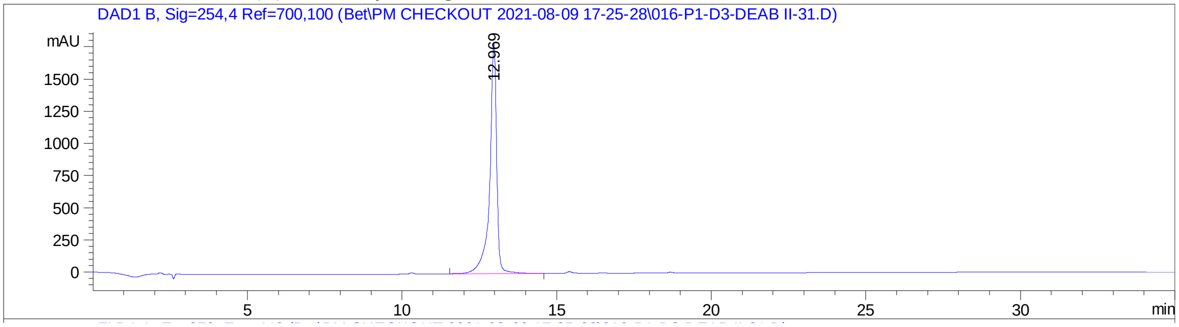

Signal 1: DAD1 B, Sig=254,4 Ref=700,100

Peak #	RetTime [min]	Type	Width [min]	Area [mAU*s]	Height [mAU]	Area %
1	12.969	BB	0.2303	2.82186e4	1786.05750	100.0000

4-morpholino-3-nitrobenzaldehyde (32) :

Additional Info : Peak(s) manually integrated

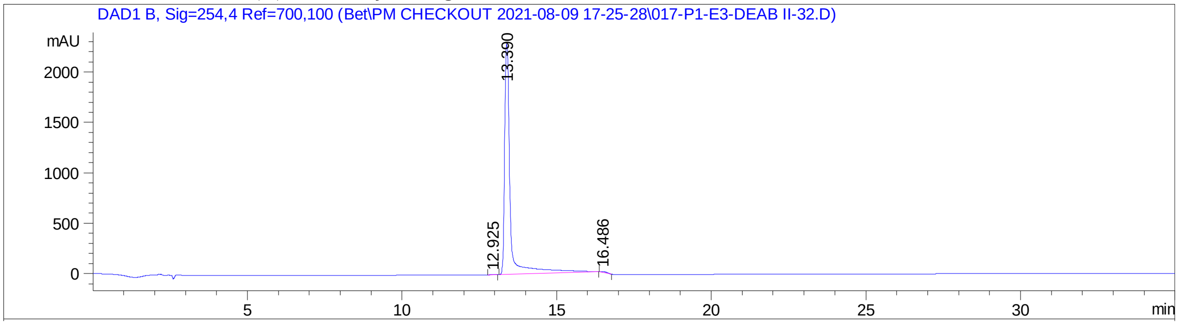

Signal 1: DAD1 B, Sig=254,4 Ref=700,100

Peak #	RetTime [min]	Type	Width [min]	Area [mAU*s]	Height [mAU]	Area %
1	12.925	BB	0.1187	11.98015	1.57544	0.0410
2	13.390	BB	0.1905	2.91033e4	2286.96753	99.5081
3	16.486	BB	0.2636	131.87599	7.70997	0.4509

4-isopropoxy-3-methoxybenzaldehyde (33) :

Additional Info : Peak(s) manually integrated

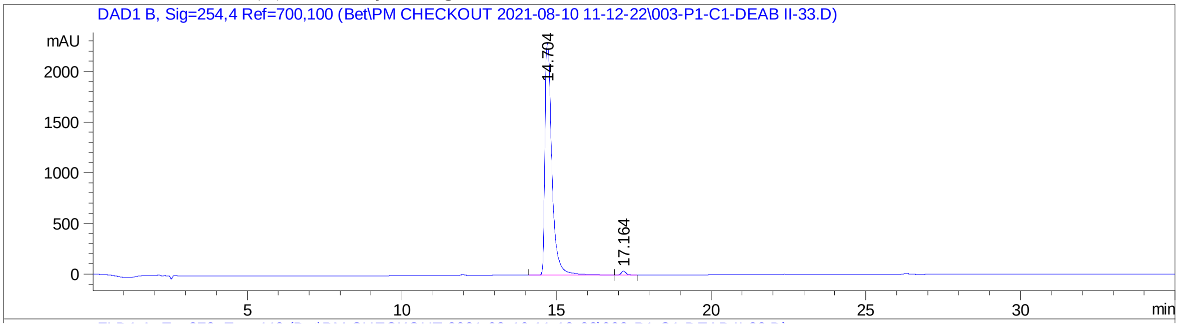

Signal 1: DAD1 B, Sig=254,4 Ref=700,100

Peak #	RetTime [min]	Type	Width [min]	Area [mAU*s]	Height [mAU]	Area %
1	14.704	BB	0.2382	3.60028e4	2280.67334	98.9063
2	17.164	BB	0.1580	398.10287	38.68860	1.0937

3-nitro-4-(pyrrolidin-1-yl)benzaldehyde (34) :

Additional Info : Peak(s) manually integrated

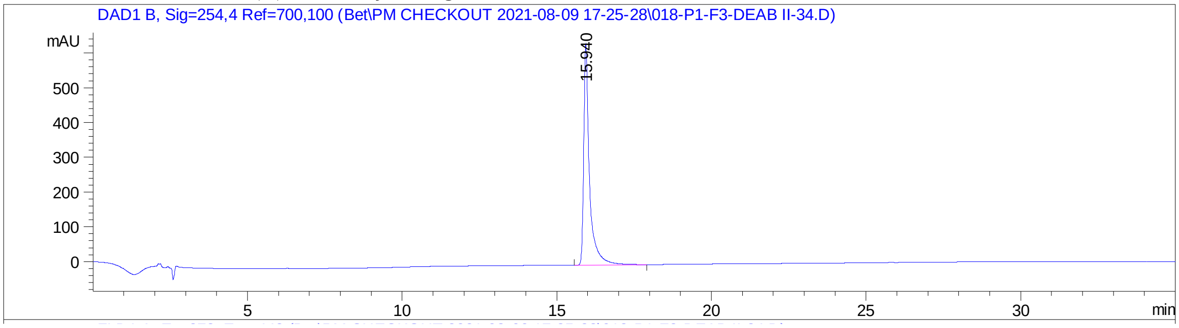

Signal 1: DAD1 B, Sig=254,4 Ref=700,100

Peak #	RetTime [min]	Type	Width [min]	Area [mAU*s]	Height [mAU]	Area %
1	15.940	BB	0.1873	8238.16016	635.28925	100.0000

4-isopropoxy-3-methoxy-5-nitrobenzaldehyde (35) :

Additional Info : Peak(s) manually integrated

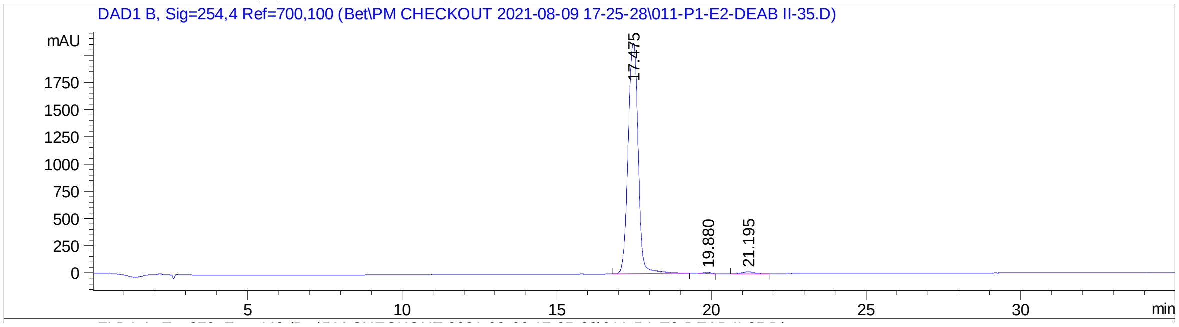

Signal 1: DAD1 B, Sig=254,4 Ref=700,100

Peak #	RetTime [min]	Type	Width [min]	Area [mAU*s]	Height [mAU]	Area %
1	17.475	BB	0.3562	4.78470e4	2112.57935	98.9476
2	19.880	BB	0.1845	132.94029	10.73396	0.2749
3	21.195	BB	0.3332	375.95752	17.60258	0.7775

3-nitro-4-(piperidin-1-yl)benzaldehyde (36) :

Additional Info : Peak(s) manually integrated

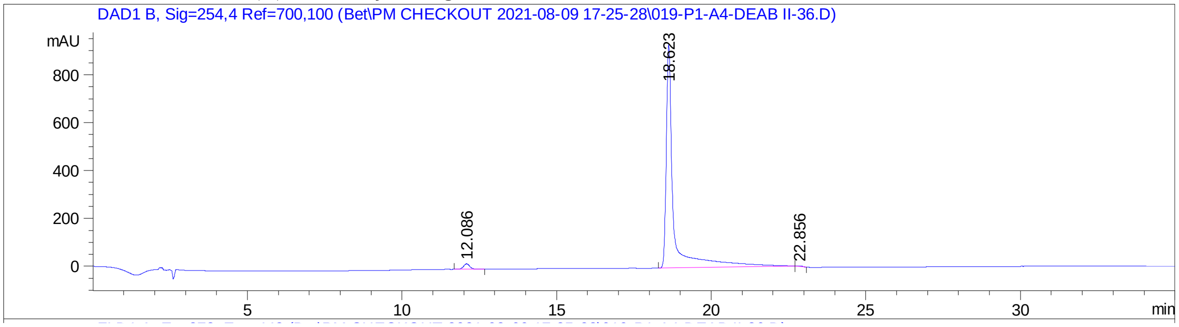

Signal 1: DAD1 B, Sig=254,4 Ref=700,100

Peak #	RetTime [min]	Type	Width [min]	Area [mAU*s]	Height [mAU]	Area %
1	12.086	BB	0.2082	312.70349	22.77236	1.9848
2	18.623	BB	0.2299	1.54125e4	937.18359	97.8268
3	22.856	BB	0.1845	29.67567	2.53718	0.1884

4-(diethylamino)-2-methoxybenzaldehyde (37) :

Additional Info : Peak(s) manually integrated

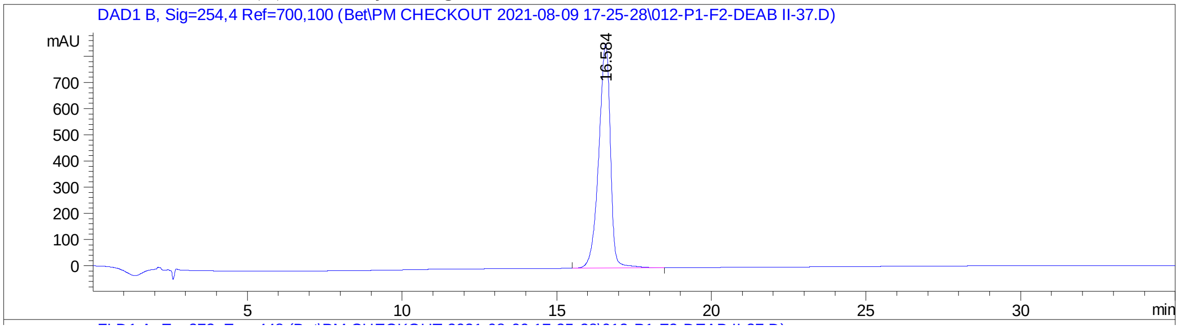

Signal 1: DAD1 B, Sig=254,4 Ref=700,100

Peak #	RetTime [min]	Type	Width [min]	Area [mAU*s]	Height [mAU]	Area %
1	16.584	BB	0.4016	2.24093e4	855.60120	100.0000

4-(dipropylamino)-2-methoxybenzaldehyde (38) :

Additional Info : Peak(s) manually integrated

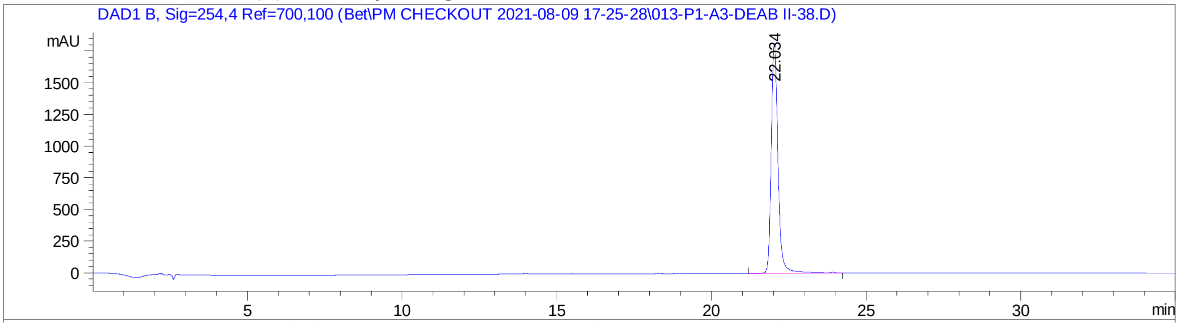

Signal 1: DAD1 B, Sig=254,4 Ref=700,100

Peak #	RetTime [min]	Type	Width [min]	Area [mAU*s]	Height [mAU]	Area %
1	22.034	BV R	0.2316	2.76324e4	1809.83557	100.0000

***tert*-butyl 4-(4-formyl-2-nitrophenyl)piperazine-1-carboxylate (39) :**

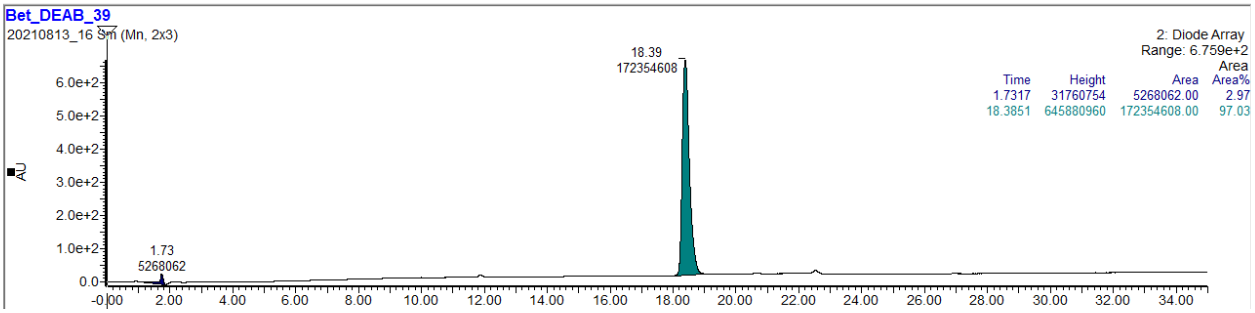

3-nitro-4-(piperazin-1-yl)benzaldehyde (40) :

Additional Info : Peak(s) manually integrated

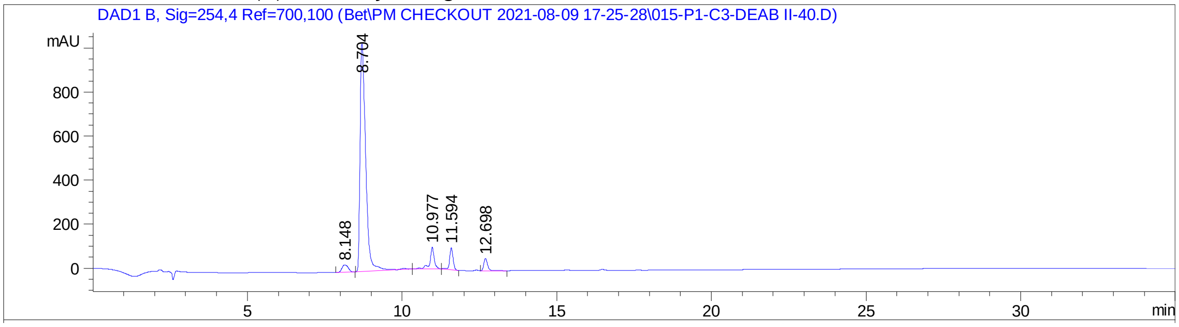

Signal 1: DAD1 B, Sig=254,4 Ref=700,100

Peak #	RetTime [min]	Type	Width [min]	Area [mAU*s]	Height [mAU]	Area %
1	8.148	BB	0.2300	478.32361	34.03717	3.0088
2	8.704	BV R	0.1982	1.31731e4	1032.36182	82.8622
3	10.977	VB R	0.1216	985.56915	99.70602	6.1995
4	11.594	VB R	0.1143	783.38550	101.05767	4.9277
5	12.698	VV R	0.1277	477.22284	55.52624	3.0019
